# How Reliable Are Modern Density Functional Approximations to Simulate Vibrational Spectroscopies?

**DOI:** 10.1021/acs.jpclett.2c01278

**Published:** 2022-06-23

**Authors:** Sebastian P. Sitkiewicz, Robert Zaleśny, Eloy Ramos-Cordoba, Josep M. Luis, Eduard Matito

**Affiliations:** † Donostia International Physics Center (DIPC), 20018 Donostia, Euskadi, Spain; ‡ Polimero eta Material Aurreratuak: Fisika, Kimika eta Teknologia, Kimika Fakultatea, Euskal Herriko Unibertsitatea UPV/EHU, P.K. 1072, 20080 Donostia, Euskadi, Spain; ¶ Faculty of Chemistry, Wrocław University of Science and Technology, Wyb. Wyspiańskiego 27, PL−50370 Wrocław, Poland; § Institut de Química Computacional i Catàlisi (IQCC) and Departament de Química, Universitat de Girona, 17003 Girona, Catalonia, Spain; ∥ Ikerbasque Foundation for Science, Plaza Euskadi 5, 48009 Bilbao, Euskadi, Spain

## Abstract

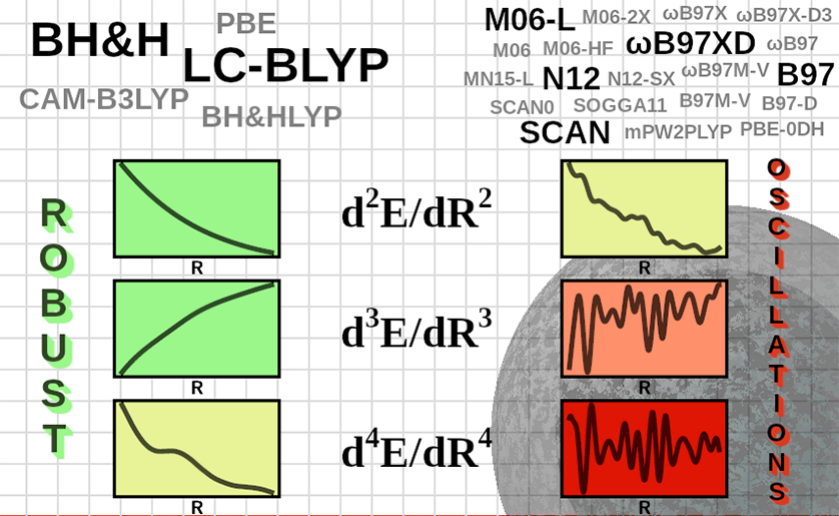

We show that properties of molecules with low-frequency modes calculated with density functional approximations (DFAs) suffer from spurious oscillations along the nuclear displacement coordinate due to numerical integration errors. Occasionally, the problem can be alleviated using extensive integration grids that compromise the favorable cost-accuracy ratio of DFAs. Since spurious oscillations are difficult to predict or identify, DFAs are exposed to severe performance errors in IR and Raman intensities and frequencies or vibrational contributions to any molecular property. Using Fourier spectral analysis and digital signal processing techniques, we identify and quantify the error due to these oscillations for 45 widely used DFAs. LC-BLYP and BH&H are revealed as the only functionals showing robustness against the spurious oscillations of various energy, dipole moment, and polarizability derivatives with respect to a nuclear displacement coordinate. Given the ubiquitous nature of molecules with low-frequency modes, we warrant caution in using modern DFAs to simulate vibrational spectroscopies.

Given its effectiveness, low cost, and conceptual simplicity, Kohn–Sham density functional theory has become the true workhorse of modern computational chemistry.^1−3^ Since the exact density functional is unknown, a race for constructing the most accurate and versatile density functional approximation (DFA) is still on. The three most significant challenges of modern DFAs,^4,5^ the delocalization error,^6^ dispersion forces,^7^ and strong correlation,^2^ occupy the agenda of DFA developers, who have lately put forward various approximations that, to some extent, succeeded in overcoming these limitations.^8,9^ The increase of accuracy often comes hand-in-hand with the mathematical complexity of modern DFAs. Unlike most wave function methods, the computation of electronic energies from DFAs requires the numerical integration of the exchange and correlation functionals, controlled by the size and type of the numerical integration grid. Johnson and co-workers showed that spurious oscillations appear in the potential energy surface (PES) of dissociating dispersion-bounded systems calculated with meta generalized gradient approximations (meta-GGAs) using small integration grids.^10,11^ The origin of these oscillations was attributed to an insufficient grid sampling of the midpoints in between the dispersion-bonded atoms, where the kinetic energy density has greatly enhanced values.^11^ Other authors have identified similar spurious oscillations on other molecules stabilized by London dispersion forces.^5,10−20^ However, thus far, the problem seemed to be limited to the computation of PES of dispersion complexes with meta-GGAs and solved by the use of reasonably large grids [such as (250,590), see below].

In the present work, we show that the spurious oscillations that arise from the sizable numerical integration errors of DFAs are neither a problem limited to meta-GGAs and the PES of dispersion compounds nor can be solved using the largest predefined grids available in most computational packages. An earlier study by some of the present authors reported enormous errors in anharmonic vibrational corrections to electric properties using some DFAs.^21^ A subsequent in-depth analysis of these errors motivated the present Letter. As it will be demonstrated in this work, these spurious oscillations occur in molecules with low-frequency vibrational modes, affect various molecular properties, and vary with the grid size, making most DFA predictions strongly grid-size dependent. The errors derived from this grid-dependency problem are sometimes huge and can be easily spotted,^21^ whereas in many other cases are disguised as performance errors attributed to the approximate nature of the functional. In fact, the oscillations significantly affect energy derivatives with respect to nuclear displacements and can occur in all kinds of DFAs. Avoiding them is not always possible and requires grid sizes that compromise the cost-efficiency of DFAs. As a result, the calculation of fundamental properties related to the vibrational motion of molecular systems poses a greater challenge to DFAs than it was initially anticipated.

First of all, let us show that the problem affects various energy derivatives and it cannot always be solved increasing the numerical grid size. Figure 1 illustrates the spurious oscillations of various energy derivatives with respect to the interatomic distance in Ar_2_ calculated with three popular DFAs. Although these oscillations were not evident from the potential energy curve of Ar_2_,^11,19,20^ they compromise the calculation of accurate force constants and higher-order derivatives, for which the oscillations intensify, leading to great discrepancies in response properties that involve derivatives with respect to the nuclear coordinates. In some cases, at the expense of a large computational cost, the problem can be partially alleviated if grids much larger than those predefined in standard packages are employed. However, for some DFAs (see M06-2X and ωB97X), the oscillations persist after increasing the grid size, and obtaining an accurate response property is out of reach even with extremely large integration grids. Since one cannot either anticipate whether there is a grid size sufficient to avoid the spurious oscillations or which DFAs will present this problem, one is exposed to serious performance errors in the calculation of various response properties such as IR and Raman intensities and frequencies and zero-point vibrational averages or pure vibrational contributions to any molecular property. The specific goals of this work are (i) bring attention to this shortcoming of many modern DFAs, (ii) put forward a method to easily identify and quantify the error due to these spurious oscillations, (iii) do an exploration of the grid-size dependency of the most popular DFAs, and (iv) identify which functionals can be safely used to calculate various response properties.

**Figure 1:**
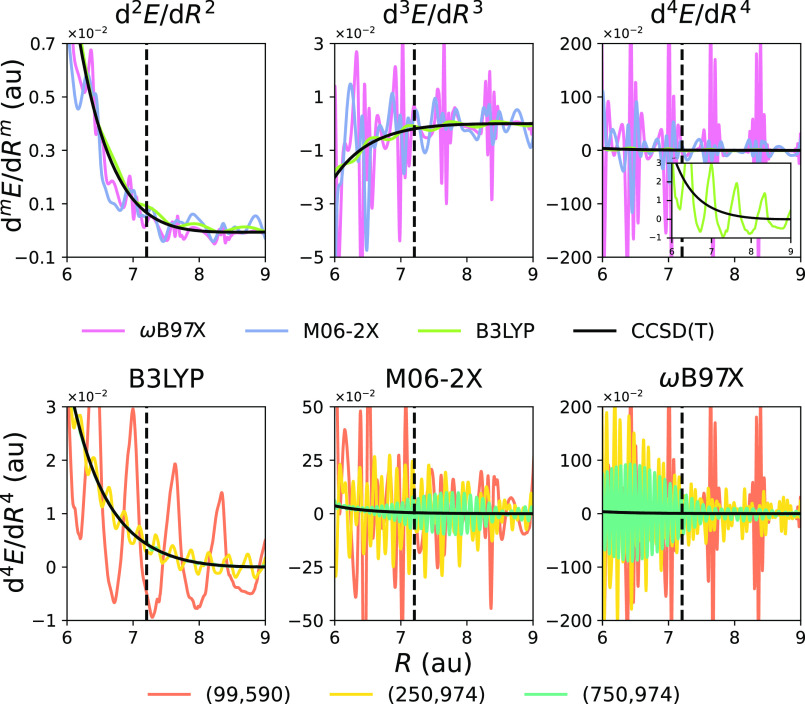
Grid-related spurious oscillations affecting the derivatives of total energy with respect to interatomic separation *R* of Ar_2_, d^*m*^*E*/d*R*^*m*^ (*m* = 2–4). Top-row panels show the results of DFAs combined with the (99,590) grid. On the bottom row panels, curves of d^4^*E*/d*R*^4^ obtained with various integration grids are shown. In all plots, the black solid curve represents the CCSD(T) results and the vertical lines mark the equilibrium distance. All units are a.u.

We will analyze the derivatives of the total energy, d^*m*^*E*/dξ^*m*^ (*m* = 1–4), the dipole moment, d^*m*^μ_*z*_/dξ^*m*^ (*m* = 1–3), and the static polarizability, d^*m*^α_*zz*_/dξ^*m*^ (*m* = 1–2), with respect to the nuclear displacement coordinate (ξ) of dispersion-bonded complexes (Ar_2_ and He_2_), hydrogen-bonded (HCN···HF, HCN···HCl, CO···HF, and N_2_···HF), and halogen-bonded (HCN···BrH and HCN···BrF) complexes. We investigated 45 DFAs including generalized gradient approximations (GGAs), meta-GGAs, global hybrids, range-separated DFAs, and double hybrids (see the figures below for the full list). In all cases, the aug-cc-pVTZ basis set was used. We tested various integration grids: (99,590) (i.e., unpruned SG3^22^ and UltraFine^23^), (250,974) (i.e., unpruned SuperFineGrid^23^), (500,974), and (750,974), where (*N*_r_, *N*_Ω_) indicates *N*_r_ radial shells and *N*_Ω_ angular points per atom. The unpruned (250,974) grid is already larger than most of the largest predefined grids in standard computational packages (see Table S3). The cost of the calculations multiply by four going from (99,590) to (250,974) and triple from (250,974) to (750,974). Hence, the calculation with the latter grid increases by more than 1 order of magnitude the cost of most calculations performed nowadays. For diatomic molecules, we scanned the properties along the interatomic bond distance, whereas in polyatomic systems, we represent the nuclear displacements (ξ) with the first-order field-induced coordinate (FIC), χ_1,*z*_. The first-order FIC is a linear combination of normal modes, which effectively represents an overall response of the equilibrium geometry to an external electric field (see eq S1 for further details).^24^

We find two difficulties to establish a spurious-oscillation-free reference that we can use to quantify the distortion of the property along the nuclear displacement. First, we cannot directly compare the DFA and another computational method because the differences could be either due to the spurious oscillations or to the methods’ performance. Hence, the reference must be based on the same DFA. However, we have also established (see Figure 1) that we cannot blindly trust a very large grid. To circumvent these problems, we will benefit from the fact that the spurious oscillations depicted in Figure 1 show regular patterns. We have designed an algorithm (sketched in Figure 2, see section 1.2 of the Supporting Information for further details) that employs Fourier spectral analysis and digital signal processing to identify and quantify the oscillations of a property measured with a given DFA, *P*^DFA^. The Fourier spectra of *P*^DFA^ and *P*^ref^, an integration-error-free reference, usually obtained from *ab initio* calculations such as HF or CCSD, are compared to identify spurious oscillations. The latter are removed from *P*^DFA^, giving rise to *P*_filt_^DFA^, which can now be compared against *P*^DFA^. In order to quantify how the oscillations affect the property, we calculate the root-mean-square (RMS) of a set of points (close to the equilibrium geometry) along the property profile. The ratio between the oscillation and the property RMSs gives the relative root-mean-square error (RRMSE),
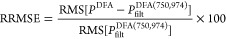
1Large RRMSE values indicate significant spurious oscillations and warn about the presence of potentially large errors in various molecular properties. RRMSE is a convenient measure of the spurious oscillations because it only requires the computation of the derivative profile at the DFA level and a cost-efficient *ab initio* reference such as HF. d^*m*^*E*/dξ^*m*^, d^*m*^μ_*z*_/dξ^*m*^, and d^*m*^α_*zz*_/dξ^*m*^ enter the expression of various vibrational spectroscopic quantities; however, it is difficult to anticipate the effect of the spurious oscillations on these molecular properties because they depend on various derivatives in quite different ways. In Table S5, we review the connection between these derivatives and various molecular properties. Since we are quantifying the effect of the spurious oscillations on the derivative profile with RRMSE, we can also use it to predict how large the error in the properties might be. To this end, in Table S16, we collect RRMSE values and relative errors for various vibrational spectroscopic properties of N_2_·HF. The numbers show that the relative errors in the property might be as large as twice the RRMSE value. For instance, 17% RRMSE on dα_*zz*_/dξ gives a relative error on the harmonic Raman intensity of 38%; 64% RRMSE on d^2^α_*zz*_/dξ^2^ gives a relative error on the anharmonic correction to the Raman intensity of 163%. Certain properties depend on more than one derivative, and the RRMSE of all the derivatives need to be considered. For instance, RRMSEs of 16% and 665% for d^3^*E*/dξ^3^ and d^4^*E*/dξ^4^, respectively, must be considered to explain the 234% error on the calculation of IR anharmonic corrections to the intermolecular stretching mode of N_2_·HF using ωB97X with the (250,974) grid.

**Figure 2:**
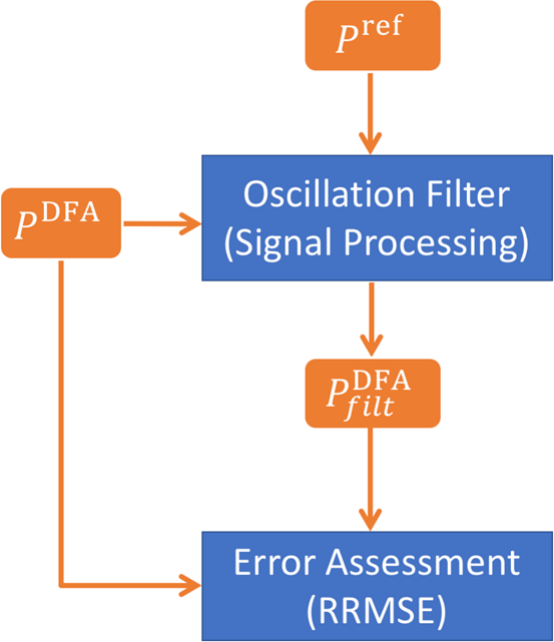
Flowchart of the algorithm to quantify the spurious oscillations. *P*^DFA^ is the property profile calculated with the target DFA. *P*^ref^ is a integration-error-free property profile, usually obtained from an *ab initio* wave function method. *P*_filt_^DFA^ is the property profile calculated with the target DFA after filtering the spurious oscillations.

In Figure 3, for a representative set of 11 DFAs, we collect the maximum RRMSE values within the set of studied systems (see Figures S9–S17 for the full list of DFAs). RRMSE can reach thousands of percents and, as expected, the largest errors correspond to high-order derivatives. The (99,590) grid, which is equal or larger than the largest predefined grid in QCHEM 5.3^22^ or ORCA 5.0.1,^25^ gives maximum RRMSE values that exceed 10%, 40%, and 500%, respectively, for d^2^*E*/dξ^2^, d^3^*E*/dξ^3^, and d^4^*E*/dξ^4^. The latter is a clear indication that (99,590) is definitely an insufficient grid to compute vibrational spectroscopic data of molecules with at least a low-frequency vibrational mode. For the (250,974) grid, the maximum values of RRMSE for d^4^*E*/dξ^4^ are greater than 160% for all DFAs, except for BH&H (18%) and LC-BLYP (11%). The largest grid, (750,974), reduces the maximum RRMSE significantly for the first and second energy derivatives but some popular DFAs, such as ωB97X, SCAN, or M06-2X, give extremely large errors for the third and fourth derivatives; in fact, even with the largest grid, only a handful of functionals gives a maximum RRMSE below 50% for d^4^*E*/dξ^4^.

**Figure 3:**
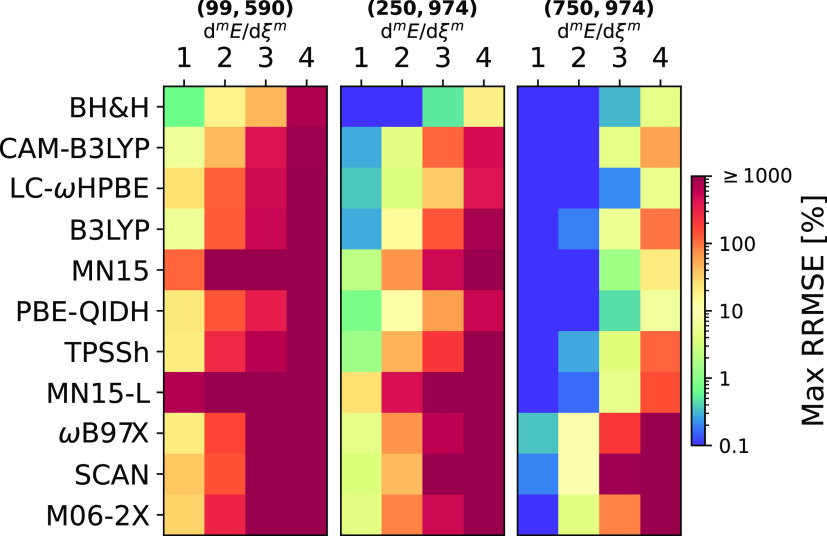
Maximum values of RRMSE for the derivatives of total energy within the set of all studied molecular complexes.

We set RRMSE below 10% as the criterion to define a *safe* calculation (thorough numerical tests indicate that property errors due to spurious oscillations are usually below 20% for such RRMSE values). Modern DFAs with the (99,590) integration grid, which is the default choice in many computational packages,^22,23^ cannot be safely used to study the IR or Raman spectroscopy of molecules involving floppy motions (see Figure S18). In Figure 4, we collect the maximum order of the derivatives which can be safely calculated using a wide range of DFAs employing the (250,974) grid. Derivatives up to the fourth, third, and second order along the nuclear displacement were considered for the energy and the main symmetry axis component of the dipole and first polarizability, respectively. The molecules tested are divided into three groups: hydrogen-bonded (A), halogen-bonded (B), and dispersion-bonded (C) molecules. Hydrogen-bonded complexes show the smallest spurious oscillations, although they are still considerably large. Halogen-bonded molecules exhibit much more significant oscillations, whereas dispersion-bonded systems are the species most affected by this problem. We have classified the DFAs into five rungs according to their overall stability across various molecular properties (see section S1.3 of the Supporting Information for further details about this classification). Within the same group of molecules, the magnitude of the spurious oscillations increases in the following order: d^*m*^*E*/dξ^*m*^, d^*m*^μ_*z*_/dξ^*m*^, and d^*m*^α_*zz*_/dξ^*m*^. One should take into account that molecules in group C are centrosymmetric; hence, d^*m*^μ_*z*_/dξ^*m*^ were not tested for the most challenging molecules of the set.

**Figure 4:**
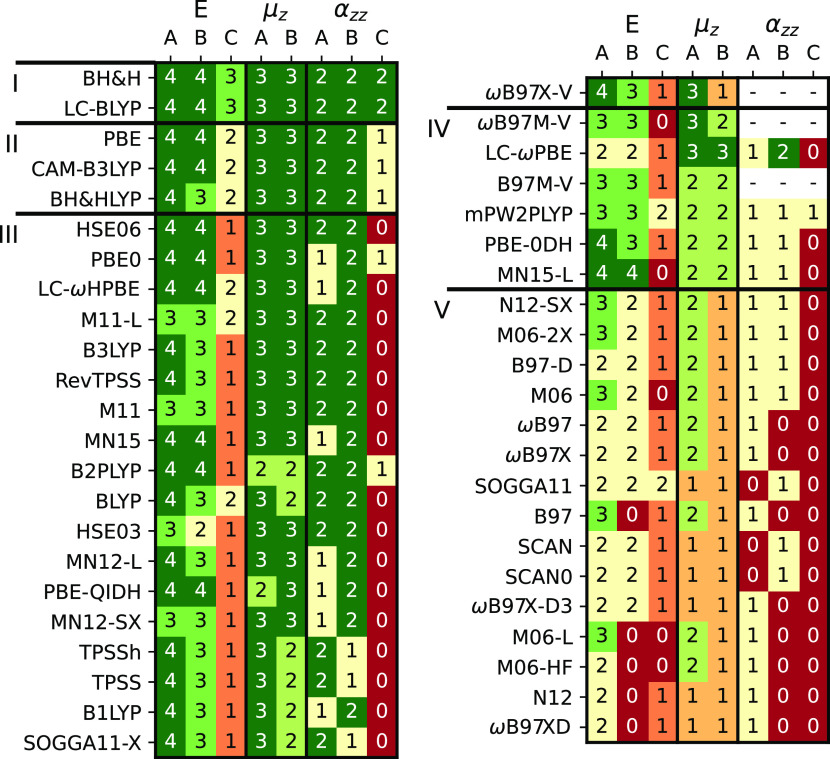
Maximum order of derivatives that can be safely obtained (i.e., RRMSE ≤ 10%) with the (250,974) grid. Groups A, B, and C, correspond to hydrogen-, halogen-, and dispersion-bonded complexes, respectively. Data not available for ωB97X-V, ωB97M-V, and B97M-V, for which static polarizabilities are not implemented. Figures S18 and S19 collect the same information for grids (99,590) and (750,974).

*Rung I* includes grid-robust DFAs, namely, BH&H and LC-BLYP. These DFAs provide safe results for almost all derivatives using the (250,974) grid. BH&H is the only functional in our set which does not employ a GGA or meta-GGA exchange. In practice, BH&H is seldom employed, because it is not accurate for most chemical purposes. (This DFA is not even considered in two of the most extensive recent benchmark studies.^5,26^) Conversely, LC-BLYP, has been extensively used to study molecular properties,^21,27−29^ and, hence, it is considered the safest choice among the DFAs covered in this study.

DFAs in *Rung II*, PBE, CAM-B3LYP, and BH&HLYP, suffer from some grid dependency. They can provide stable anharmonic vibrational corrections for the hydrogen- and halogen-bonded complexes and harmonic contributions for the dispersion-bonded systems. On the contrary, they should not be employed to calculate anharmonic contributions of dispersion-bonded systems.

*Rung III* includes DFAs suffering from grid-dependent oscillations for all the studied systems. Widely used DFAs, such as B3LYP, PBE0, MN15, and ωB97X-V, belong to this group. The DFAs in this rung cannot provide stable force constants or the Raman intensities (see dα_*zz*_/dξ) of dispersion-bonded systems.

*Rung IV* encloses DFAs which are highly grid dependent. Most of these DFAs encounter serious difficulties to study molecules with low-frequency vibrations, and within this group of molecules, they are limited to the basic anharmonic corrections to frequencies and IR intensities (see d^3^*E*/dξ^3^ and d^2^μ_*z*_/dξ^2^) of hydrogen- and halogen-bonded complexes. We advise to use these DFAs with a great caution, as they may require giant integration grids to avoid spurious oscillations (see Figure S19).

DFAs with the highest grid dependency belong to *Rung V*. This group consists mostly of DFAs from the B97, M06, SCAN, and N12 families, including very popular DFAs such as M06-2X and ωB97X. Within the set of molecules exhibiting low-frequency vibrations, most of *Rung V* DFAs are limited to the calculation of harmonic vibrational properties of hydrogen- and halogen-bonded complexes. Some DFAs, namely, B97, M06-L, M06-HF, N12, and ωB97XD, cannot provide stable energy or polarizability gradients for the halogen-bonded systems. Grids smaller than (250,974) seriously compromise the calculation of energy gradients even for hydrogen-bonded complexes (see Figure S18). A much larger grid, such as (750,974), increases the stability of some DFAs in *Rung V*, but even with this enormous grid, these DFAs cannot be trusted to calculate harmonic frequencies of dispersion-bonded complexes (see Figure S19).

In summary, we have uncovered an important limitation of most modern density functional approximations, which might suffer from spurious oscillations of molecular properties along the nuclear displacement coordinate. The oscillations are due to numerical integration errors, which can be alleviated using large integration grids that compromise the favorable cost-accuracy ratio of DFAs. In some cases, the spurious oscillations cannot be avoided even using enormous grids such as (750,974).

Using Fourier spectral analysis and digital signal processing techniques we can easily identify and quantify these oscillations. We have used an algorithm to classify forty-five popular DFAs into five groups. Among widely employed DFAs, only LC-BLYP shows robustness against spurious oscillations of various molecular properties examined along a nuclear displacement coordinate. Hardly a handful of DFAs is safe to compute basic anharmonic corrections to the vibrational properties of molecules with low-frequency modes. Long-range corrected DFAs (LC-DFAs) reduce the amount of GGA exchange, prone to numerical integration errors, at large interelectronic separations. Hence, LC-DFAs are less sensitive to spurious oscillations than their uncorrected counterparts. The latter statement is supported by the different performance of LC-BLYP (CAM-B3LYP) and BLYP and (B3LYP).

All the molecules presented in this Letter are noncovalently bonded complexes, which especially suffer from the presence of spurious oscillations. However, the latter are not limited to this kind of molecules and could be identified in all sorts of molecular systems. In section 4 of the Supporting Information, we show that molecules like H_2_O_2_, H_2_S_2_, the allyl anion, butadiene, cyclobutadiene, benzene, naphthalene, and phenanthrene also exhibit spurious oscillations for various energy and dipole derivatives over certain normal modes. All the evidence indicates that the oscillations are a universal feature of modern DFAs that can affect molecules with low-frequency vibrational modes. Given the ubiquitous nature of the latter, the grid-dependency problem of modern DFAs constitutes a challenge to the development of new approximations that calls for the inclusion of molecular properties and the study of spurious oscillations in the construction of new DFAs.

## Supporting Information Available

Field-induced coordinates; technical details of the algorithm to detect and quantify spurious oscillations; procedure to classify the DFAs in rungs; computational details (including a description of the numerical differentiation procedure); figures with the data of Figure 3 extended to all DFAs and all the properties; maximum order of derivatives that can be safely calculated with (99,590) and (750,974) grids: averages and individual assessments per molecule; relative errors of various vibrational molecular properties compared against RRMSE values; and section 4, various regular covalent systems that also exhibit spurious oscillations (PDF)
Spreadsheet of the raw data (XLSX)
