# Supplementary material for: How Reliable Are Modern Density Functional Approximations to Simulate Vibrational Spectroscopies?
Source: J Phys Chem Lett. 2022 Jun 23;13(25):5963–8. doi: 10.1021/acs.jpclett.2c01278 (PMC9251762; doi:10.1021/acs.jpclett.2c01278)
Supplement: Supplementary file 1 — jz2c01278_si_001.pdf [file jz2c01278_si_001.pdf]

Supplementary Information: How Reliable are Modern Density Functional Approximations to Simulate Vibrational Spectroscopies?

Sebastian P. Sitkiewicz,[†,‡] Robert Zaleśny,[¶] Eloy Ramos-Cordoba,[†,‡] Josep M. Luis,[*,§] and Eduard Matito[*,†,‖]

†*Donostia International Physics Center (DIPC), 20018 Donostia, Euskadi, Spain*

‡*Polimero eta Material Aurreratuak: Fisika, Kimika eta Teknologia, Kimika Fakultatea, Euskal Herriko Unibertsitatea UPV/EHU, P.K. 1072, 20080 Donostia, Euskadi, Spain.*

¶*Faculty of Chemistry, Wrocław University of Science and Technology, Wyb. Wyspiańskiego 27, PL–50370 Wrocław, Poland*

§*Institut de Química Computacional i Catàlisi (IQCC) and Departament de Química, Universitat de Girona, 17003 Girona, Catalonia, Spain*

‖*Ikerbasque Foundation for Science, Plaza Euskadi 5, 48009 Bilbao, Euskadi, Spain*

E-mail: josepm.luis@udg.edu; ematito@gmail.com

Contents

S1 Methodology **3**

S1.1 Molecular Property Curves. Field Induced Coordinate 3

S1.2 Procedure to Detect and Quantify Spurious Oscillations 5

S1.3 Five-Rung Classification of the DFAs 13

S2 Computational Details **15**

S2.1 Electronic Structure Calculations 15

S2.2 Numerical Differentiation 17

S3 Raw data **22**

S4 Spurious Oscillations in Low-Frequency Modes of Other Molecules **44**

S4.1 Allyl anion (Q_2 mode) 44

S4.2 Butadiene (Q_2 and Q_2 modes) 47

S4.3 Cyclobutadiene (Q_4 mode) 49

S4.4 Benzene (Q_4 mode) 51

S4.5 Naphthalene (Q_3 mode) 53

S4.6 Phenanthrene (Q_4 mode) 56

S4.7 H_2O_2 (Q_1 mode) 58

S4.8 H_2S_2 (Q_1 mode) 63

References **68**

S1 Methodology

S1.1 Molecular Property Curves. Field Induced Coordinate

To effectively describe the most anharmonic nuclear displacements in polyatomic systems, we adopted the first–order Field Induced Coordinate (FIC), $\chi_{1,i}$.[1] It is defined as

$$\chi_{1,i} = -\sum_{\nu=1}^{3N-6} Q_\nu q_1^{\nu,i} \quad \text{and} \quad q_1^{\nu,i} = \frac{1}{2k_\nu}\left(\frac{\mathrm{d}\mu_i}{\mathrm{d}Q_\nu}\right)_{\boldsymbol{Q}=0,\boldsymbol{F}=0}, \tag{1}$$

where Q_ν is the ν–th normal mode with a corresponding force constant k_ν, and μ_i is the i–th component of the static dipole moment. All those properties are obtained at the optimized field–free geometry for a given computational method. From its definition, it is obvious that floppy vibrational modes (characterized by very low k_ν) have a substantial contribution to $\chi_{1,i}$.

In the case of hydrogen- and halogen-bonded systems, the property curves were obtained for linear displacements along the first-order FIC vector, $\chi_{1,z}$. For these systems, $\chi_{1,z}$ is parallel to the main intermolecular axis and involves only vibrational modes of the σ-type. In fact, $\chi_{1,z}$ almost exclusively consists of the low-frequency intermolecular stretching mode between two molecules held by a noncovalent interaction (see Figure S1).

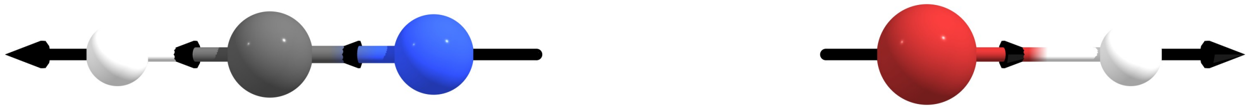

Figure S1: Graphical representation of $\chi_{1,z}$ (in normalized Cartesian coordinates) of HCN·BrH obtained with B3LYP/aug-cc-pVTZ and the (250,974) integration grid. For this system, according to eq 1, $\chi_{1,z} = 0.62Q_1 + 0.19Q_2 + 0.21Q_3 - 219.48Q_4$, where Q_1-Q_4 are σ-type normal modes with the harmonic frequencies listed in Table S19.

Therefore, one can try to roughly express $\chi_{1,z}$ in terms of the separation between two molecules, where $\pm\Delta\chi_{1,z}$ corresponds roughly to $\pm$0.15Å in the intermolecular separation. However, for the sake of generality, in this paper, we have preferred to use FICs which would be the most efficient way of studying the spurious oscillations of more complex polyatomic systems. In this work, for polyatomic systems, curves were sampled with 400 points and $\Delta(\Delta\chi_{1,z})$ = 0.02 au, with the opti-

mized unperturbed geometry corresponding to $\Delta\chi_{1,z} = 0$.

In the case of dispersion-bonded systems, scan along the interatomic distance R has been done. The curves were sampled with $\Delta R = 0.005$ a.u. and consisted of 1000 points. In all cases, irrespective if the methods give a bounded system or not, starting points $R = 7.2$ a.u. and $R = 6.0$ a.u. were taken for Ar_2 and He_2, respectively.

Afterward, for such generated property curves their derivatives were calculated numerically (see Section S2.2).

S1.2 Procedure to Detect and Quantify Spurious Oscillations

In this section, the algorithm for the detection and quantification of spurious oscillations along a nuclear displacement is discussed in detail.

We refer to the property profile, which is the property (for instance, an energy derivative) along a nuclear displacement coordinate. For convenience, we have chosen the FICs as the nuclear coordinate (see the previous section). The number of points that will be analyzed on the property profile defines the set of geometries indicated as $\{\boldsymbol{\xi_0}\}$, and we will refer to them as *displacement range*.

The algorithm will need two inputs: the property profile we want to analyze and will be calculated with a certain DFA, which we will refer to as P^{DFA}, and some spurious-oscillation free reference, P^{ref}. In order to guarantee that the latter does not have spurious oscillations it is convenient to choose a computational method that does not rely on numerical integration, such as Hartree-Fock (HF), MP2 or coupled-cluster. The algorithm will provide a quantitative measure of the degree of oscillations that are present in the property profile within the displacement range.

The algorithm consists of two stages (see Figure S2): 1) obtaining an oscillation-free version of P^{DFA}, $P^{\mathrm{DFA}}_{\mathrm{filt}}$, which can be directly compared to P^{DFA}, and 2) quantifying the spurious oscillations by comparing $P^{\mathrm{DFA}}_{\mathrm{filt}}$ and P^{DFA}. There are two variants of the algorithm, which will be commented below. The algorithm has been implemented in an in-house Python3 program, which uses NumPy,[2] SciPy,[3] and SymPy[4] libraries, and it is available from the authors upon request.

In the first stage, the algorithm detects and filters out the eventual spurious oscillations in P^{DFA}. Since the main goal of this stage is obtaining $P^{\mathrm{DFA}}_{\mathrm{filt}}$, we do not have to employ the grid that we actually want to test. In fact, it is convenient to use the same DFA we want to test with a larger integration grid (g1), which will have much less spurious oscillations than a lower-quality grid

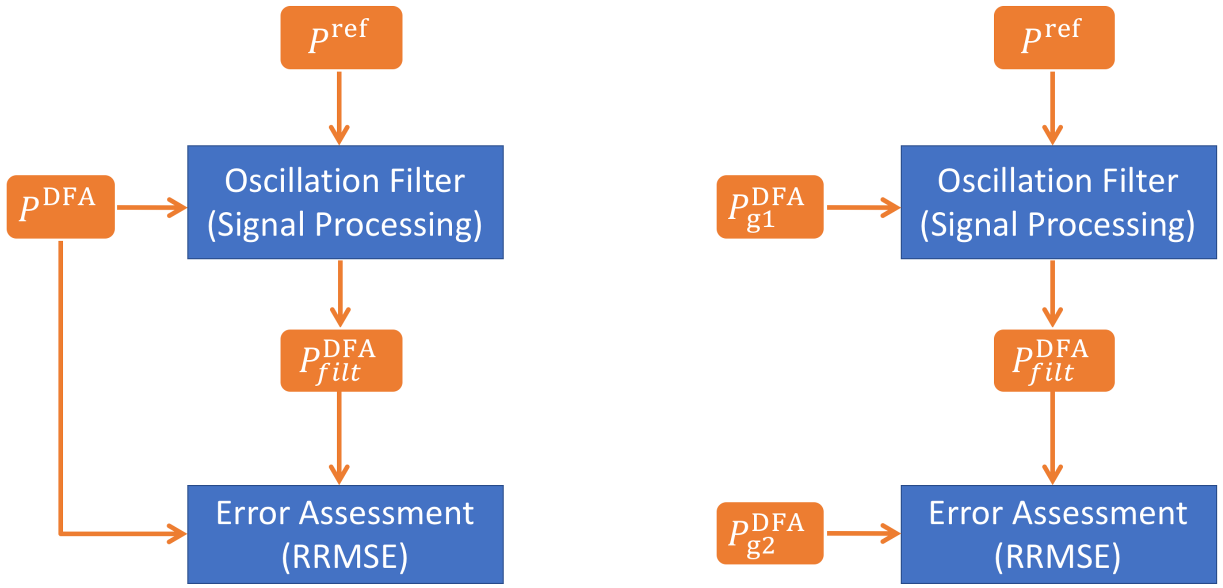

Figure S2: *Left:* **Algorithm 1**, which uses the property profile to be tested also to obtain the filtered profile. *Right:* **Algorithm 2**, which uses a larger-grid (g1) to obtain the filtered profile that will be used to quantify the spurious oscillations on a smaller grid (g2). In all cases, the same DFA and basis are employed.

(g2) and, therefore, it will be more suitable to remove the spurious oscillations (**Algorithm 2** in Figure S2). However, as we will comment later, the algorithm can be easily adapted to confidently filter the oscillations of any grid size. **Algorithm 1** in Figure S2 uses the same grid of the tested DFA calculation to generate $P^{\text{DFA}}_{\text{filt}}$. In this first work, to eliminate the uncertainty about the presence of spurious oscillations in the DFAs we study, and we have decided to employ the (750,974) grid for this first stage. Hence, $P^{\text{DFA}(750,974)}$ will be the input of this first stage of **Algorithm 2**. In this work, we have used reference property curves obtained with CCSD(T), $P^{\text{CCSD(T)}}$. However, the choice of the *ab-initio* method used to distinguish the (eventual) oscillations in $P^{\text{DFA}(750,974)}$ does not impact the results. Despite the evident differences in performance between HF, MP2, and CCSD(T) all these methods work equally well as spurious-oscillation-free references for this algorithm.

At the first stage, the discrete Fourier transforms of P^{DFA} and P^{ref} are compared. The comparison of their frequency spectra will reveal bands that are present on P^{DFA} but do not show in P^{ref} and will be marked as potentially spurious oscillatory bands. Figure S3 illustrates the property profiles of $P^{\mathrm{DFA}(750,974)}$ and $P^{\mathrm{CCSD(T)}}$ and their Fourier transform for HCN · BrH along $\Delta\chi_{1,z}$. The bands of both profiles are evenly matched by the means of an overlap criterion larger than 0.90. Any extra bands present in the spectrum of P^{DFA}, but not in P^{ref}, are labeled as spurious oscillatory bands (spurious bands, hereafter). For a better distinction of the spurious bands, which may have much lower amplitude than the main low-frequency band, the Kaiser windowing technique[5] is adapted to P^{DFA} and P^{ref} (with a different relative side-lobe attenuation for each type of derivative).

The spurious bands detected in P^{DFA} (along with high-frequency numerical noise) are filtered out, yielding a filtered reference curve, $P^{\mathrm{DFA}}_{\mathrm{filt}}$. This is done using a low-pass finite impulse response (FIR) filter of type I, designed with the Remez exchange algorithm.[6] Filter specifications (frequency cutoff, stop– and pass–band attenuation) are automatically chosen by our program. To this end, the position and height of the oscillation bands are used:

- The cutoff frequency corresponds to the right-most side of the main non-spurious band, which has the same height as the top of the detected oscillatory band.
- The stop-band attenuation is chosen to be 60dB smaller than the maximum of the highest oscillatory band.
- The pass-band attenuation is chosen to be 40dB.
- The number of filter coefficients (*i.e.*, the number of filter taps) is set to the first odd number smaller than the total number of points minus the number of points in the displacement range, $\{\xi_0\}$.
- The width of the transition is estimated using Bellanger's estimate.[7]

The latter parameters permit to filter the oscillations within $\{\xi_0\}$ with a very good precision and in an automatized manner. Such designed FIR filter will provide a valid part of the filtered profile

only within $\{\boldsymbol{\xi}_0\}$; the resulting profile outside the displacement range could be corrupted by the initial conditions of the FIR filter.

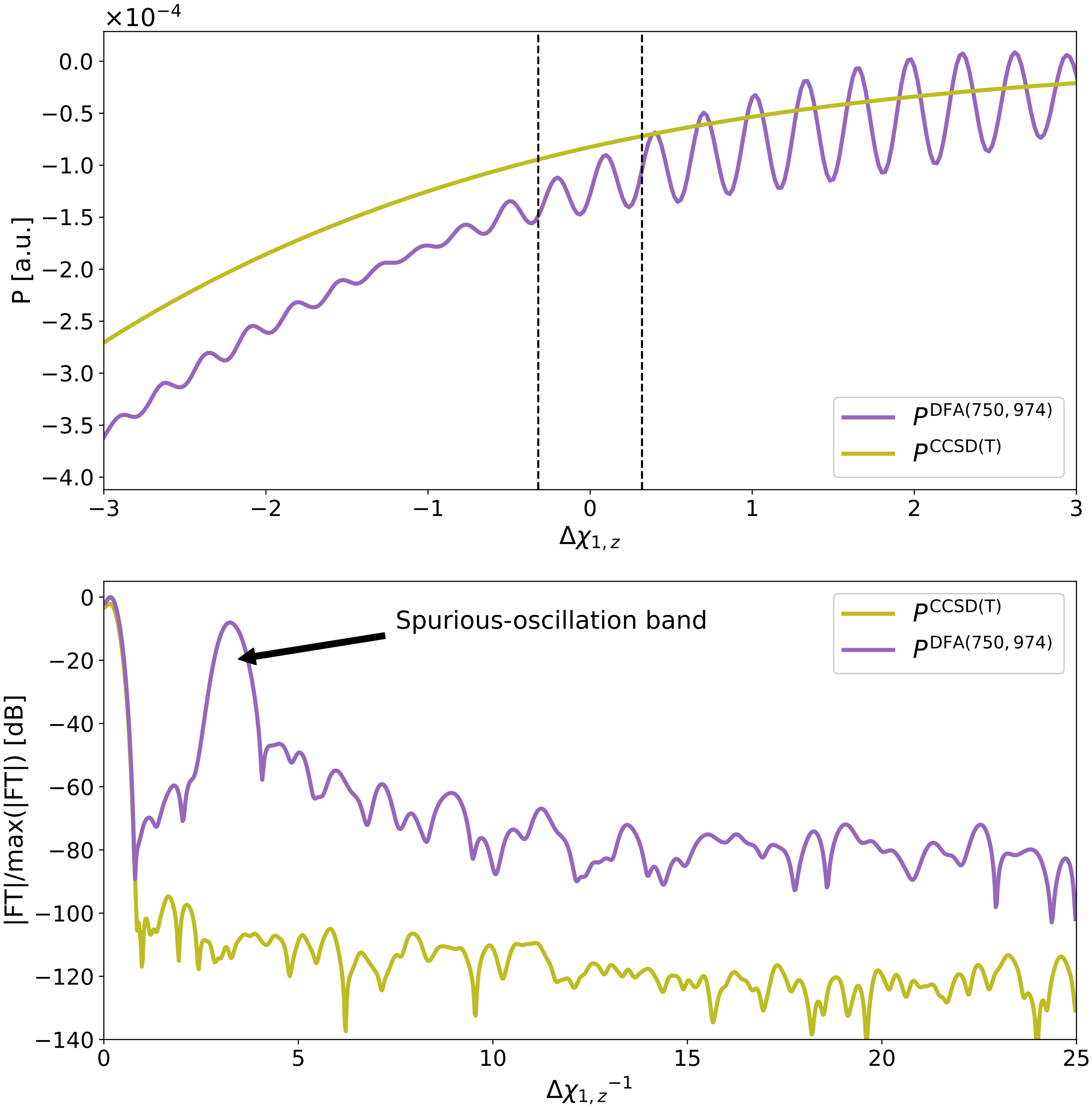

Figure S3: *Top:* $\mathrm{d}^3E/\mathrm{d}\chi_{1,z}{}^3$ curves for HCN $\cdot$ BrH along the $\Delta\chi_{1,z}$ displacement, obtained with the ωB97X/aug-cc-pVTZ combined with the reference (750,974) grid, $P^{\mathrm{DFA}(750,974)}$ (purple). Equivalent property curve obtained with CCSD(T), $P^{\mathrm{CCSD(T)}}$, is shown in yellow. *Bottom:* Frequency spectra of $P^{\mathrm{DFA}(750,974)}$ and $P^{\mathrm{CCSD(T)}}$. The band corresponding to the spurious oscillation present in $P^{\mathrm{DFA}(750,974)}$, but not in $P^{\mathrm{CCSD(T)}}$, is indicated with a black arrow.

An example of the FIR filter and the result of filtering are shown in Figure S4. The separation of the spurious band from the main low-frequency band (which determines the general shape of the curve) is the key factor to a successful filtering. We have tested that the main low-frequency band is well separated from the spurious bands when the (750,974) grid is used in the first stage. Using $P^{\mathrm{DFA}(250,974)}$ in the first stage, in most cases, also yields the correct filtered profile $P_{\mathrm{filt}}^{\mathrm{DFA}(250,974)}$. However, difficulties arise for (99,590) or smaller grids because, in these cases, the spurious oscillations are very strong, span already at low frequencies, and cannot be easily filtered out using the same FIR filter. For smaller grids, one should adapt the FIR filter. Therefore, with the present FIR filter we recommend the use of large grids.

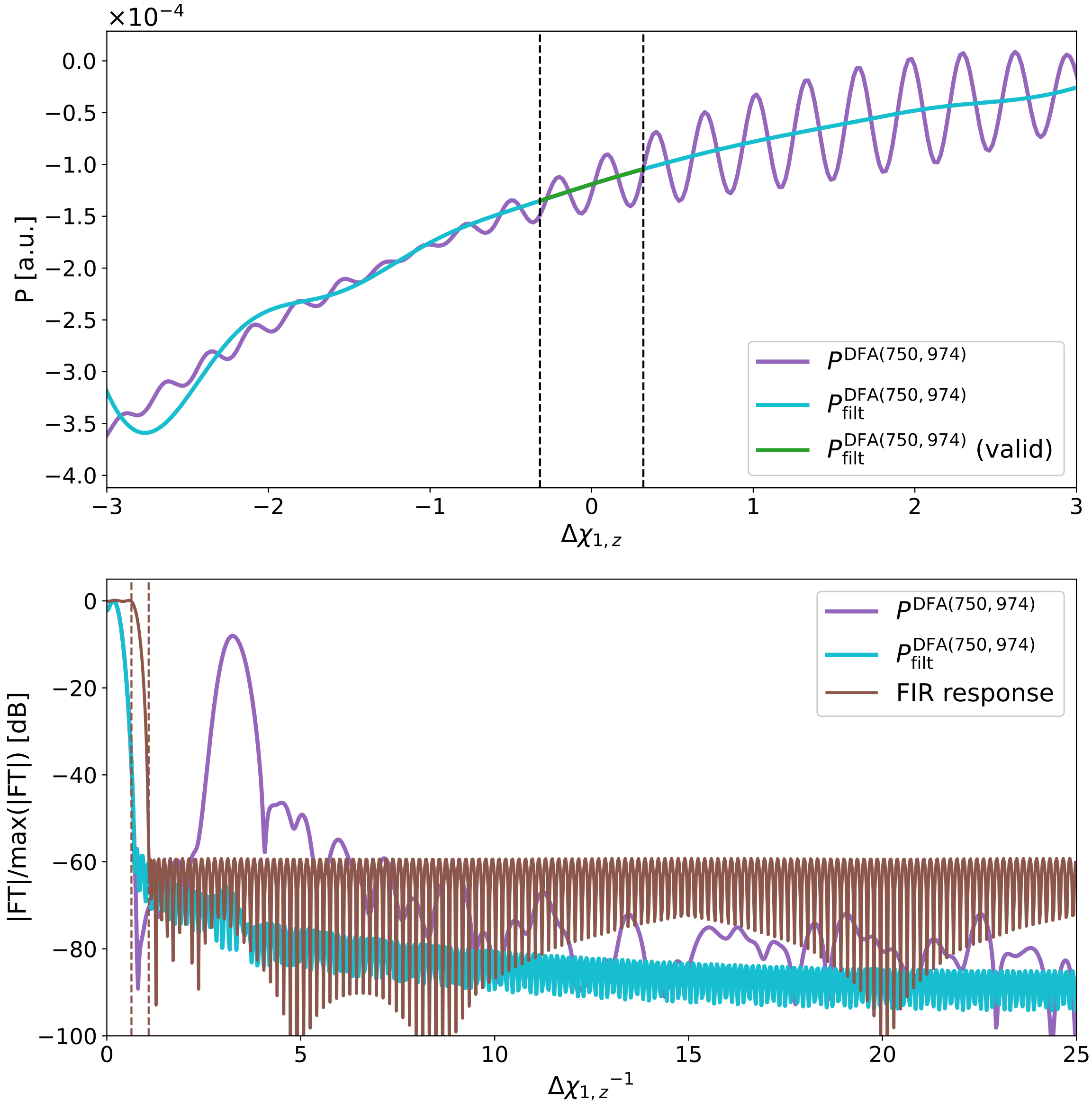

Figure S4: The procedure of filtering the reference curve $P^{\mathrm{DFA}(750,974)}$ with an automatically designed FIR filter, shown for $P = \mathrm{d}^3E/\mathrm{d}{\chi_{1,z}}^3$ of HCN · BrH using ωB97X/aug-cc-pVTZ. *Top:* Original and filtered curves in the $\chi_{1,z}$ domain, the valid part of the filtered curve is shown with green color. *Bottom:* Frequency spectra of the original and filtered curve (in reciprocal domain), along with the response of the designed FIR filter (shown in normalised decibel scale). In the top plot, dashed black vertical lines mark the studied range of the property curve $\{\boldsymbol{\xi}_\mathbf{0}\}$, for which RMS, RMSE, and RRMSE are calculated. In the bottom plot, dashed brown vertical lines mark the transition band of the designed low-pass filter.

At the second stage of the algorithm, we use $P^{\mathrm{DFA}}_{\mathrm{filt}}$ and P^{DFA} to quantify the spurious grid-

oscillations in the property curve obtained from some DFA and integration grid, P^{DFA}. At this stage, the size of the grid does not influence the process and we can test very small grids. We employ the root mean square (RMS) and the root mean square error (RMSE) to define the relative root mean square error (RRMSE), which quantifies the distortion of the property curve on the displacement range due to spurious oscillations.

$$RMSE = RMS\left[P^{\mathrm{DFA}} - P^{\mathrm{DFA}}_{\mathrm{filt}}\right], \tag{2}$$

and

$$RRMSE = \frac{RMSE}{RMS\left[P^{\mathrm{DFA}}_{\mathrm{filt}}\right]} \cdot 100. \tag{3}$$

In the current analysis, the RMS involves the geometries within the displacement range, which we have selected following these criteria:

- For hydrogen– and halogen– bonded systems: 33 points in the range $0 \pm 0.32\Delta\chi_{1,z}$ (*i.e.*, displacements along the normalized FIC coordinate, starting from the optimized geometry).
- For dispersion-bonded systems: 129 points in the range 7.2 ± 0.32 a.u. for Ar_2, and 6.0 ± 0.32 a.u. for He_2 (*i.e.*, displacements along the interatomic separation close to the overall minimum in the potential energy of Ar_2 and He_2).

RMS, RMSE, and RRMSE with reversed sign are included in the plots of Figure S5. In this example, the curve obtained with ωB97X and the reference (750,974) grid for HCN$\cdot$BrH, $P^{\mathrm{DFA}(750,974)}$ (purple color in Figure S5) still suffers from spurious oscillations. The spurious oscillations are qualitatively filtered out in $P^{\mathrm{DFA}(750,974)}_{\mathrm{filt}}$ (green color in Figure S5). Curves obtained with other grids ($P^{\mathrm{DFA}(99,590)}$, $P^{\mathrm{DFA}(250,974)}$, and $P^{\mathrm{DFA}(750,974)}$) are also shown for comparison. The difference between the latter curves and $P^{\mathrm{DFA}(750,974)}_{\mathrm{filt}}$ yields the red curves in Figure S5, which represent the spurious oscillations in $P^{\mathrm{DFA}(750,974)}$ within the ξ_0 range.

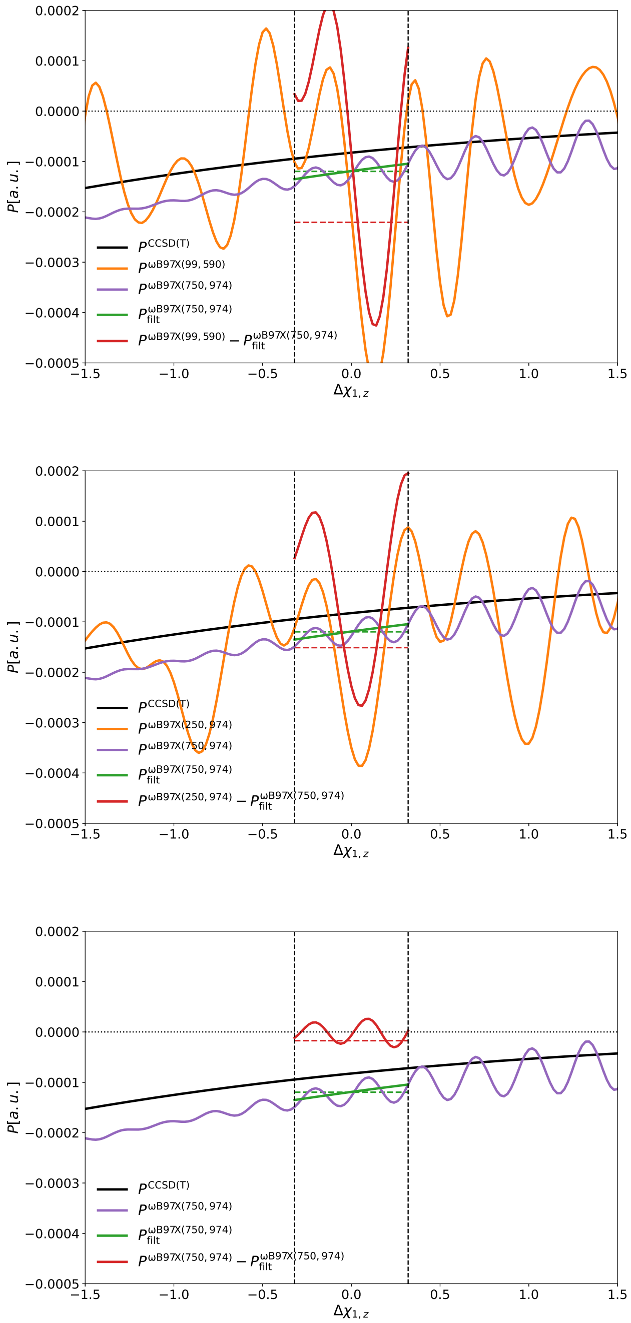

Figure S5: The P_{g1}^{DFA}, P_{g2}^{DFA} and $P_{\mathrm{filt}}^{\mathrm{DFA}}$ curves of $\mathrm{d}^3E/\mathrm{d}\chi_{1,z}{}^3$ of the HCN · BrH system. Calculated with ωB97X/aug-cc-pVTZ and three tested grids g2: (99,590) *(top)*, with RMSE = $2.21 \cdot 10^{-4}$ and RRMSE = 184.7%; (250,974) *(middle)*, with RMSE = $1.51 \cdot 10^{-4}$ and RRMSE = 126.3%; (750,974) *(bottom)*, with RMSE = $1.72 \cdot 10^{-5}$ and RRMSE = 14.4%. In all computations, g1 = (750,974) was utilized. Dashed colored horizontal limes mark the negative value of RMSE (red) and RMS of the filtered profile (green). Dashed black vertical lines mark the studied range of the property curve $\{\boldsymbol{\xi_0}\}$, for which RMS, RMSE, and RRMSE are calculated.

S1.3 Five-Rung Classification of the DFAs

In Figure 4 of the manuscript, DFAs were ordered with respect to their overall performance in obtaining the highest derivatives of E, μ_z, and α_{zz}, when combined with the (250,974) grid. DFAs were sorted according to the weights defined as

$$\omega^{\mathrm{DFA}} = \frac{1}{3}\left(\frac{1}{12}\sum_{G}^{\mathrm{A,B,C}}\max_{i\in G}(n_{E_i}) + \frac{1}{9}\sum_{G}^{\mathrm{A,B}}\max_{i\in G}(n_{\mu_{z,i}}) + \frac{1}{6}\sum_{G}^{\mathrm{A,B,C}}\max_{i\in G}(n_{\alpha_{zz,i}})\right), \quad (4)$$

where n_{E_i}, $n_{\mu_{z,i}}$ and $n_{\alpha_{zz,i}}$ are, respectively, the lowest maximum order of the derivative that can be safely calculated with that DFA (RRMSE$\leq$ 10%) for the energy, the dipole moment, and the polarizability of molecule i. If the weight is one for a given DFA, it means that such DFA can safely compute all the high-order derivatives of E, μ_z, and α_{zz}. DFAs along with their weights are presented in Table S1.

Table S1: Lowest maximum order of derivatives that can be safely obtained with the (250,974) grid (*i.e.*, for which the RRMSE was lower than 10%), found for each group of complexes: A – hydrogen-, B – halogen-, and C – dispersion-bonded. Last column presents the weight of the overall performance, defined in eq 4 of the Supporting Information.

	E			μ_z		α_{zz}			
	A	B	C	A	B	A	B	C	weight
BH&H	4	4	3	3	3	2	2	2	0.972
LC-BLYP	4	4	3	3	3	2	2	2	0.972
PBE	4	4	2	3	3	2	2	1	0.889
CAM-B3LYP	4	4	2	3	3	2	2	1	0.889
BH&HLYP	4	3	2	3	3	2	2	1	0.861
HSE06	4	4	1	3	3	2	2	0	0.806
PBE0	4	4	1	3	3	1	2	1	0.806
LC-ωHPBE	4	4	2	3	3	1	2	0	0.778
M11-L	3	3	2	3	3	2	2	0	0.778
B3LYP	4	3	1	3	3	2	2	0	0.778
RevTPSS	4	3	1	3	3	2	2	0	0.778
M11	3	3	1	3	3	2	2	0	0.750
MN15	4	4	1	3	3	1	2	0	0.750
B2PLYP	4	4	1	2	2	2	2	1	0.750
BLYP	4	3	2	3	2	2	2	0	0.750
HSE03	3	2	1	3	3	2	2	0	0.722
MN12-L	4	3	1	3	3	1	2	0	0.722
PBE-QIDH	4	4	1	2	3	1	2	0	0.694
MN12-SX	3	3	1	3	3	1	2	0	0.694
TPSSh	4	3	1	3	2	2	1	0	0.667
TPSS	4	3	1	3	2	2	1	0	0.667
B1LYP	4	3	1	3	2	1	2	0	0.667
SOGGA11-X	4	3	1	3	2	2	1	0	0.667

	E			μ_z		α_{zz}			
	A	B	C	A	B	A	B	C	weight
ωB97X-V	4	3	1	3	1	-	-	-	0.667
ωB97M-V	3	3	0	3	2	-	-	-	0.667
LC-ωPBE	2	2	1	3	3	1	2	0	0.639
B97M-V	3	3	1	2	2	-	-	-	0.625
mPW2PLYP	3	3	2	2	2	1	1	1	0.611
MN15-L	4	4	0	2	2	1	1	0	0.556
PBE-0DH	4	3	1	2	2	1	1	0	0.556
N12-SX	3	2	1	2	1	1	1	0	0.444
M06-2X	3	2	1	2	1	1	1	0	0.444
B97-D	2	2	1	2	1	1	1	0	0.417
M06	3	2	0	2	1	1	1	0	0.417
ωB97	2	2	1	2	1	1	0	0	0.361
ωB97X	2	2	1	2	1	1	0	0	0.361
SOGGA11	2	2	2	1	1	0	1	0	0.333
B97	3	0	1	2	1	1	0	0	0.333
SCAN	2	2	1	1	1	0	1	0	0.306
SCAN0	2	2	1	1	1	0	1	0	0.306
ωB97X-D3	2	2	1	1	1	1	0	0	0.306
M06-L	3	0	0	2	1	1	0	0	0.306
M06-HF	2	0	0	2	1	1	0	0	0.278
N12	2	0	1	1	1	1	0	0	0.250
ωB97XD	2	0	1	1	1	1	0	0	0.250

S2 Computational Details

S2.1 Electronic Structure Calculations

For all DFAs (see Table S2), KS-DFT computations were performed using Gaussian16[8], except for B97, B97-D, SCAN, SCAN0, ωB97X-D3, B97M-V, ωB97X-V, ωB97M-V, which were performed using QChem 5.1.[9] CCSD(T) computations were done using CFOUR.[10] In the manuscript and Sections 1 to 3 of this Supporting Information, all of the computations employed the aug-cc-pVTZ[11–18] basis set, whereas Section 4 of this Supporting Information employed the aug-cc-pVDZ basis set.

Table S2: List of DFAs used thorough this work; GGA - Generalized Gradient Approximation, GH - global hybrids, RSH - range-separated hybrids, DH - double hybrids.

DFA	Type	Ref.	DFA	Type	Ref.
BLYP	GGA	19,20	PBE	GGA	21
B1LYP	GH GGA	22	PBE0	GH GGA	23
B3LYP	GH GGA	24,25	LR-wPBE	RSH GGA	26
BH&H	GH GGA[a]	8	LR-wHPBE	RSH GGA	8
BH&HLYP	GH GGA	8	B97	GH GGA	27
LC-BLYP	RSH GGA	28	B97-D	GGA	29
CAM-B3LYP	RSH GGA	30	ωB97	RSH GGA	31
M06	GH meta-GGA	32	ωB97X	RSH GGA	31
M06-L	meta-GGA	33	ωB97X-D	RSH GGA	34
M06-HF	GH meta-GGA	35	ωB97X-D3	RSH GGA	36
M06-2X	GH meta-GGA	32	B97M-V	meta-GGA	37
SOGGA11	GGA	38	ωB97M-V	RSH meta-GGA	39
SOGGA11-X	GH GGA	40	ωB97X-V	RSH GGA	41
M11	RSH meta-GGA	42	TPSS	meta-GGA	43
M11-L	meta-GGA	44	RevTPSS	meta-GGA	45
MN12-L	meta-GGA	46	TPSSh	GH meta-GGA	47
N12-SX	RSH GGA	48	PBE-0DH	DH GGA	49
N12	GGA	42	PBE-QIDH	DH GGA	50
MN12-SX	RSH meta-GGA	48	B2PLYP	DH GGA	51
MN15	GH meta-GGA	52	mPW2PLYP	DH GGA	53
MN15-L	meta-GGA	54	HSE03	RSH GGA	55,56
SCAN	meta-GGA	57	HSE06	RSH GGA	58
SCAN0	GH meta-GGA	59			

[a] No GGA exchange is used, *i.e.*, $E_{xc}^{BHH} = 0.5\, E_x^{Slater} + 0.5\, E_x^{HF} + E_c^{LYP}$

The SCF DIIS converge criteria was set to 10^{-12} in the HF and KS–DFT computations, whereas in CCSD(T) the convergence threshold for maximum amplitude was set to 10^{-10}. In the coupled–perturbed equations, convergence threshold was set to 10^{-11}. In all methods, the screening threshold for the two–electron integrals was set to 10^{-14}. Except Ar_2 and He_2, all molecular systems were optimized at the corresponding level of theory with the convergence criteria of RMS of the gradient set below 10^{-6} (and 10^{-8} in case of the CCSD(T)). In the optimization procedure with DFAs, only the (250,974) grid has been applied for the integration.

Molecular properties E, μ_z, and α_{zz} have been obtained analytically. In the case of double hybrid functionals and CCSD(T), the relaxed densities have been used to compute μ_z and α_{zz}.

Table S3: The largest predefined grids in some quantum chemical computational packages. The fourth column lists the parent unpruned grids (shown for C atom).

Program	Grid	Radial Quadrature	Parent Grid Size
Gaussian16	SuperFineGrid	Handy	(250,974)
QCHEM 5.3	SG3	DE[60–63]	(99,590)
ORCA 5.0.1	DEFGRID3	Ahlrichs[64]	(45,590)
GAMESS US	JANS	Handy[65]	(150,947)
NWChem 7.0.2	huge	Knowles[66]	(300,1454)
DALTON	UltraF	LMG[67]	(111,1454)

In the KS-DFT computations, we have used Euler–Maclaurin–Lebedev grids, (N_r, N_Ω), where N_r is number of radial shells and N_Ω is number of points in the Lebedev angular grid. In this study, the following unpruned integration grids were tested: (99,590), (250,974), (500,974) and (750,974), along with some of their pruned versions: UltraFine, SG3, and SuperFineGrid. For the integration of nonlocal VV10[68] correlation in B97M-V, ωB97X-V and ωB97M-V, the SG3 grid has been used. The smallest unpruned grid tested in our study, (99,590), is an unpruned version of UltraFine[8] (a default predefined grid in Gaussian16) and SG3[69] (the largest predefined grid in QChem5.1[9]). The (250,974) grid is the unpruned version of a popular (and the largest) predefined grid in Gaussian16, known as the SuperFineGrid. Basic information on the largest predefined grids in some quantum chemical packages is compiled in Table S3.

Table S4: Molecular complexes included in the benchmark and the type of intermolecular interaction responsible for their binding.

Complex	Intermolecular interaction
$HCN \cdot HF$	hydrogen-bond
$HCN \cdot HCl$	hydrogen-bond
$N_2 \cdot HF$	hydrogen-bond
$OC \cdot HF$	hydrogen-bond
$HCN \cdot BrH$	halogen-bond
$HCN \cdot BrF$	halogen-bond
He_2	dispersion
Ar_2	dispersion

S2.2 Numerical Differentiation

Derivatives $\mathrm{d}^m E/\mathrm{d}\xi^m$ ($m = 1-4$), $\mathrm{d}^m \mu_z/\mathrm{d}\xi^m$ ($m = 1-3$) and $\mathrm{d}^m \alpha_{zz}/\mathrm{d}\xi^m$ ($m = 1-2$) have been obtained through numerical differentiation. For that purpose, we have constructed an algorithm to automatically choose the Savitzky-Golay[70] differentiation filter (*i.e.*, differentiation via polynomial fitting). We will refer to this method as AutoSG. The order of the filter (*i.e.*, the order of the interpolating polynomial) has been chosen automatically, with the criterion to quantitatively differentiate all relevant (*i.e.*, non-noisy) bands in the Fourier Transform spectrum of the property. In this procedure, a maximum order of 30 has been used as the limit. The length of the filter (*i.e.*, the number of points used in the fitting) has been set to 51, 75, and 101 for hydrogen-, halogen- and dispersion-bonded systems, respectively. Such number of points provided a good trade-off between the cost and steepness of the cutoff region.

The most important feature of this algorithm is that it differentiates hidden spurious oscillatory bands and keeps the noise contribution to the minimum. Such numerical derivatives have proven to be of very good quality and superior to ones obtained with a finite (central) differentiation or even the Romberg-Rutishauser procedure.[71] If one would use the finite (central) differentiation with a very large step size, one could miss the differentiation of high-frequency spurious oscillations. However, the latter does not represent a solution to the spurious oscillations because there is no guarantee that finite-difference numerical derivatives are not affected by the spurious oscillations. Besides, since spurious oscillations are an inherent problem of the DFA, analytical derivatives –

which are employed in many computational packages for low-order derivatives– would also present spurious oscillations on the property profile.

In Figures S6-S8, we show a performance comparison of numerical differentiation techniques, including our newly implemented AutoSG. In the test, the formula for central differentiation (utilizing Richardson extrapolation once, which we label here as $CD(n,1)$) has been used:

$$\begin{aligned} CD(n,1) = \frac{1}{48\Delta R^4}\,[&-E(R-4\Delta R)+68E(R-2\Delta R)-256E(R-\Delta R)+378E(R) \\ &-256E(R+\Delta R)+68E(R+2\Delta R)-E(R+4\Delta R)] \qquad (5) \\ &\text{where} \quad \Delta R = 2^n\cdot 0.005\,\text{a.u.} \end{aligned}$$

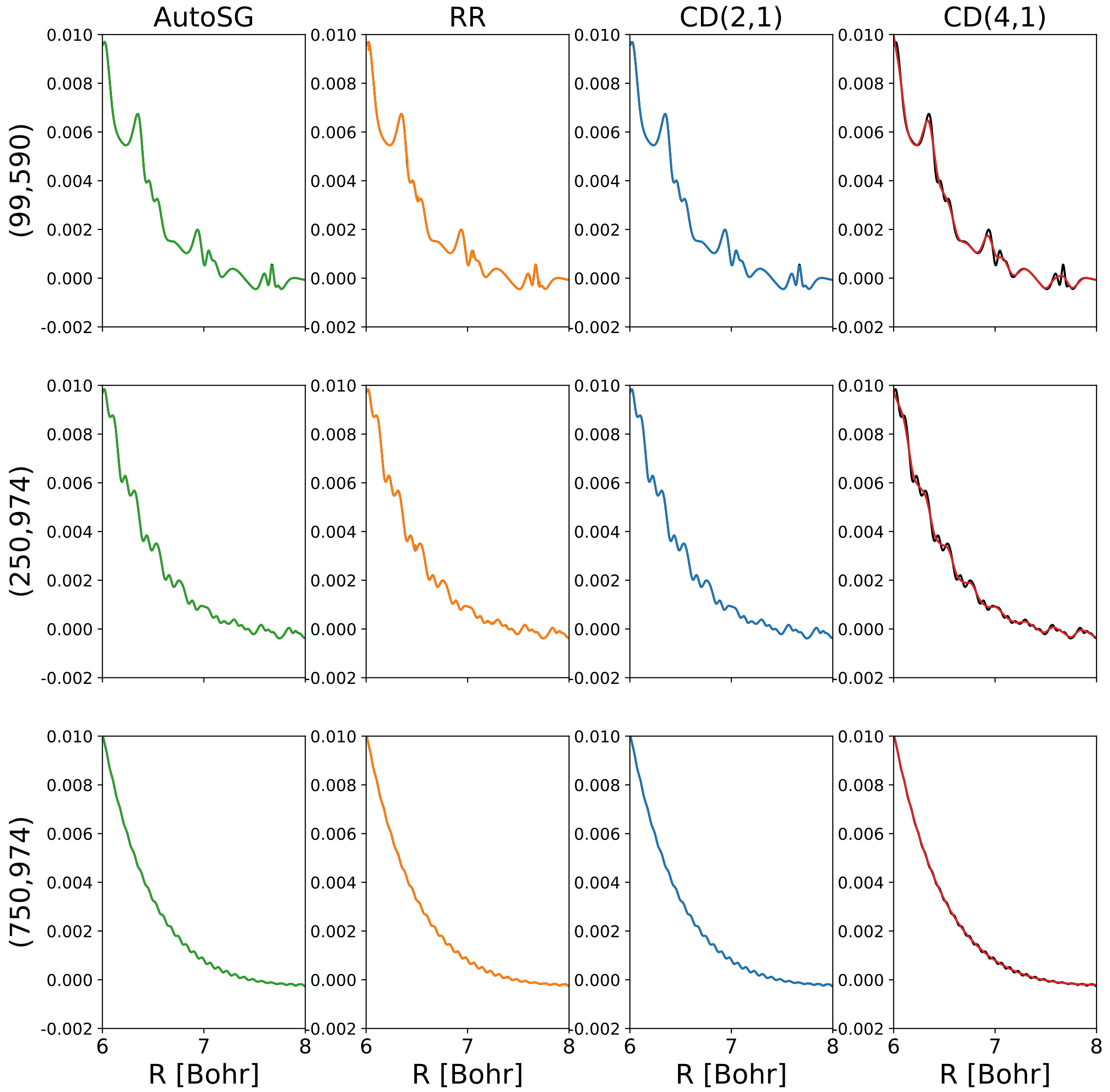

Figure S6: Accuracy of different numerical differentiation procedures to compute d^2E/dR^2 in Ar_2. Black curve, REF, represents the reference curve obtained as the analytically obtained d^2E/dR^2. Other curves were obtained as numerical second derivatives from the total energies. AutoSG is our automatic differentiation using Savitzky-Golay filtering; RR corresponds to Romberg-Rutishauser differentiation with $k_{\max} = 4$ and $\Delta R = 0.005$ a.u.; CD(2,1) corresponds to the central differentiation and uses $\Delta R = 0.02$ a.u. starting step; CD(4,1) corresponds to the central differentiation and uses $\Delta R = 0.08$ a.u. starting step; all curves were obtained using the ωB97X functional, combined with different integration grids, namely (99,590) - top row, (250,974) - middle row, and (750,974) - bottom row.

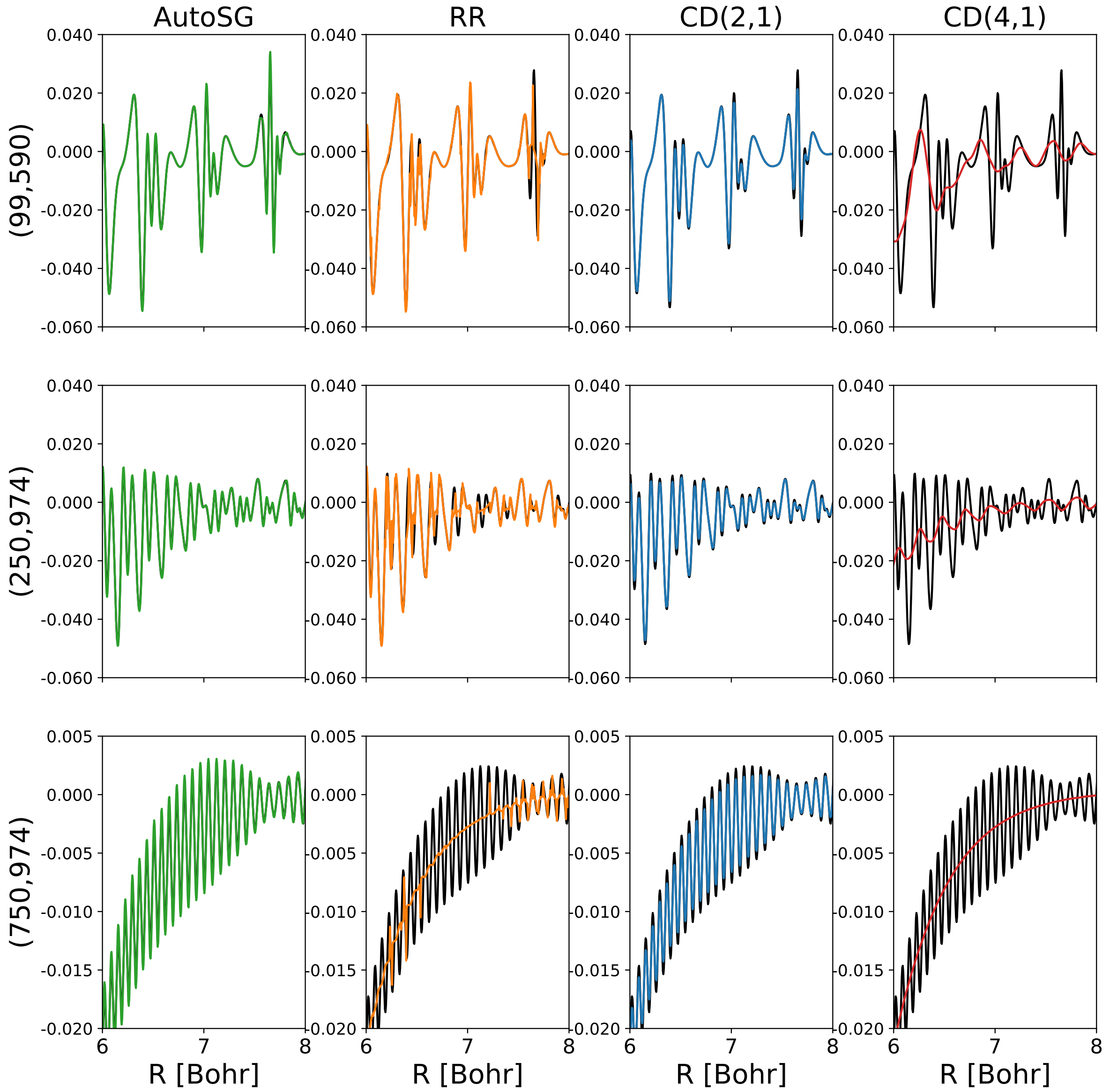

Figure S7: Accuracy of different numerical differentiation procedures to compute d^3E/dR^3 in Ar_2. Black curve, REF, represents the reference curve obtained as numerical first derivative (utilizing CD(2,1)) of the analytically obtained d^2E/dR^2. Other curves were obtained as numerical third derivatives from the total energies. AutoSG is our automatic differentiation using Savitzky-Golay filtering; RR corresponds to Romberg-Rutishauser differentiation with $k_{max} = 4$ and $\Delta R = 0.005$ a.u.; CD(2,1) corresponds to the central differentiation and uses $\Delta R = 0.02$ a.u. starting step; CD(4,1) corresponds to the central differentiation and uses $\Delta R = 0.08$ a.u. starting step; all curves were obtained using the ωB97X functional, combined with different integration grids, namely (99,590) - top row, (250,974) - middle row, and (750,974) - bottom row.

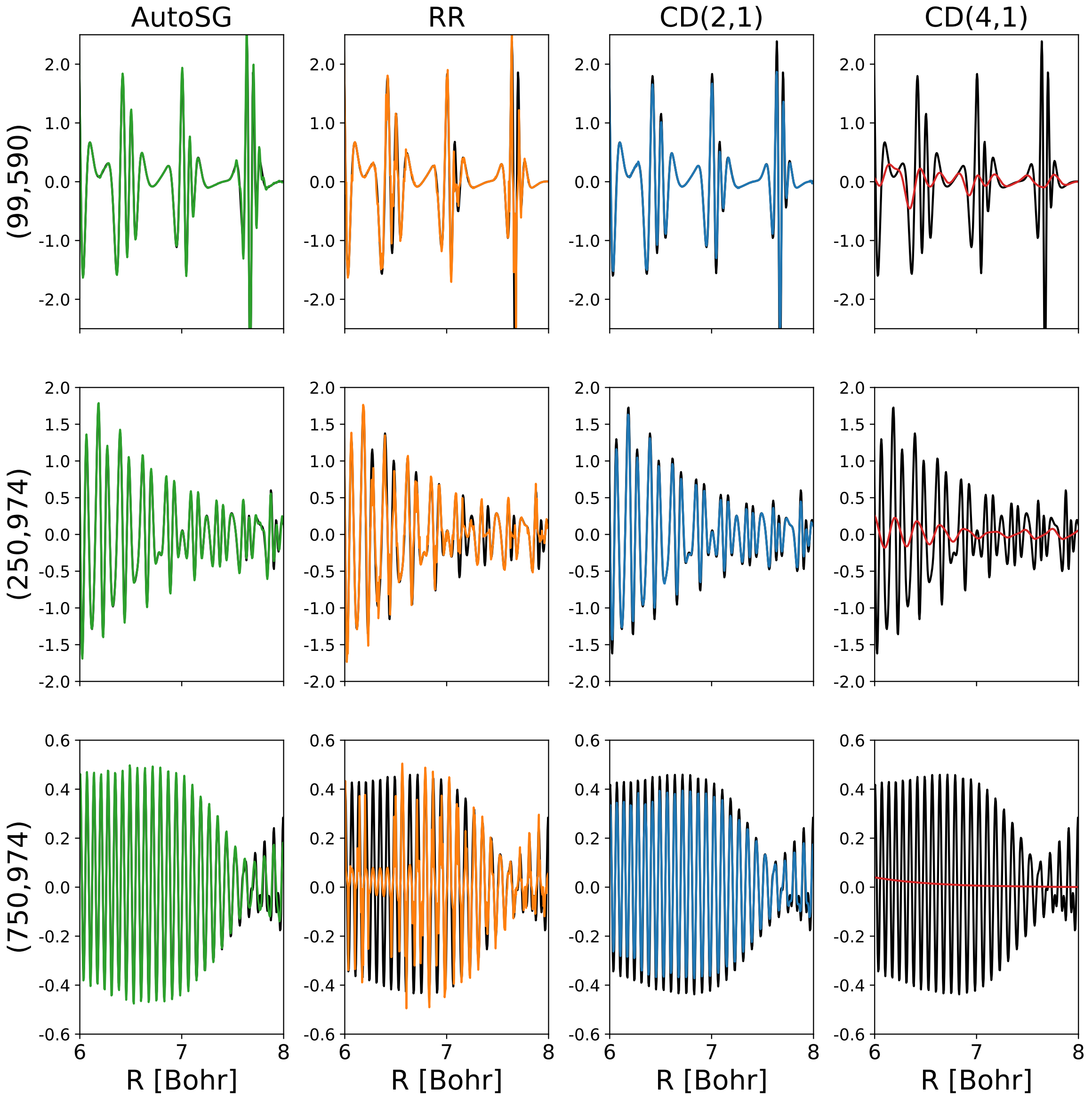

Figure S8: Accuracy of different numerical differentiation procedures to compute d^4E/dR^4 in Ar_2. Black curve, REF, represents the reference curve obtained as numerical second derivative (utilizing CD(2,1)) of the analytically obtained d^2E/dR^2. Other curves were obtained as numerical fourth derivatives from the total energies. AutoSG is our automatic differentiation using Savitzky-Golay filtering; RR corresponds to Romberg-Rutishauser differentiation with $k_{max} = 4$ and $\Delta R = 0.005$ a.u.; CD(2,1) corresponds to the central differentiation and uses $\Delta R = 0.02$ a.u. starting step; CD(4,1) corresponds to the central differentiation and uses $\Delta R = 0.08$ a.u. starting step; all curves were obtained using the ωB97X functional, combined with different integration grids, namely (99,590) - top row, (250,974) - middle row, and (750,974) - bottom row.

S3 Raw data

Raw RRMSE values for all studied derivatives, all studied DFAs, integration grids and for all molecular complexes, are compiled in an external spreadsheet file *raw_data_rrmse.xlsx*.

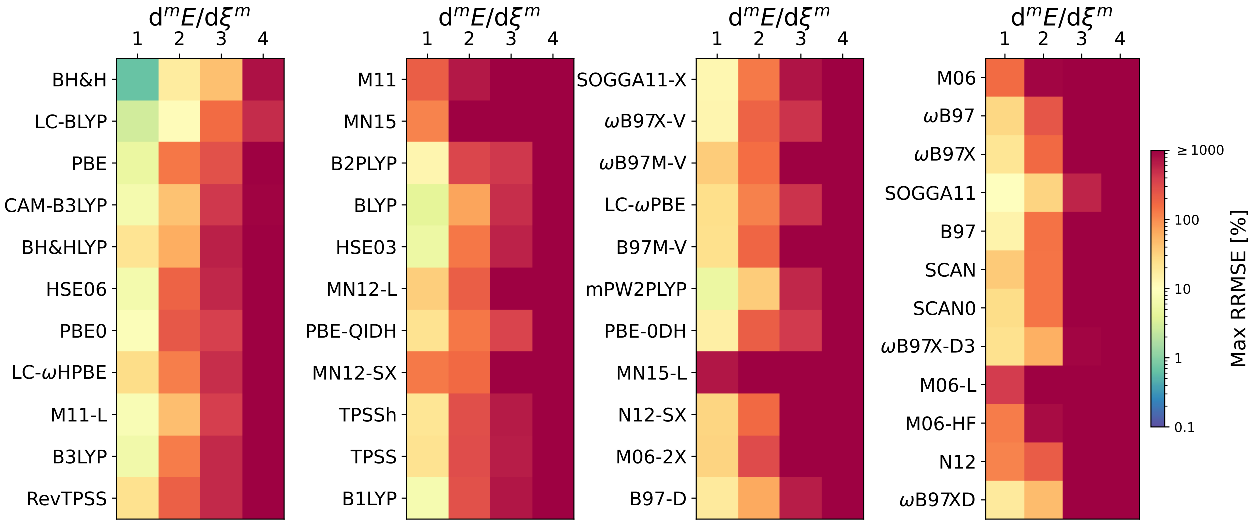

Figure S9: Maximum values of RRMSE for the derivatives of the total energy found within the set of all molecular complexes. All calculations use the (99,590) grid.

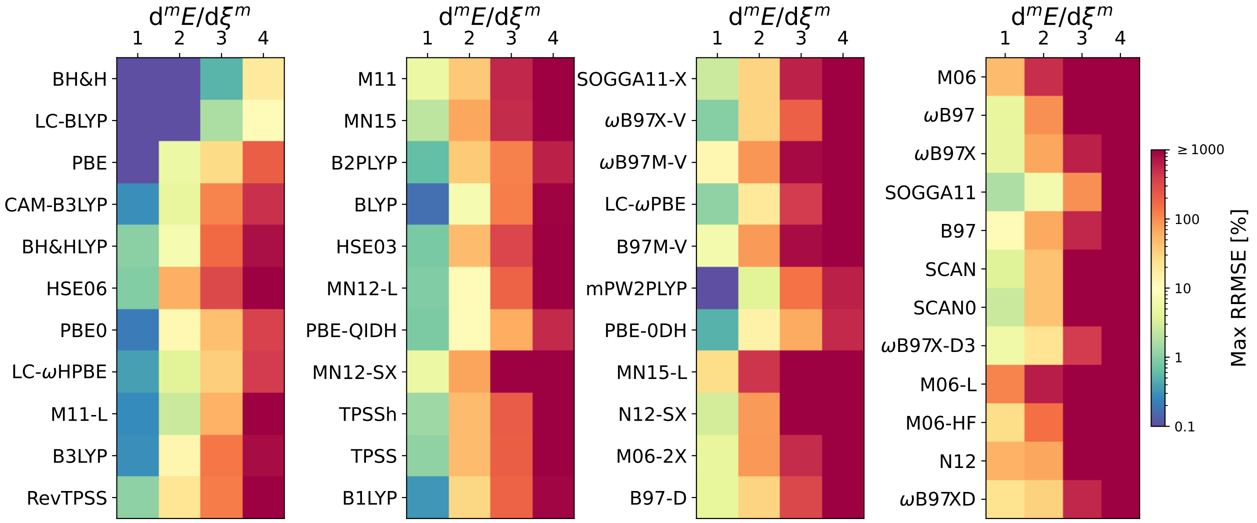

Figure S10: Maximum values of RRMSE for the derivatives of the total energy found within the set of all molecular complexes. All calculations use the (250,974) grid.

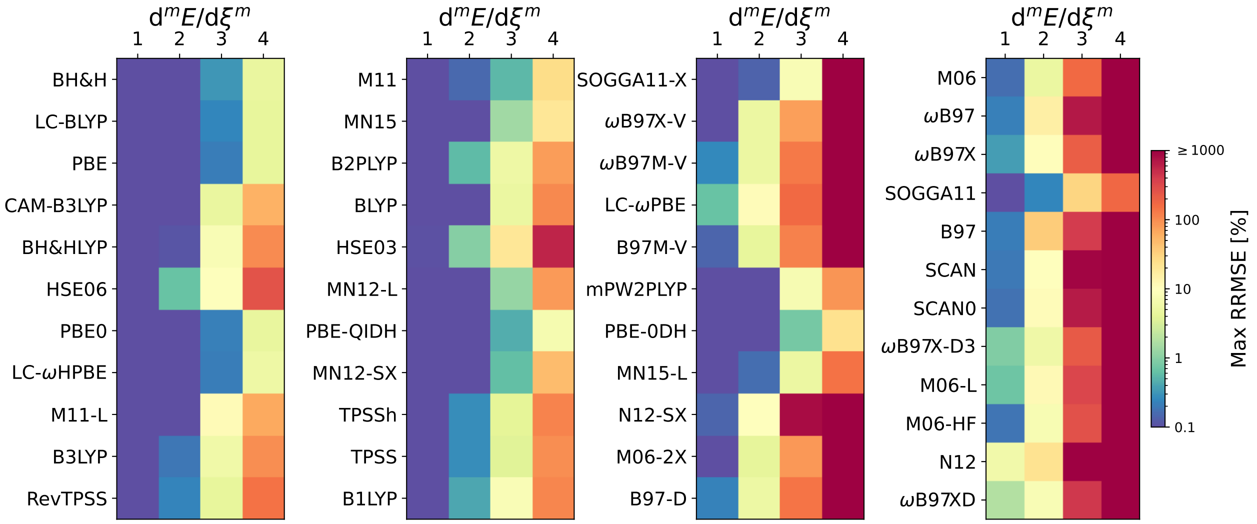

Figure S11: Maximum values of RRMSE for the derivatives of the total energy found within the set of all molecular complexes. All calculations use the (750,974) grid.

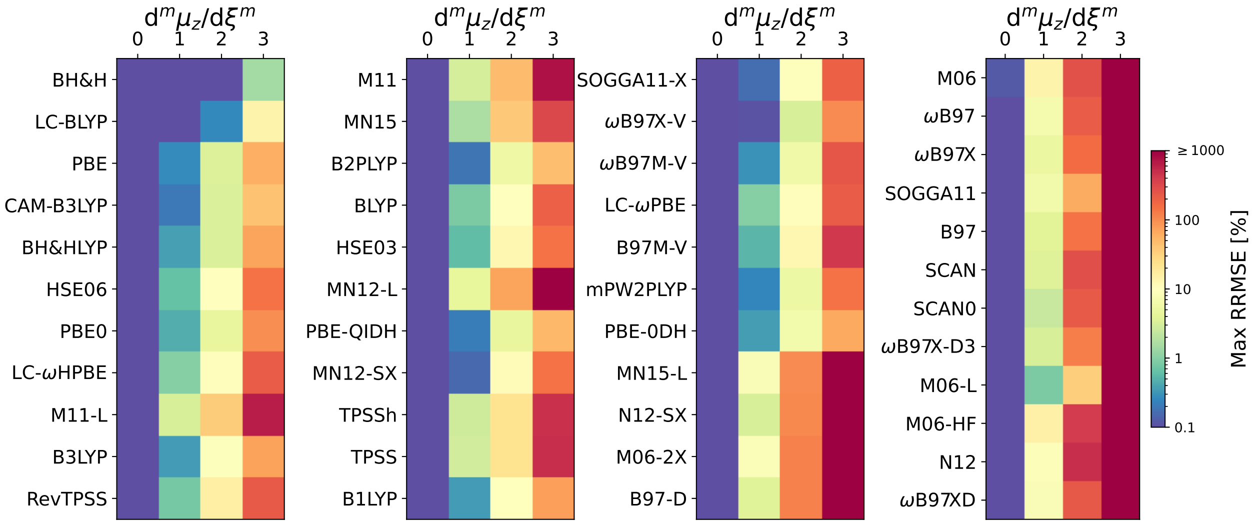

Figure S12: Maximum values of RRMSE for the derivatives of μ_z found within the set of hydrogen- and halogend-bonded molecular complexes. All calculations use the (99,590) grid.

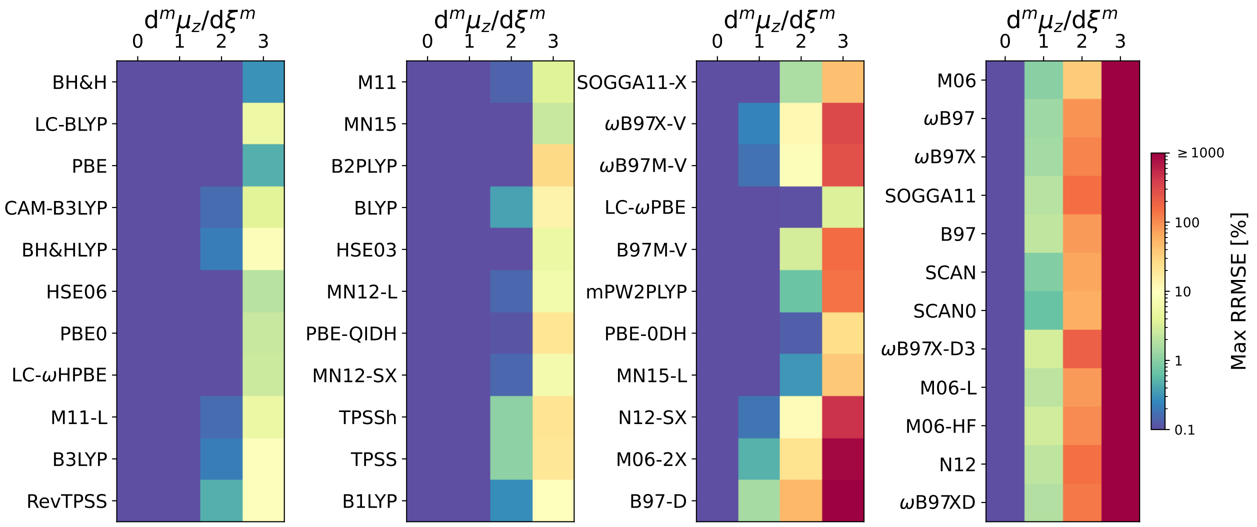

Figure S13: Maximum values of RRMSE for the derivatives of μ_z found within the set of hydrogen- and halogend-bonded molecular complexes. All calculations use the (250,974) grid.

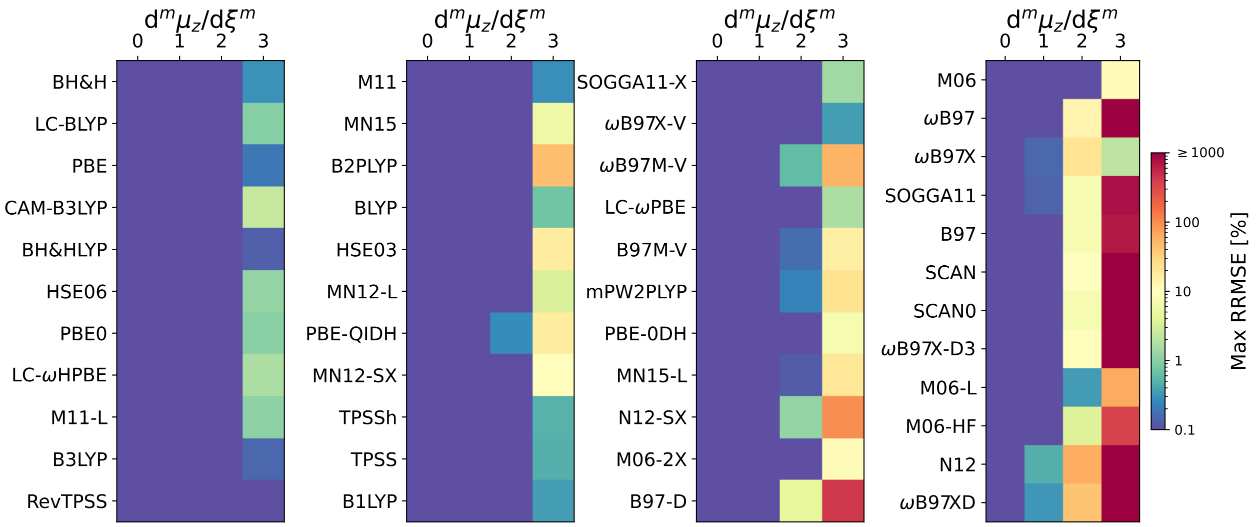

Figure S14: Maximum values of RRMSE for the derivatives of μ_z found within the set of hydrogen- and halogend-bonded molecular complexes. All calculations use the (750,974) grid.

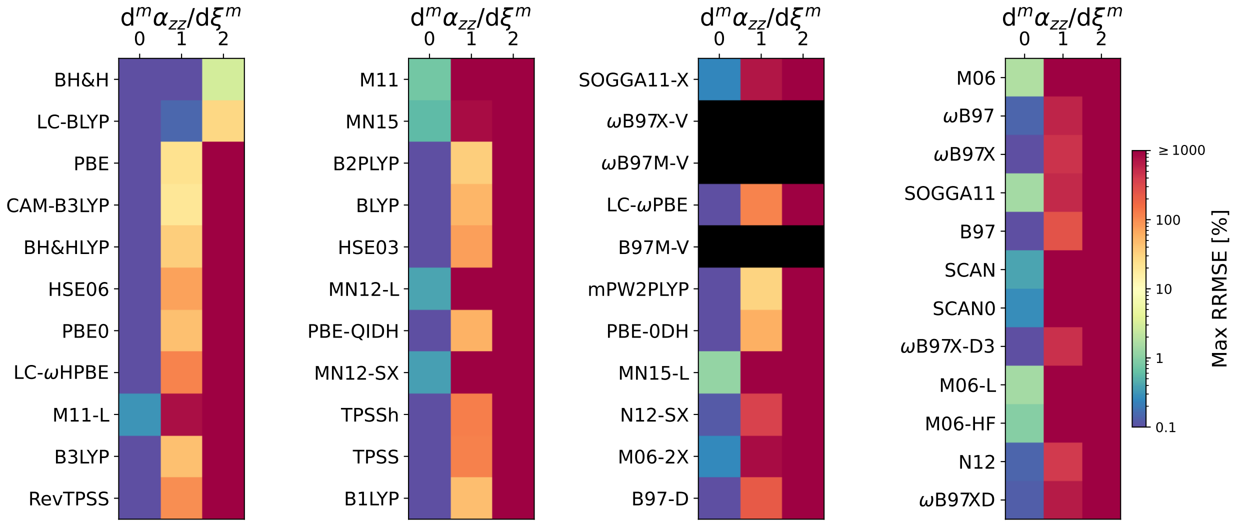

Figure S15: Maximum values of RRMSE for the derivatives of α_{zz} found within the set of all studied molecular complexes. All calculations use the (99,590) grid. Data not available for ωB97X-V, ωB97M-V and B97M-V, for which static polarizabilities are not implemented.

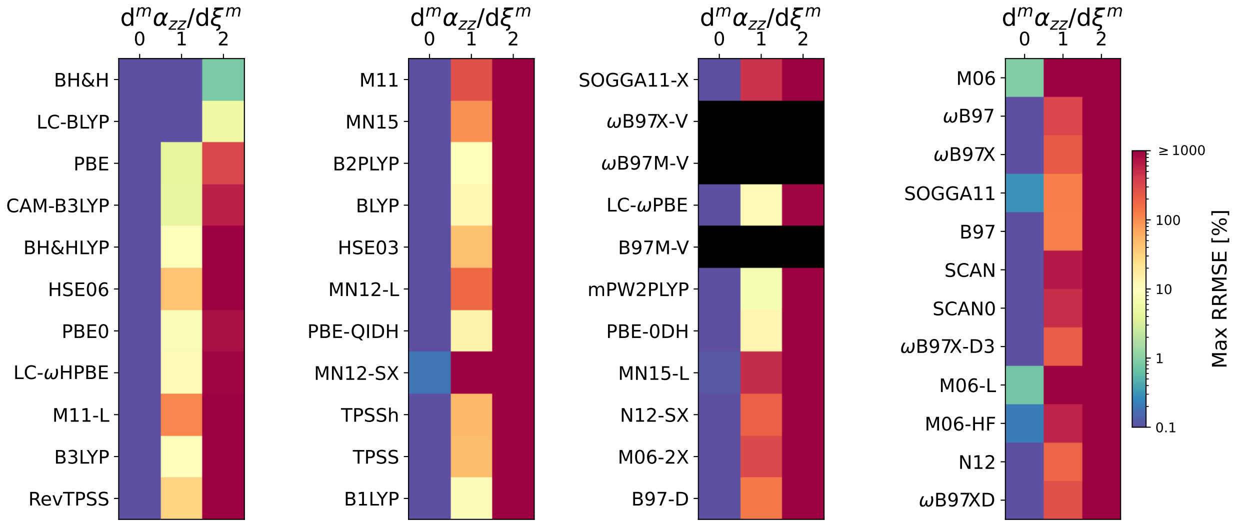

Figure S16: Maximum values of RRMSE for the derivatives of α_{zz} found within the set of all studied molecular complexes. All calculations use the (250,974) grid. Data not available for ωB97X-V, ωB97M-V and B97M-V, for which static polarizabilities are not implemented.

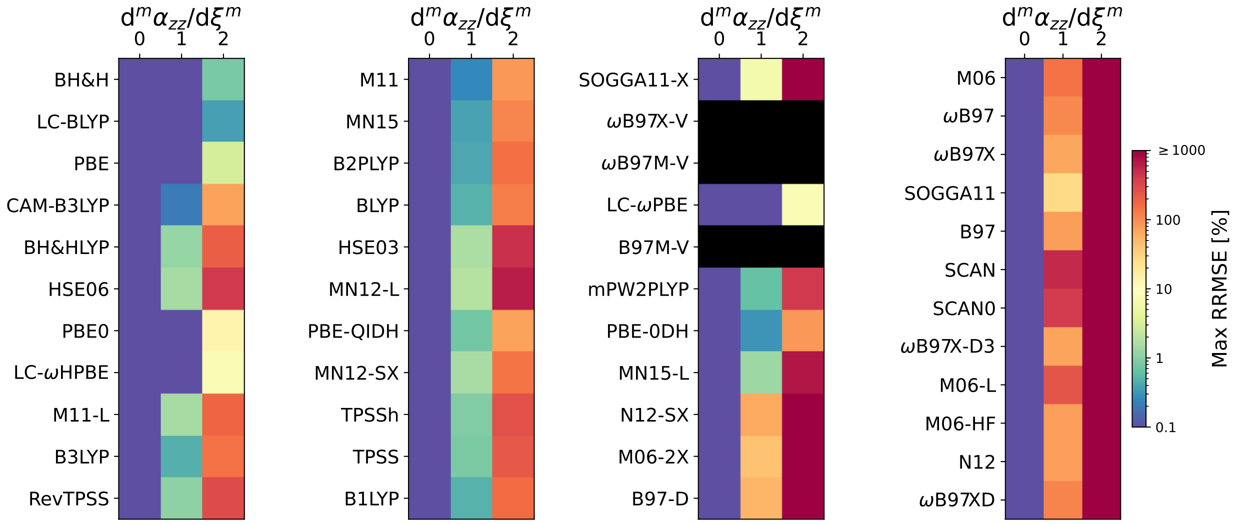

Figure S17: Maximum values of RRMSE for the derivatives of α_{zz} found within the set of all studied molecular complexes. All calculations use the (750,974) grid. Data not available for ωB97X-V, ωB97M-V and B97M-V, for which static polarizabilities are not implemented.

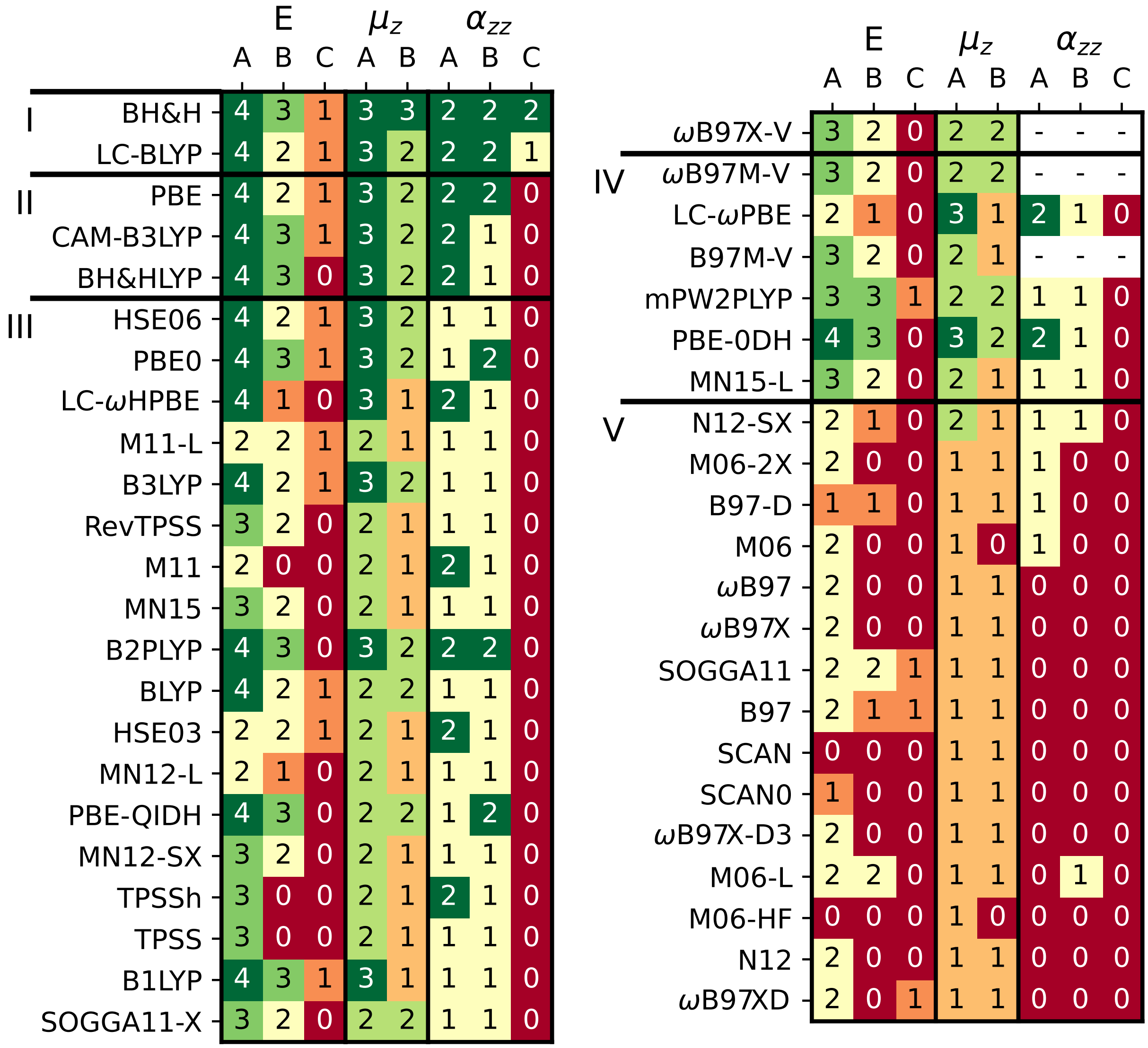

Figure S18: Lowest maximum order of derivatives that can be safely obtained with the (99,590) grid found for each group of complexes: A – hydrogen-, B – halogen-, and C – dispersion-bonded. The rungs maintain the order obtained from the (250,974) grid.

Rung	Functional	E A	E B	E C	μ_z A	μ_z B	α_{zz} A	α_{zz} B	α_{zz} C
I	BH&H	4	4	4	3	3	2	2	2
	LC-BLYP	4	4	4	3	3	2	2	2
II	PBE	4	4	4	3	3	2	2	2
	CAM-B3LYP	4	4	3	3	3	2	2	1
	BH&HLYP	4	4	3	3	3	2	2	1
III	HSE06	4	4	3	3	3	2	2	1
	PBE0	4	4	4	3	3	1	2	2
	LC-ωHPBE	4	4	4	3	3	2	2	2
	M11-L	4	4	2	3	3	2	2	1
	B3LYP	4	4	3	3	3	2	2	1
	RevTPSS	4	4	3	3	3	2	2	1
	M11	4	4	3	3	3	2	2	1
	MN15	4	4	3	3	3	1	2	1
	B2PLYP	4	4	3	2	2	2	1	1
	BLYP	4	4	3	3	3	2	2	1
	HSE03	2	2	2	2	3	2	2	1
	MN12-L	4	4	3	3	3	1	1	1
	PBE-QIDH	4	4	4	3	2	1	1	2
	MN12-SX	4	4	3	3	2	1	1	1
	TPSSh	4	4	3	3	3	2	2	1
	TPSS	4	4	3	3	3	2	2	1
	B1LYP	4	4	3	3	3	2	2	1
	SOGGA11-X	4	4	3	3	3	2	2	1

Rung	Functional	E A	E B	E C	μ_z A	μ_z B	α_{zz} A	α_{zz} B	α_{zz} C
	ωB97X-V	4	4	2	3	3	-	-	-
IV	ωB97M-V	4	3	2	3	2	-	-	-
	LC-ωPBE	3	2	1	3	3	2	2	2
	B97M-V	4	4	2	3	2	-	-	-
	mPW2PLYP	4	3	3	3	2	2	2	1
	PBE-0DH	4	3	4	3	3	1	1	2
	MN15-L	4	4	3	2	2	1	1	1
V	N12-SX	4	3	1	3	2	2	1	0
	M06-2X	4	3	2	3	2	1	2	0
	B97-D	4	3	2	3	2	2	1	0
	M06	4	4	2	3	2	1	1	0
	ωB97	4	3	1	3	1	2	1	0
	ωB97X	4	2	1	3	2	2	1	0
	SOGGA11	2	3	3	2	2	0	1	0
	B97	4	3	1	3	2	2	1	0
	SCAN	2	2	1	2	2	1	1	0
	SCAN0	2	2	1	2	2	1	1	0
	ωB97X-D3	4	2	2	3	1	2	0	0
	M06-L	4	4	1	2	2	1	2	0
	M06-HF	4	3	2	3	2	2	1	0
	N12	4	1	1	3	1	2	0	0
	ωB97XD	4	2	2	3	1	1	0	0

Figure S19: Lowest maximum order of derivatives that can be safely obtained with the (750,974) grid found for each group of complexes: A – hydrogen-, B – halogen-, and C – dispersion-bonded. The rungs maintain the order obtained from the (250,974) grid.

Table S5: Property derivatives studied in this work and the experimental molecular properties they are describing, including vibrational frequencies, IR and Raman intensities, and Nuclear Relaxation (NR) contributions to static nonlinear optical properties. In the case of higher anharmonic corrections, lower-order mechanical derivatives are also utilized.

Derivative	Molecular Property
$\mathrm{d}E/\mathrm{d}\xi$	Forces acting on nuclei
$\mathrm{d}^2E/\mathrm{d}\xi^2$	Quadratic force constants; Harmonic vibrational frequencies; NR contribution to vibrational polarizability
$\mathrm{d}^3E/\mathrm{d}\xi^3$	Cubic force constants; Cubic anharmonic correction to vibrational frequencies; Quadratic and Cubic anharmonic corrections to the IR and Raman intensities; NR contribution to vibrational hyperpolarizability
$\mathrm{d}^4E/\mathrm{d}\xi^4$	Quartic force constants; Quartic anharmonic correction to vibrational frequencies; Cubic anharmonic correction to the IR and Raman intensities; NR contribution to vibrational second hyperpolarizability
μ_i	Permanent dipole moment
$\mathrm{d}\mu_i/\mathrm{d}\xi$	Harmonic IR intensity; NR contribution to vibrational polarizability
$\mathrm{d}^2\mu_i/\mathrm{d}\xi^2$	Quadratic anharmonic correction to IR intensity; NR contribution to vibrational hyperpolarizability
$\mathrm{d}^3\mu_i/\mathrm{d}\xi^3$	Cubic anharmonic correction to IR intensity; NR contribution to vibrational second hyperpolarizability
α_{ij}	Static electronic polarizability
$\mathrm{d}\alpha_{ij}/\mathrm{d}\xi$	Harmonic Raman intensity; NR contribution to vibrational hyperpolarizability
$\mathrm{d}^2\alpha_{ij}/\mathrm{d}\xi^2$	Quadratic anharmonic correction to Raman intensity; NR contribution to vibrational second hyperpolarizability
[a] $\mathrm{d}^3\alpha_{ij}/\mathrm{d}\xi^3$	Cubic anharmonic correction to Raman intensity

[a]Derivative not studied in this work

Table S6: Maximum order m of $\mathrm{d}^m E/\mathrm{d}\xi^m$ derivatives (m = 0 - 4), for which RRMSE was lower than 10%. All calculations use the (99,590) grid.

	HCN·HF	HCN·HCl	OC·HF	N_2·HF	HCN·BrH	HCN·BrF	Ar_2	He_2
BH&H	4	4	4	4	4	3	1	2
LC-BLYP	4	4	4	4	3	2	1	2
PBE	4	4	4	4	3	2	2	1
CAM-B3LYP	4	4	4	4	3	3	1	1
BH&HLYP	4	4	4	4	3	3	2	0
HSE06	4	4	4	4	2	2	2	1
PBE0	4	4	4	4	3	3	2	1
LC-ωHPBE	4	4	4	4	1	3	1	0
M11-L	2	3	2	3	2	3	1	1
B3LYP	4	4	4	4	3	2	1	1
RevTPSS	3	4	3	4	2	2	1	0
M11	3	3	2	3	0	2	0	0
MN15	3	3	3	3	2	3	0	0
B2PLYP	4	4	4	4	3	3	2	0
BLYP	4	4	4	4	3	2	1	1
HSE03	3	3	3	2	2	2	2	1
MN12-L	3	3	2	3	1	2	0	0
PBE-QIDH	4	4	4	4	3	3	2	0
MN12-SX	3	3	3	3	2	2	0	0
TPSSh	4	4	3	4	0	2	1	0
TPSS	4	4	3	4	0	2	1	0
B1LYP	4	4	4	4	3	3	1	1
SOGGA11-X	4	3	4	3	2	2	1	0
ωB97X-V	3	3	3	3	-	2	1	0
ωB97M-V	3	3	3	3	-	2	0	0
LC-ωPBE	2	2	3	3	1	2	1	0
B97M-V	3	3	3	3	2	2	0	0
mPW2PLYP	3	3	3	3	3	3	2	1
MN15-L	3	3	3	3	2	2	0	0
PBE-0DH	4	4	4	4	3	3	2	0
N12-SX	2	2	2	2	1	2	0	1
M06-2X	2	2	2	2	0	2	0	0
B97-D	2	2	2	1	1	2	1	0
M06	2	-	2	2	0	2	0	0
ωB97	2	2	2	2	0	2	0	0
ωB97X	2	2	2	2	0	2	0	0
SOGGA11	2	-	2	2	2	2	1	1
B97	2	2	2	2	1	1	1	1
SCAN	2	0	1	1	0	0	0	1
SCAN0	2	1	1	1	2	0	0	1
ωB97X-D3	2	2	2	2	0	2	1	0
M06-L	2	2	2	2	-	2	0	0
M06-HF	2	2	2	0	0	1	0	0
N12	2	2	2	2	0	2	1	0
ωB97XD	2	2	2	2	0	0	1	1

Table S7: Maximum order m of $d^m\mu_z/d\xi^m$ derivatives (m = 0 - 3), for which RRMSE was lower than 10%. All calculations use the (99,590) grid.

	HCN·HF	HCN·HCl	OC·HF	N_2·HF	HCN·BrH	HCN·BrF
BH&H	3	3	3	3	3	3
LC-BLYP	3	3	3	3	3	2
PBE	3	3	3	3	2	3
CAM-B3LYP	3	3	3	3	2	2
BH&HLYP	3	3	3	3	2	3
HSE06	3	3	3	3	2	3
PBE0	3	3	3	3	2	3
LC-ωHPBE	3	3	3	3	1	3
M11-L	2	3	2	2	1	2
B3LYP	3	3	3	3	2	3
RevTPSS	3	3	2	3	1	3
M11	2	3	2	3	1	2
MN15	3	3	3	2	1	2
B2PLYP	3	3	3	3	2	2
BLYP	3	3	2	3	2	3
HSE03	3	3	2	3	1	3
MN12-L	3	2	2	2	1	2
PBE-QIDH	3	2	3	3	2	3
MN12-SX	3	3	2	2	1	2
TPSSh	3	3	2	3	1	3
TPSS	3	3	2	2	1	3
B1LYP	3	3	3	3	1	2
SOGGA11-X	3	3	2	2	2	2
ωB97X-V	2	2	2	2	-	2
ωB97M-V	2	2	2	2	-	2
LC-ωPBE	3	3	3	3	1	3
B97M-V	2	2	2	2	1	2
mPW2PLYP	2	2	2	2	2	2
MN15-L	2	2	2	2	1	2
PBE-0DH	3	3	3	3	2	3
N12-SX	2	2	2	2	1	1
M06-2X	2	2	1	1	1	1
B97-D	2	1	1	1	1	1
M06	2	-	2	1	0	2
ωB97	2	1	2	1	1	1
ωB97X	1	1	1	1	1	1
SOGGA11	2	-	2	1	1	2
B97	2	1	1	1	1	1
SCAN	1	1	1	1	1	1
SCAN0	1	1	1	1	1	1
ωB97X-D3	1	1	1	1	1	1
M06-L	2	1	1	1	-	1
M06-HF	2	1	1	1	0	1
N12	1	1	1	1	1	1
ωB97XD	1	1	1	1	1	1

Table S8: Maximum order m of the $\mathrm{d}^m\alpha_{zz}/\mathrm{d}\xi^m$ derivatives (m = 0 - 2), for which RRMSE was lower than 10%. All calculations use the (99,590) grid.

	HCN·HF	HCN·HCl	OC·HF	N_2·HF	HCN·BrH	HCN·BrF	Ar_2	He_2
BH&H	2	2	2	2	2	2	2	2
LC-BLYP	2	2	2	2	2	2	1	2
PBE	2	2	2	2	2	2	1	0
CAM-B3LYP	2	2	2	2	1	2	1	0
BH&HLYP	2	2	2	2	1	2	1	0
HSE06	1	2	2	2	1	2	1	0
PBE0	1	2	2	2	2	2	0	0
LC-ωHPBE	2	2	2	2	1	2	0	0
M11-L	1	2	1	1	1	2	0	0
B3LYP	1	2	2	2	1	2	1	0
RevTPSS	2	2	2	1	1	2	0	0
M11	2	2	2	2	1	2	0	0
MN15	2	2	2	1	1	2	0	0
B2PLYP	2	2	2	2	2	2	1	0
BLYP	2	2	2	1	1	2	0	0
HSE03	2	2	2	2	1	2	1	0
MN12-L	1	1	1	1	1	2	0	0
PBE-QIDH	2	1	2	2	2	2	0	0
MN12-SX	1	2	1	1	1	2	0	0
TPSSh	2	2	2	2	1	2	0	0
TPSS	2	2	2	1	1	2	0	0
B1LYP	1	2	2	2	1	2	1	0
SOGGA11-X	2	2	2	1	1	1	0	0
ωB97X-V	-	-	-	-	-	-	-	-
ωB97M-V	-	-	-	-	-	-	-	-
LC-ωPBE	2	2	2	2	1	2	0	0
B97M-V	-	-	-	-	-	-	-	-
mPW2PLYP	2	2	1	1	1	2	1	0
MN15-L	1	1	1	1	1	1	0	0
PBE-0DH	2	2	2	2	1	2	0	0
N12-SX	1	1	1	1	1	1	0	0
M06-2X	1	1	1	1	0	1	0	0
B97-D	1	1	1	1	0	1	0	0
M06	1	-	1	1	0	1	0	0
ωB97	1	1	1	0	0	1	0	0
ωB97X	1	1	1	0	0	1	0	0
SOGGA11	0	-	1	1	0	1	0	0
B97	1	1	1	0	0	1	0	0
SCAN	0	0	0	0	0	0	0	0
SCAN0	0	0	0	0	0	0	0	0
ωB97X-D3	1	1	0	0	0	1	0	0
M06-L	1	1	1	0	-	1	0	0
M06-HF	1	1	0	0	0	1	0	0
N12	1	1	1	0	0	1	0	0
ωB97XD	1	1	0	0	0	1	0	0

Table S9: Maximum order m of $\mathrm{d}^m E/\mathrm{d}\xi^m$ derivatives (m = 0 - 4), for which RRMSE was lower than 10%. All calculations use the (250,974) grid.

	HCN·HF	HCN·HCl	OC·HF	N_2·HF	HCN·BrH	HCN·BrF	Ar_2	He_2
BH&H	4	4	4	4	4	4	4	3
LC-BLYP	4	4	4	4	4	4	4	3
PBE	4	4	4	4	4	4	4	2
CAM-B3LYP	4	4	4	4	4	4	3	2
BH&HLYP	4	4	4	4	3	4	3	2
HSE06	4	4	4	4	4	4	3	1
PBE0	4	4	4	4	4	4	4	1
LC-ωHPBE	4	4	4	4	4	4	4	2
M11-L	3	3	3	3	3	4	2	2
B3LYP	4	4	4	4	3	4	3	1
RevTPSS	4	4	4	4	3	4	3	1
M11	3	3	3	3	4	3	2	1
MN15	4	4	4	4	4	4	3	1
B2PLYP	4	4	4	4	4	4	3	1
BLYP	4	4	4	4	3	4	3	2
HSE03	3	3	3	3	2	3	3	1
MN12-L	4	4	4	4	4	3	2	1
PBE-QIDH	4	4	4	4	4	4	4	1
MN12-SX	4	4	3	4	4	3	2	1
TPSSh	4	4	4	4	3	3	3	1
TPSS	4	4	4	4	3	4	3	1
B1LYP	4	4	4	4	3	4	3	1
SOGGA11-X	4	4	4	4	3	4	2	1
ωB97X-V	4	4	4	4	3	3	2	1
ωB97M-V	4	3	4	4	3	3	2	0
LC-ωPBE	2	3	3	2	2	2	2	1
B97M-V	4	3	3	4	3	3	3	1
mPW2PLYP	4	3	4	3	3	3	3	2
MN15-L	4	4	4	4	4	4	3	0
PBE-0DH	4	4	4	4	4	3	4	1
N12-SX	4	3	3	3	2	3	1	1
M06-2X	3	3	3	3	2	2	1	1
B97-D	3	3	3	2	2	3	2	1
M06	4	-	3	3	2	3	1	0
ωB97	3	2	3	2	2	2	1	1
ωB97X	3	2	3	2	2	2	1	1
SOGGA11	3	2	3	2	2	4	2	3
B97	3	3	3	3	0	2	1	1
SCAN	2	2	2	2	2	2	1	1
SCAN0	2	2	2	2	2	2	1	1
ωB97X-D3	3	2	3	2	2	2	1	1
M06-L	3	3	3	3	0	3	1	0
M06-HF	3	3	3	2	0	2	1	0
N12	3	2	3	2	0	2	1	1
ωB97XD	3	2	3	2	0	2	1	1

Table S10: Maximum order m of $d^m\mu_z/d\xi^m$ derivatives (m = 0 - 3), for which RRMSE was lower than 10%. All calculations use the (250,974) grid.

	HCN·HF	HCN·HCl	OC·HF	N_2·HF	HCN·BrH	HCN·BrF
BH&H	3	3	3	3	3	3
LC-BLYP	3	3	3	3	3	3
PBE	3	3	3	3	3	3
CAM-B3LYP	3	3	3	3	3	3
BH&HLYP	3	3	3	3	3	3
HSE06	3	3	3	3	3	3
PBE0	3	3	3	3	3	3
LC-ωHPBE	3	3	3	3	3	3
M11-L	3	3	3	3	3	3
B3LYP	3	3	3	3	3	3
RevTPSS	3	3	3	3	3	3
M11	3	3	3	3	3	3
MN15	3	3	3	3	3	3
B2PLYP	2	3	3	3	3	2
BLYP	3	3	3	3	2	3
HSE03	3	3	3	3	3	3
MN12-L	3	3	3	3	3	3
PBE-QIDH	2	2	3	3	3	3
MN12-SX	3	3	3	3	3	3
TPSSh	3	3	3	3	2	3
TPSS	3	3	3	3	2	3
B1LYP	3	3	3	3	2	3
SOGGA11-X	3	3	3	3	2	3
ωB97X-V	3	3	3	3	1	3
ωB97M-V	3	3	3	3	2	2
LC-ωPBE	3	3	3	3	3	3
B97M-V	3	3	2	3	2	2
mPW2PLYP	3	2	2	2	2	2
MN15-L	2	3	3	3	3	2
PBE-0DH	2	3	3	3	3	2
N12-SX	2	2	2	2	1	2
M06-2X	3	2	2	2	1	2
B97-D	2	2	2	2	1	2
M06	3	-	2	2	1	2
ωB97	2	2	2	2	1	1
ωB97X	2	2	2	2	1	1
SOGGA11	1	2	3	2	1	2
B97	2	2	2	2	1	2
SCAN	1	1	1	1	1	1
SCAN0	1	1	1	1	1	1
ωB97X-D3	2	2	2	1	1	1
M06-L	2	2	2	2	1	3
M06-HF	2	2	2	2	1	2
N12	2	2	2	1	1	1
ωB97XD	2	2	2	1	1	1

Table S11: Maximum order m of the $d^m \alpha_{zz}/d\xi^m$ derivatives (m = 0 - 2), for which RRMSE was lower than 10%. All calculations use the (250,974) grid.

	HCN · HF	HCN · HCl	OC · HF	N_2 · HF	HCN · BrH	HCN · BrF	Ar_2	He_2
BH&H	2	2	2	2	2	2	2	2
LC-BLYP	2	2	2	2	2	2	2	2
PBE	2	2	2	2	2	2	1	1
CAM-B3LYP	2	2	2	2	2	2	1	1
BH&HLYP	2	2	2	2	2	2	1	1
HSE06	2	2	2	2	2	2	1	0
PBE0	1	2	2	2	2	2	1	1
LC-ωHPBE	1	2	2	2	2	2	1	0
M11-L	2	2	2	2	2	2	1	0
B3LYP	2	2	2	2	2	2	1	0
RevTPSS	2	2	2	2	2	2	1	0
M11	2	2	2	2	2	2	1	0
MN15	1	2	2	2	2	2	1	0
B2PLYP	2	2	2	2	2	2	1	1
BLYP	2	2	2	2	2	2	1	0
HSE03	2	2	2	2	2	2	1	0
MN12-L	1	2	2	2	2	2	1	0
PBE-QIDH	1	1	2	2	2	2	1	0
MN12-SX	1	2	2	2	2	2	0	0
TPSSh	2	2	2	2	1	2	1	0
TPSS	2	2	2	2	1	2	1	0
B1LYP	1	2	2	2	2	2	1	0
SOGGA11-X	2	2	2	2	1	2	1	0
ωB97X-V	-	-	-	-	-	-	-	-
ωB97M-V	-	-	-	-	-	-	-	-
LC-ωPBE	1	2	2	2	2	2	1	0
B97M-V	-	-	-	-	-	-	-	-
mPW2PLYP	2	2	1	1	1	1	1	1
MN15-L	1	2	2	2	1	1	1	0
PBE-0DH	1	2	2	2	1	2	1	0
N12-SX	2	1	1	1	1	2	0	0
M06-2X	2	1	1	1	1	2	0	0
B97-D	1	1	1	1	1	2	0	0
M06	1	-	2	1	1	2	0	0
ωB97	1	1	1	1	0	1	0	0
ωB97X	1	1	1	1	0	1	0	0
SOGGA11	0	1	1	1	1	1	0	0
B97	2	1	1	1	0	1	0	0
SCAN	0	0	0	0	1	1	0	0
SCAN0	0	1	0	0	1	1	0	0
ωB97X-D3	1	1	1	1	0	1	0	0
M06-L	1	1	1	1	0	1	0	0
M06-HF	2	1	1	1	0	1	0	0
N12	1	1	1	1	0	1	0	0
ωB97XD	2	1	1	1	0	1	0	0

Table S12: Maximum order m of $d^m E/d\xi^m$ derivatives (m = 0 - 4), for which RRMSE was lower than 10%. All calculations use the (750,974) grid.

	HCN·HF	HCN·HCl	OC·HF	N_2·HF	HCN·BrH	HCN·BrF	Ar_2	He_2
BH&H	4	4	4	4	4	4	4	4
LC-BLYP	4	4	4	4	4	4	4	4
PBE	4	4	4	4	4	4	4	4
CAM-B3LYP	4	4	4	4	4	4	4	3
BH&HLYP	4	4	4	4	4	4	4	3
HSE06	4	4	4	4	4	4	4	3
PBE0	4	4	4	4	4	4	4	4
LC-ωHPBE	4	4	4	4	4	4	4	4
M11-L	4	4	4	4	4	4	4	2
B3LYP	4	4	4	4	4	4	4	3
RevTPSS	4	4	4	4	4	4	4	3
M11	4	4	4	4	4	4	4	3
MN15	4	4	4	4	4	4	4	3
B2PLYP	4	4	4	4	4	4	4	3
BLYP	4	4	4	4	4	4	4	3
HSE03	3	3	2	3	3	2	4	2
MN12-L	4	4	4	4	4	4	4	3
PBE-QIDH	4	4	4	4	4	4	4	4
MN12-SX	4	4	4	4	4	4	4	3
TPSSh	4	4	4	4	4	4	4	3
TPSS	4	4	4	4	4	4	4	3
B1LYP	4	4	4	4	4	4	4	3
SOGGA11-X	4	4	4	4	4	4	4	3
ωB97X-V	4	4	4	4	4	4	4	2
ωB97M-V	4	4	4	4	3	4	2	2
LC-ωPBE	3	3	3	3	2	3	2	1
B97M-V	4	4	4	4	4	4	4	2
mPW2PLYP	4	4	4	4	3	4	3	3
MN15-L	4	4	4	4	4	4	4	3
PBE-0DH	4	4	4	4	4	3	4	4
N12-SX	4	4	4	4	3	4	2	1
M06-2X	4	4	4	4	4	3	2	2
B97-D	4	4	4	4	3	4	2	2
M06	4	-	4	4	4	4	2	2
ωB97	4	4	4	4	3	4	2	1
ωB97X	4	4	4	4	2	4	2	1
SOGGA11	3	3	4	2	3	4	3	3
B97	4	4	4	4	3	3	2	1
SCAN	3	2	2	2	2	2	2	1
SCAN0	3	2	2	2	2	3	2	1
ωB97X-D3	4	4	4	4	2	4	2	2
M06-L	4	4	4	4	4	4	2	1
M06-HF	4	4	4	4	3	3	2	2
N12	4	4	4	4	1	4	2	1
ωB97XD	4	4	4	4	2	4	2	2

Table S13: Maximum order m of $d^m\mu_z/d\xi^m$ derivatives (m = 0 - 3), for which RRMSE was lower than 10%. All calculations use the (750,974) grid.

	HCN · HF	HCN · HCl	OC · HF	N_2 · HF	HCN · BrH	HCN · BrF
BH&H	3	3	3	3	3	3
LC-BLYP	3	3	3	3	3	3
PBE	3	3	3	3	3	3
CAM-B3LYP	3	3	3	3	3	3
BH&HLYP	3	3	3	3	3	3
HSE06	3	3	3	3	3	3
PBE0	3	3	3	3	3	3
LC-ωHPBE	3	3	3	3	3	3
M11-L	3	3	3	3	3	3
B3LYP	3	3	3	3	3	3
RevTPSS	3	3	3	3	3	3
M11	3	3	3	3	3	3
MN15	3	3	3	3	3	3
B2PLYP	2	3	3	3	3	2
BLYP	3	3	3	3	3	3
HSE03	3	3	2	3	3	3
MN12-L	3	3	3	3	3	3
PBE-QIDH	3	3	3	3	2	3
MN12-SX	3	3	3	3	2	3
TPSSh	3	3	3	3	3	3
TPSS	3	3	3	3	3	3
B1LYP	3	3	3	3	3	3
SOGGA11-X	3	3	3	3	3	3
ωB97X-V	3	3	3	3	3	3
ωB97M-V	3	3	3	3	2	3
LC-ωPBE	3	3	3	3	3	3
B97M-V	3	3	3	3	2	3
mPW2PLYP	3	3	3	3	2	2
MN15-L	2	3	3	3	3	2
PBE-0DH	3	3	3	3	3	3
N12-SX	3	3	3	3	2	3
M06-2X	3	3	3	3	2	3
B97-D	3	3	3	3	2	3
M06	3	-	3	3	2	3
ωB97	3	3	3	3	1	3
ωB97X	3	3	3	3	2	3
SOGGA11	2	3	3	2	2	2
B97	3	3	3	3	2	3
SCAN	2	2	2	2	2	2
SCAN0	2	2	2	2	2	2
ωB97X-D3	3	3	3	3	1	3
M06-L	2	3	2	3	2	3
M06-HF	3	3	3	3	2	3
N12	3	3	3	3	1	3
ωB97XD	3	3	3	3	1	3

Table S14: Maximum order m of the $d^m \alpha_{zz}/d\xi^m$ derivatives (m = 0 - 2), for which RRMSE was lower than 10%. All calculations use the (750,974) grid.

	$HCN \cdot HF$	$HCN \cdot HCl$	$OC \cdot HF$	$N_2 \cdot HF$	$HCN \cdot BrH$	$HCN \cdot BrF$	Ar_2	He_2
BH&H	2	2	2	2	2	2	2	2
LC-BLYP	2	2	2	2	2	2	2	2
PBE	2	2	2	2	2	2	2	2
CAM-B3LYP	2	2	2	2	2	2	1	1
BH&HLYP	2	2	2	2	2	2	1	1
HSE06	2	2	2	2	2	2	2	1
PBE0	1	2	2	2	2	2	2	2
LC-ωHPBE	2	2	2	2	2	2	2	2
M11-L	2	2	2	2	2	2	2	1
B3LYP	2	2	2	2	2	2	1	1
RevTPSS	2	2	2	2	2	2	2	1
M11	2	2	2	2	2	2	2	1
MN15	1	2	2	2	2	2	2	1
B2PLYP	2	2	2	2	1	2	1	1
BLYP	2	2	2	2	2	2	1	1
HSE03	2	2	2	2	2	2	2	1
MN12-L	1	2	2	2	1	2	2	1
PBE-QIDH	1	1	2	2	1	2	2	2
MN12-SX	1	2	2	2	1	2	2	1
TPSSh	2	2	2	2	2	2	2	1
TPSS	2	2	2	2	2	2	2	1
B1LYP	2	2	2	2	2	2	1	1
SOGGA11-X	2	2	2	2	2	2	2	1
ωB97X-V	-	-	-	-	-	-	-	-
ωB97M-V	-	-	-	-	-	-	-	-
LC-ωPBE	2	2	2	2	2	2	2	2
B97M-V	-	-	-	-	-	-	-	-
mPW2PLYP	2	2	2	2	2	2	1	1
MN15-L	1	2	2	2	1	1	2	1
PBE-0DH	1	2	2	2	1	2	2	2
N12-SX	2	2	2	2	1	2	0	0
M06-2X	2	2	2	1	2	2	1	0
B97-D	2	2	2	2	1	1	0	0
M06	1	-	2	2	1	2	0	0
ωB97	2	2	2	2	1	2	0	0
ωB97X	2	2	2	2	1	2	0	0
SOGGA11	0	1	1	2	1	1	1	0
B97	2	2	2	2	1	2	0	0
SCAN	1	1	1	1	1	1	1	0
SCAN0	1	1	1	1	1	1	1	0
ωB97X-D3	2	2	2	2	0	2	0	0
M06-L	1	1	2	2	2	2	0	0
M06-HF	2	2	2	2	1	2	1	0
N12	2	2	2	2	0	1	0	0
ωB97XD	2	2	2	1	0	2	0	0

Table S15: (An)harmonic vibrational properties of the intermolecular stretching mode of $N_2 \cdot HF$: ω_{har} and ω_{anh} - harmonic and anharmonic vibrational frequencies, IR_{har} and IR_{anh} - harmonic and anharmonic IR intensities, RA_{har} and RA_{anh} - harmonic and anharmonic Raman intensities. DFAs results combined with the (750,974) integration grid.

			$N_2 \cdot HF$			
	ω_{har} [cm^{-1}]	ω_{anh} [cm^{-1}]	IR_{har} [km mol^{-1}]	IR_{anh} [km mol^{-1}]	RA_{har} [Å^6]	RA_{anh} [Å^6]
B3LYP	117.4	82.5	1.72	1.28	6.5E-03	9.6E-03
CAM-B3LYP	129.6	96.4	1.89	1.47	5.3E-03	7.3E-03
N12	121.5	85.8	2.05	1.58	8.4E-03	1.2E-02
M062X	123.5	84.7	1.51	1.06	4.9E-03	7.5E-03
ωB97X	137.9	105.1	1.81	1.43	4.6E-03	6.1E-03

Table S16: RRMSE of various derivatives of E, μ_z, and α_{zz} with respect to the floppy intermolecular stretching mode Q_3 of $N_2 \cdot HF$ using three integration grids: G1 - (99,590), G2 - (250,974) and G3 - (500,974). The last 6 columns contain the relative errors (in %) of the selected (an)harmonic vibrational properties: ω_{har} and $\Delta\omega_{\mathrm{anh}}$- harmonic vibrational frequency and its anharmonic correction; $\mathrm{IR}_{\mathrm{har}}$ and $\Delta\mathrm{IR}_{\mathrm{anh}}$ - harmonic IR intensities and its anharmonic correction; $\mathrm{RA}_{\mathrm{har}}$ and $\mathrm{RA}_{\mathrm{anh}}$ - harmonic Raman intensities and its anharmonic correction. The error estimations were obtained with the (750,974) grid results used as the reference.

	RRMSE [%]									Prop. Rel. Err. [%]					
	$\mathrm{d}^2E/\mathrm{d}\chi_{1,z}{}^2$			$\mathrm{d}^3E/\mathrm{d}\chi_{1,z}{}^3$			$\mathrm{d}^4E/\mathrm{d}\chi_{1,z}{}^4$			$\omega_{\mathrm{har}}(Q_3)$			$\Delta\omega_{\mathrm{anh}}(Q_3)$		
	G1	G2	G3	G1	G2	G3	G1	G2	G3	G1	G2	G3	G1	G2	G3
B3LYP	0	0	0	0	0	0	4	0	0	0	0	0	2	0	0
CAM-B3LYP	0	0	0	0	0	0	2	0	0	0	0	0	1	0	0
N12	7	1	0	119	31	2	1973	874	70	2	0	0	625	175	0
M062X	2	0	0	21	3	0	388	70	1	1	0	0	62	14	0
ωB97X	3	1	0	52	16	1	1447	665	48	2	0	0	195	234	9

	RRMSE [%]									Prop. Rel. Err. [%]					
	$\mathrm{d}^1\mu_z/\mathrm{d}\chi_{1,z}{}^1$			$\mathrm{d}^2\mu_z/\mathrm{d}\chi_{1,z}{}^2$			$\mathrm{d}^3\mu_z/\mathrm{d}\chi_{1,z}{}^3$			$\mathrm{IR}_{\mathrm{har}}(Q_3)$			$\Delta\mathrm{IR}_{\mathrm{anh}}(Q_3)$		
	G1	G2	G3	G1	G2	G3	G1	G2	G3	G1	G2	G3	G1	G2	G3
B3LYP	0	0	0	0	0	0	7	0	0	0	0	0	1	0	0
CAM-B3LYP	0	0	0	0	0	0	3	0	0	0	0	0	0	0	0
N12	1	0	0	56	14	1	4548	1983	148	2	0	0	303	62	4
M062X	0	0	0	12	1	0	373	42	1	0	0	0	9	1	0
ωB97X	0	0	0	26	9	1	1650	794	50	2	0	0	31	126	4

	RRMSE [%]						Prop. Rel. Err. [%]					
	$\mathrm{d}^1\alpha_{zz}/\mathrm{d}\chi_{1,z}{}^1$			$\mathrm{d}^2\alpha_{zz}/\mathrm{d}\chi_{1,z}{}^2$			$\mathrm{RA}_{\mathrm{har}}(Q_3)$			$\Delta\mathrm{RA}_{\mathrm{anh}}(Q_3)$		
	G1	G2	G3	G1	G2	G3	G1	G2	G3	G1	G2	G3
B3LYP	0	0	0	5	0	0	0	0	0	6	0	0
CAM-B3LYP	0	0	0	2	0	0	0	0	0	4	0	0
N12	17	4	0	8588	3726	257	38	11	1	32656	15921	192
M062X	5	0	0	907	114	29	11	0	0	171	110	9
ωB97X	12	4	0	2103	973	64	15	0	0	60435	915	163

Table S17: Relative errors with respect to CCSD(T) of the electronic (el) and nuclear relaxation (nr) contributions to various nonlinear optical properties of $N_2 \cdot HF$ using three different DFAs and two integration grids: (750,974) and SuperFineGrid.[8] Data for the SuperFineGrid was taken from Ref. 72, whereas data for the (750,974) grid was recalculated in this work using the same methodology.

	$\alpha^{\mathrm{el}}_{zz}$	$\beta^{\mathrm{el}}_{zzz}$	$\gamma^{\mathrm{el}}_{zzzz}$	α^{r}_{zz}	$\beta^{\mathrm{nr}}_{zzz}$	$\gamma^{\mathrm{nr}}_{zzzz}$
M06(SuperFineGrid)[a]	2	68	58	1	11	767
M06(750,974)	2	60	36	1	36	83
M062X(SuperFineGrid)[a]	1	15	5	1	103	2296
M062X(750,974)	1	19	3	0	21	119
ωB97X(SuperFineGrid)[a]	2	6	5	4	140	5483
ωB97X(750,974)	2	9	6	1	1	21

[a] Taken from Ref. 72

Table S18: RRMSE of various derivatives of E, μ_z, and α_{zz} for the intermolecular stretching of HCN$\cdot$BrH using four integration grids: G1 - (99,590), G2 - (250,974), G3 - (500,974), G4 - (750,974), and G5 - (999,974). The last 10 columns contain the values of selected (an)harmonic vibrational properties: ω_{har} and $\Delta\omega_{\text{anh}}$- harmonic vibrational frequency and its anharmonic correction; IR_{har} and $\Delta\text{IR}_{\text{anh}}$ - harmonic IR intensities and its anharmonic correction; RA_{har} and RA_{anh} - harmonic Raman intensities and its anharmonic correction.

	RRMSE [%]												$\omega_{\text{har}}(Q_4)$ [cm^{-1}]					$\Delta\omega_{\text{anh}}(Q_4)$ [cm^{-1}]				
	$d^2E/d\chi_{1,z}{}^2$				$d^3E/d\chi_{1,z}{}^3$				$d^4E/d\chi_{1,z}{}^4$													
	G1	G2	G3	G4	G1	G2	G3	G4	G1	G2	G3	G4	G1	G2	G3	G4	G5	G1	G2	G3	G4	G5
B3LYP	0.6	0.1	0.0	0.0	4.2	1.2	0.1	0.0	19.6	13.4	5.6	0.2	44.9	45.0	45.0	45.0	45.0	-10.9	-10.6	-1.7	-8.9	-6.9
BH&H	0.2	0.0	0.0	0.0	1.2	0.0	0.0	0.0	6.7	0.4	1.4	1.0	90.0	90.1	90.1	90.1	90.1	-22.5	-23.4	-25.3	-20.7	-20.0
N12	95.6	69.1	21.4	22.2	1430.0	863.5	393.0	616.7	3.2E4	2.0E4	1.3E4	2.2E4	44.5	36.9	21.7	25.3	26.4	-1586.9	-269.9	1270.6	-959.0	-728.5
M062X	11.9	4.9	0.3	0.0	72.5	57.8	6.2	0.0	1067.6	780.0	194.6	0.3	66.2	63.4	60.8	60.9	60.9	17.2	-73.0	-5.3	-17.0	-7.2
ωB97X	18.0	7.9	3.8	0.4	184.6	126.2	101.6	14.4	5146.8	3393.1	3527.4	764.7	86.2	76.6	72.4	74.3	74.5	-222.5	-168.5	172.4	27.1	-8.8

	RRMSE [%]												$\text{IR}_{\text{har}}(Q_4)$ [km mol^{-1}]					$\Delta\text{IR}_{\text{anh}}(Q_4)$ [km mol^{-1}]				
	$d^1\mu_z/d\chi_{1,z}{}^1$				$d^2\mu_z/d\chi_{1,z}{}^2$				$d^3\mu_z/d\chi_{1,z}{}^3$													
	G1	G2	G3	G4	G1	G2	G3	G4	G1	G2	G3	G4	G1	G2	G3	G4	G5	G1	G2	G3	G4	G5
B3LYP	0.3	0.0	0.0	0.0	9.0	0.2	0.0	0.0	74.2	9.2	2.2	0.2	0.28	0.28	0.28	0.28	0.28	-0.09	-0.08	-0.08	-0.08	-0.07
BH&H	0.0	0.0	0.0	0.0	0.0	0.0	0.0	0.0	0.2	0.1	0.1	0.1	0.74	0.74	0.74	0.74	0.74	-0.24	-0.24	-0.24	-0.24	-0.24
N12	8.8	2.2	0.7	0.5	500.0	150.7	60.7	61.3	2.8E4	1.1E4	4932.4	6243.5	0.30	0.20	0.18	0.19	0.19	116.83	5.13	6658.52	1348.11	838.45
M062X	8.6	0.5	0.0	0.0	116.8	22.6	3.6	0.1	4338.8	881.2	256.1	10.8	0.48	0.39	0.39	0.39	0.39	-0.05	-0.29	-0.14	-0.14	-0.13
ωB97X	5.2	1.5	0.9	0.2	162.9	112.2	88.1	21.5	7145.2	6359.2	5427.5	1971.6	0.53	0.45	0.46	0.46	0.46	2.17	1.91	-0.22	-0.12	-0.09

	RRMSE [%]								$\text{RA}_{\text{har}}(Q_4)$ [Å^6]					$\Delta\text{RA}_{\text{anh}}(Q_4)$ [Å^6]				
	$d^1\alpha_{zz}/d\chi_{1,z}{}^1$				$d^2\alpha_{zz}/d\chi_{1,z}{}^2$													
	G1	G2	G3	G4	G1	G2	G3	G4	G1	G2	G3	G4	G5	G1	G2	G3	G4	G5
B3LYP	0.6	0.1	0.0	0.0	12.8	4.0	0.7	0.2	1.0E-01	1.0E-01	1.0E-01	1.0E-01	1.0E-01	1.3E-02	5.5E-03	-1.7E-02	-4.2E-04	-2.1E-03
BH&H	0.0	0.0	0.0	0.0	0.0	0.0	0.0	0.0	9.0E-02	8.9E-02	8.9E-02	8.9E-02	8.9E-02	5.1E-03	6.6E-03	7.7E-03	4.4E-03	2.7E-03
N12	61.8	38.6	17.3	26.0	4746.6	2938.1	1949.8	3194.4	6.1E-03	1.1E-01	2.3E-01	3.3E-01	1.5E-01	1.9E+01	1.2E+02	8.1E+00	3.1E+03	3.6E+02
M062X	10.4	7.3	0.8	0.0	622.1	370.5	100.4	1.3	8.1E-02	7.7E-02	7.8E-02	7.9E-02	7.9E-02	3.5E-01	-2.1E-02	1.8E-01	1.9E-03	-2.1E-03
ωB97X	28.9	18.4	13.9	1.8	2509.4	1516.8	1505.9	302.4	4.1E-02	2.8E-02	7.1E-02	5.7E-02	5.9E-02	7.0E-01	6.6E+00	2.8E+00	7.5E+02	4.0E+03

Table S19: Harmonic (ω_{har}) and anharmonic (ω_{anh}) vibrational frequencies for all normal modes of HCN $\cdot$ BrH, obtained with VPT2 and the various DFAs combined with the five integration grids: G1 - (99,590), G2 - (250,974), G3 - (500,974), G4 - (750,974), and G5 - (999,974). Modes Q_1-Q_4 are of the σ-type and modes Q_5 - Q_7 are of the π-type. Moreover, Q_4 is the intermolecular streching mode, which has the dominating contribution in the FIC1 adopted in this work. The red color indicates the anharmonic frequencies that are hugely affected by the spurious oscillations; notice the magnitude and the sign of the error changes dramatically.

B3LYP

	ω_{har} [cm^{-1}]					ω_{anh} [cm^{-1}]				
Mode	G1	G2	G3	G4	G5	G1	G2	G3	G4	G5
Q_1	3440.0	3440.3	3440.3	3440.3	3440.3	3319.8	3311.1	3312.2	3311.9	3313.3
Q_2	2612.4	2613.5	2613.5	2613.5	2613.5	2546.7	2527.2	2526.5	2526.9	2527.2
Q_3	2203.4	2203.3	2203.3	2203.3	2203.3	2175.8	2175.7	2176.1	2175.9	2175.9
Q_4	44.9	45.0	45.0	45.0	45.0	34.0	34.4	43.3	36.1	38.1
Q_5	761.8	761.8	761.8	761.8	761.8	750.7	746.9	746.9	746.9	747.0
Q_6	184.4	185.2	185.2	185.1	185.2	140.5	127.4	126.6	126.5	126.9
Q_7	32.4	32.5	32.5	32.5	32.5	11.2	7.1	6.5	7.3	8.8

BH&H

	ω_{har} [cm^{-1}]					ω_{anh} [cm^{-1}]				
Mode	G1	G2	G3	G4	G5	G1	G2	G3	G4	G5
Q_1	3505.0	3505.1	3505.1	3505.1	3505.1	3385.5	3384.2	3383.2	3383.4	3382.9
Q_2	2709.4	2711.7	2711.7	2711.7	2711.7	2671.7	2633.7	2633.6	2633.9	2633.7
Q_3	2321.4	2321.4	2321.4	2321.4	2321.4	2294.7	2295.1	2294.6	2295.1	2294.9
Q_4	90.0	90.1	90.1	90.1	90.1	67.4	66.7	64.8	69.4	70.0
Q_5	807.6	807.6	807.6	807.6	807.6	790.3	789.4	789.4	789.4	789.4
Q_6	260.0	259.7	259.7	259.7	259.7	181.8	194.4	194.2	194.2	194.2
Q_7	32.8	32.7	32.7	32.7	32.7	8.9	13.9	12.9	13.5	13.1

N12

	ω_{har} [cm^{-1}]					ω_{anh} [cm^{-1}]				
Mode	G1	G2	G3	G4	G5	G1	G2	G3	G4	G5
Q_1	3440.1	3441.8	3441.8	3441.8	3441.8	3308.6	3305.3	3305.9	3304.4	3304.7
Q_2	2638.4	2644.0	2644.1	2644.1	2644.1	2611.5	2545.6	2541.9	2529.3	2545.5
Q_3	2167.5	2166.1	2165.9	2165.9	2166.0	2098.0	2130.7	2150.2	2132.8	2130.1
Q_4	44.5	36.9	21.7	25.3	26.4	**-1542.4**	**-233.0**	**1292.3**	**-933.8**	**-702.0**
Q_5	738.9	738.5	738.5	738.5	738.5	681.1	678.7	678.5	678.9	678.4
Q_6	192.9	173.0	171.9	171.8	172.1	**-1253.0**	**-487.3**	**-496.9**	**-496.7**	**-494.2**
Q_7	36.3	32.3	32.2	32.1	32.2	**-380.0**	**-174.4**	**-171.9**	**-162.7**	**-175.1**

M062X

	ω_{har} [cm^{-1}]					ω_{anh} [cm^{-1}]				
Mode	G1	G2	G3	G4	G5	G1	G2	G3	G4	G5
Q_1	3460.8	3460.6	3460.6	3460.6	3460.6	3424.4	3350.2	3349.1	3349.0	3349.9
Q_2	2672.6	2670.9	2670.9	2670.9	2670.9	2742.0	2825.9	2856.3	2856.0	2856.0
Q_3	2258.4	2258.0	2258.0	2258.0	2258.0	2231.4	2231.5	2232.8	2232.5	2233.2
Q_4	66.2	63.4	60.8	60.9	60.9	83.4	**-9.6**	55.5	43.8	53.7
Q_5	788.6	788.0	788.0	788.0	788.0	763.9	850.2	850.3	850.3	850.3
Q_6	300.5	177.0	176.8	176.8	176.8	**-1969.4**	**4951.9**	**5008.1**	**5008.9**	**5008.8**
Q_7	41.7	19.7	19.6	19.6	19.6	**-77.6**	**3036.8**	**3084.9**	**3085.3**	**3085.8**

ωB97X

	ω_{har} [cm^{-1}]					ω_{anh} [cm^{-1}]				
Mode	G1	G2	G3	G4	G5	G1	G2	G3	G4	G5
Q_1	3465.4	3466.1	3466.1	3466.2	3466.1	3358.9	3343.9	3343.0	3341.0	3352.1
Q_2	2698.1	2700.6	2700.6	2700.6	2700.6	2640.9	2615.9	2614.3	2614.1	2614.0
Q_3	2249.8	2249.0	2249.0	2249.0	2249.0	2211.7	2220.7	2227.2	2223.6	2228.1
Q_4	86.2	76.6	72.4	74.3	74.5	**-136.3**	**-91.9**	244.8	101.4	65.7
Q_5	786.1	786.1	786.1	786.1	786.1	767.7	755.2	755.3	755.2	756.3
Q_6	231.6	225.5	225.1	225.1	225.1	**-280.9**	**-21.0**	**-23.2**	**-23.2**	**-22.8**
Q_7	36.6	34.1	34.1	34.1	34.1	**-187.7**	**-65.0**	**-58.5**	**-60.3**	**-46.0**

S4 Spurious Oscillations in Low-Frequency Modes of Other Molecules

All the calculations in this section employed the aug-cc-pVDZ basis set.

S4.1 Allyl anion (Q_2 mode)

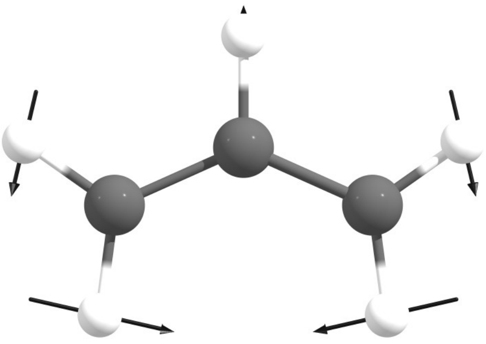

Figure S20: Graphical representation of the Q_2 (A_1) normal mode of allyl anion. Obtained at M06-2X/aug-cc-pVDZ level of theory.

Table S20: Vibrational properties of the Q_2 normal mode of allyl anion obtained with the selected methods: ω_{har} and ω_{anh} - harmonic and anharmonic vibrational frequencies, IR_{har} and IR_{anh} - harmonic and anharmonic IR intensities, RA_{har} and RA_{anh} - harmonic and anharmonic Raman intensities. DFA computations utilized the (250,974) integration grid.

	Q_2 (A_1) mode					
Method	ω_{har} $\left[\frac{1}{cm}\right]$	ω_{anh} $\left[\frac{1}{cm}\right]$	IR_{har} $\left[\frac{km}{mol}\right]$	IR_{anh} $\left[\frac{km}{mol}\right]$	RA_{har} $\left[Å^6\right]$	RA_{anh} $\left[Å^6\right]$
HF	457	459	2.983	3.019	0.705	0.264
ωB97X	427		2.561			
M06-2X	422		2.109			

Table S21: Values of RRMSE (in percents) for the selected property derivatives with respect to the Q_2 normal mode of allyl anion, obtained for the selected DFAs and integration grids.

Method	Grid	d^3E/dQ_2^3	d^4E/dQ_2^4	$d^2\mu_z/dQ_2^2$	$d^3\mu_z/dQ_2^3$
	(99,590)	7.3	12.9	0.2	12.9
ωB97X	(250,974)	0.7	1.9	0.0	1.0
	(750,974)	0.7	1.9	0.0	1.0
	(99,590)	17.7	15.0	0.7	38.3
M06-2X	(250,974)	3.1	7.5	0.0	1.2
	(750,974)	3.1	7.6	0.0	1.2

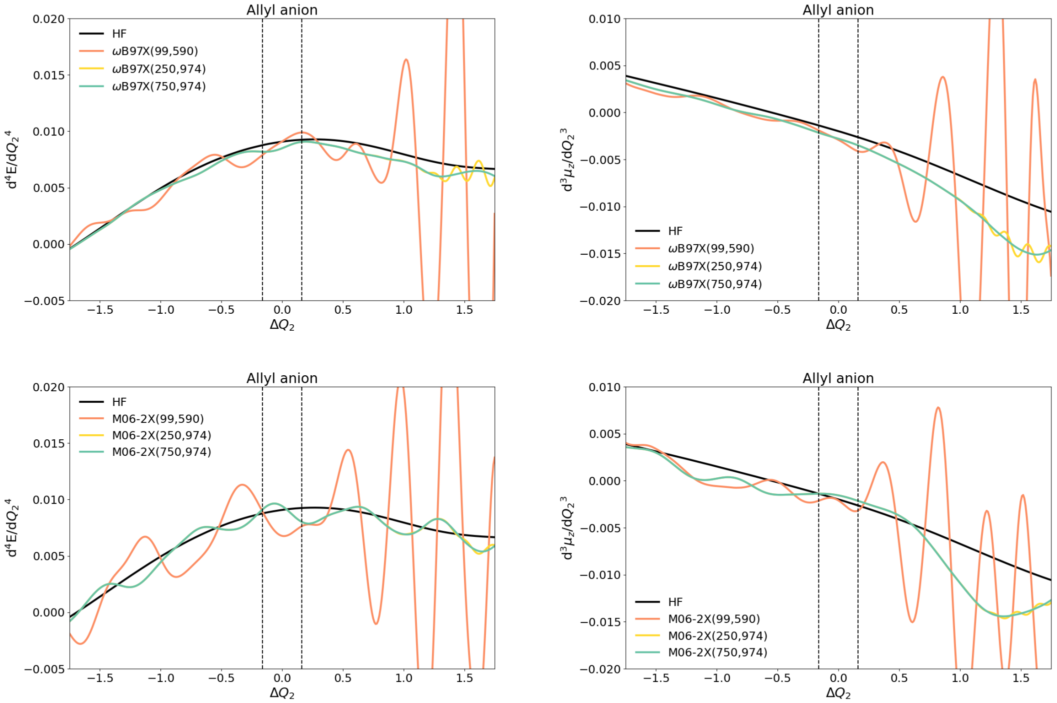

Figure S21: Spurious oscillations in d^4E/dQ_2^4 (left column) and $d^3\mu_z/dQ_2^3$ (right column) for the displacements along the Q_2 normal coordinate of allyl anion. Obtained with ωB97X (top row) and M06-2X (bottom row)

S4.2 Butadiene (Q_2 and Q_3 modes)

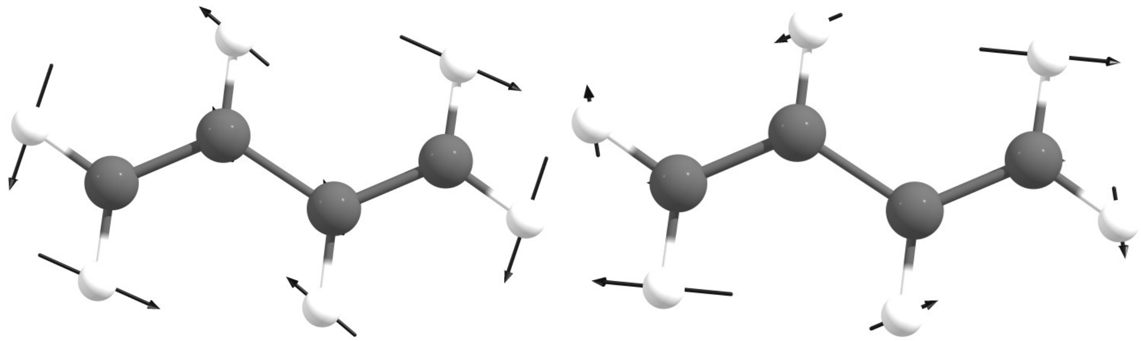

Figure S22: Graphical representation of the Q_2 (B_u, left) and Q_3 (A_g, right) normal modes of butadiene. Obtained at M06-2X/aug-cc-pVDZ level of theory.

Table S22: Vibrational properties of the Q_2 (top panel) and Q_3 (bottom panel) normal modes of butadiene obtained with the selected methods: ω_{har} and ω_{anh} - harmonic and anharmonic vibrational frequencies, IR_{har} and IR_{anh} - harmonic and anharmonic IR intensities, RA_{har} and RA_{anh} - harmonic and anharmonic Raman intensities. DFA computations utilized the (250,974) integration grid.

Q_2 (B_u) mode						
Method	ω_{har} $\left[\frac{1}{\text{cm}}\right]$	ω_{anh} $\left[\frac{1}{\text{cm}}\right]$	IR_{har} $\left[\frac{\text{km}}{\text{mol}}\right]$	IR_{anh} $\left[\frac{\text{km}}{\text{mol}}\right]$	RA_{har} $\left[\text{Å}^6\right]$	RA_{anh} $\left[\text{Å}^6\right]$
HF	319	323	4.028	4.041	0.000	0.000
ωB97X	300		2.979			
M06-2X	299		2.878			

Q_3 (A_g) mode						
Method	ω_{har} $\left[\frac{1}{\text{cm}}\right]$	ω_{anh} $\left[\frac{1}{\text{cm}}\right]$	IR_{har} $\left[\frac{\text{km}}{\text{mol}}\right]$	IR_{anh} $\left[\frac{\text{km}}{\text{mol}}\right]$	RA_{har} $\left[\text{Å}^6\right]$	RA_{anh} $\left[\text{Å}^6\right]$
HF	548	547	0.000	0.000	0.368	0.379
ωB97X	520		0.000			
M06-2X	517		0.000			

Table S23: Values of RRMSE (in percents) for the selected property derivatives with respect to the Q_2 and Q_3 normal modes of allyl anion, obtained for the selected DFAs and integration grids.

Method	Grid	d^3E/dQ_2^3	d^4E/dQ_2^4	$d^3\mu_y/dQ_2^3$	d^3E/dQ_3^3	d^4E/dQ_3^4
	(99,590)	1.4	3.8	18.8	2.4	14.4
ωB97X	(250,974)	0.1	0.3	2.1	0.0	0.7
	(750,974)	0.1	0.3	1.2	0.0	0.5
	(99,590)	6.5	16.0	45.1	6.5	48.3
M06-6X	(250,974)	0.1	0.4	13.5	0.1	8.2
	(750,974)	0.1	0.4	10.9	0.1	8.1

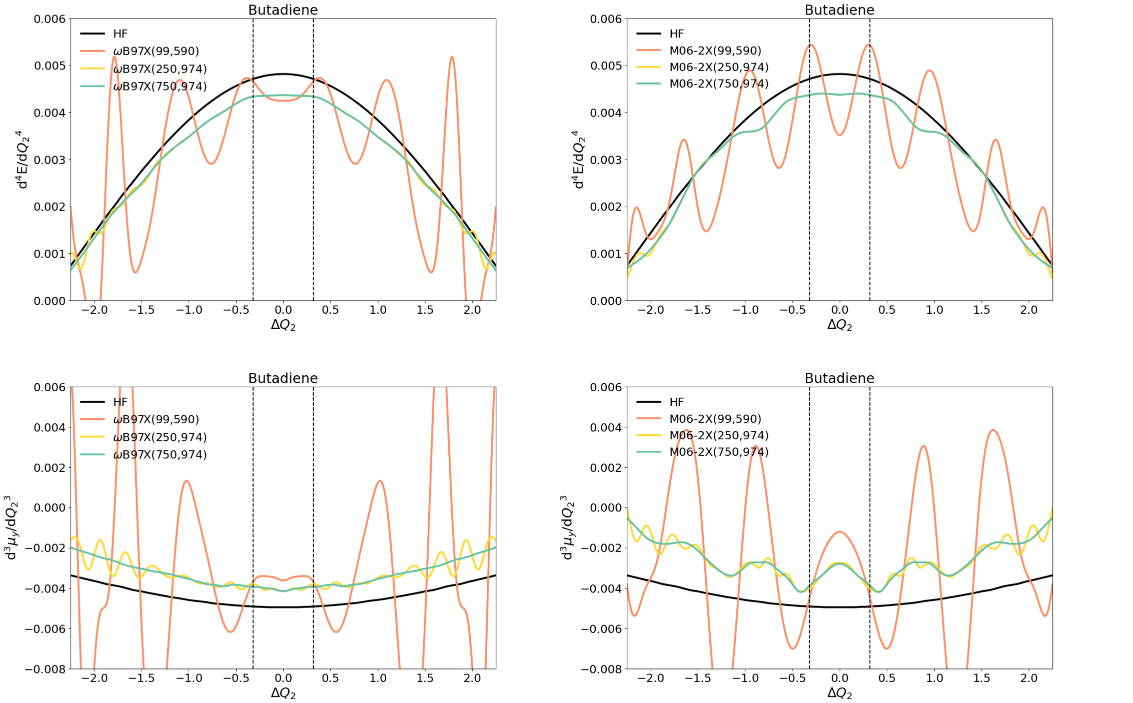

Figure S23: Spurious oscillations in $\mathrm{d}^4E/\mathrm{d}Q_2^4$ (left column) and $\mathrm{d}^3\mu_y/\mathrm{d}Q_2^3$ (right column) for the displacements along the Q_2 normal coordinate of butadiene. Obtained with ωB97X (top row) and M06-2X (bottom row)

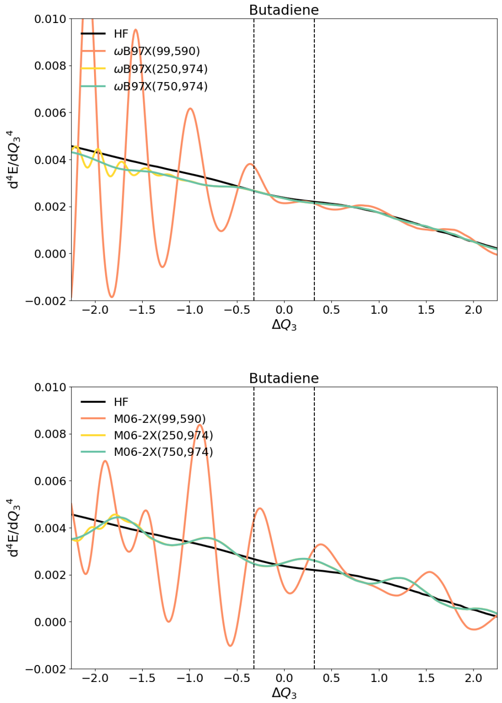

Figure S24: Spurious oscillations in $\mathrm{d}^4E/\mathrm{d}Q_3^4$ for the displacements along the Q_3 normal coordinate of butadiene. Obtained with ωB97X (top row) and M06-2X (bottom row)

S4.3 Cyclobutadiene (Q_4 mode)

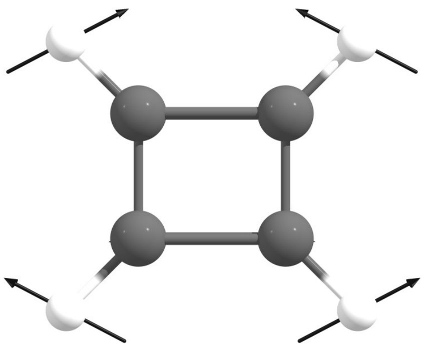

Figure S25: Graphical representation of the Q_4 (B_{2u}) normal mode of cyclobutadiene. Obtained at M06-2X/aug-cc-pVDZ level of theory.

Table S24: Vibrational properties of the Q_4 normal mode of cyclobutadiene obtained with the selected methods: ω_{har} and ω_{anh} - harmonic and anharmonic vibrational frequencies, IR_{har} and IR_{anh} - harmonic and anharmonic IR intensities, RA_{har} and RA_{anh} - harmonic and anharmonic Raman intensities. DFA computations utilized the (250,974) integration grid.

	Q_4 (B_{2u}) mode					
Method	ω_{har} $\left[\frac{1}{\text{cm}}\right]$	ω_{anh} $\left[\frac{1}{\text{cm}}\right]$	IR_{har} $\left[\frac{\text{km}}{\text{mol}}\right]$	IR_{anh} $\left[\frac{\text{km}}{\text{mol}}\right]$	RA_{har} $\left[\text{Å}^6\right]$	RA_{anh} $\left[\text{Å}^6\right]$
HF	804	790	15.557	15.281	0.000	0.000
ωB97X	764		12.255			
M06-2X	749		10.299			

Table S25: Values of RRMSE (in percents) for the selected property derivatives with respect to the Q_4 normal mode of cyclobutadiene, obtained for the selected DFAs and integration grids.

Method	Grid	d^3E/dQ_4^3	d^4E/dQ_4^4	$d^2\mu_y/dQ_4^2$	$d^3\mu_y/dQ_4^3$
	(99,590)	26.9	18.1	46.1	41.2
ωB97X	(250,974)	6.2	11.8	19.4	20.3
	(750,974)	6.1	11.8	19.4	20.3
	(99,590)	3.6	6.3	21.8	20.6
M06-6X	(250,974)	1.5	3.9	1.5	1.5
	(750,974)	1.5	3.6	1.5	1.6

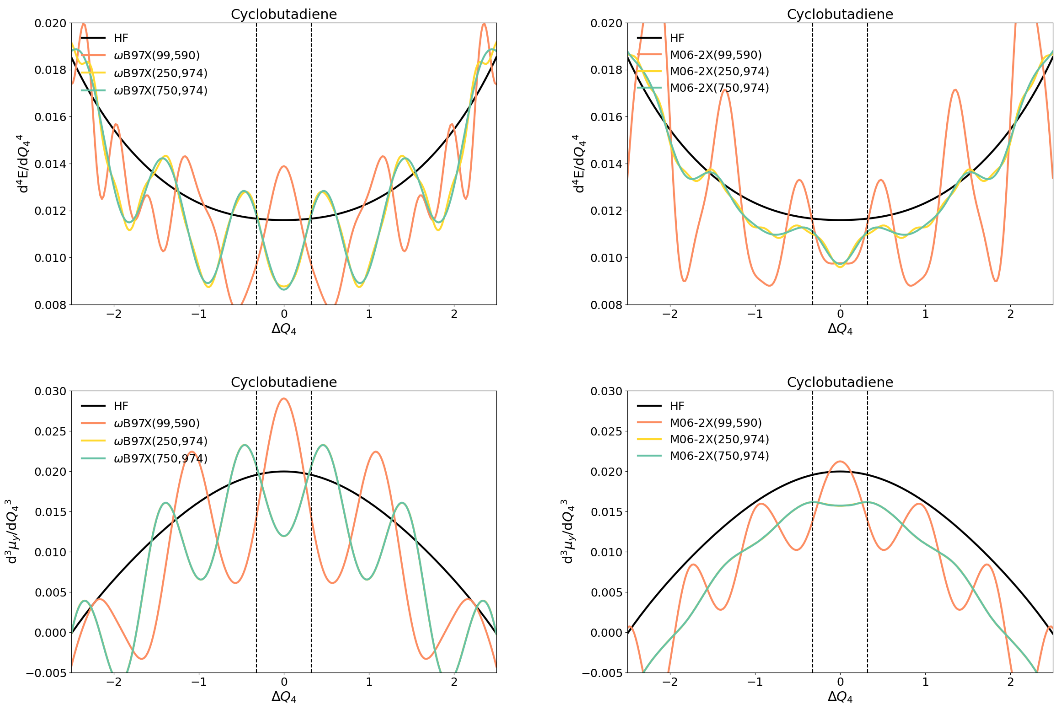

Figure S26: Spurious oscillations in $\mathrm{d}^4E/\mathrm{d}Q_4^4$ (left column) and $\mathrm{d}^3\mu_y/\mathrm{d}Q_4^3$ (right column) for the displacements along the Q_4 normal coordinate of cyclobutadiene. Obtained with ωB97X (top row) and M06-2X (bottom row)

S4.4 Benzene (Q_4 mode)

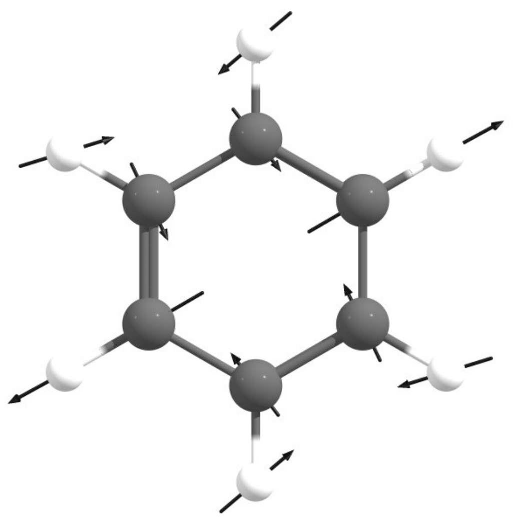

Figure S27: Graphical representation of the Q_4 (E_{2g}) normal mode of benzene. Obtained at M06-2X/aug-cc-pVDZ level of theory.

Table S26: Vibrational properties of the Q_4 normal mode of benzene obtained with the selected methods: ω_{har} and ω_{anh} - harmonic and anharmonic vibrational frequencies, IR_{har} and IR_{anh} - harmonic and anharmonic IR intensities, RA_{har} and RA_{anh} - harmonic and anharmonic Raman intensities. DFA computations utilized the (250,974) integration grid.

	Q_4 (E_{2g}) mode					
Method	ω_{har} $\left[\frac{1}{cm}\right]$	ω_{anh} $\left[\frac{1}{cm}\right]$	IR_{har} $\left[\frac{km}{mol}\right]$	IR_{anh} $\left[\frac{km}{mol}\right]$	RA_{har} $\left[Å^6\right]$	RA_{anh} $\left[Å^6\right]$
HF	659	655	0.000	0.000	0.083	0.075
ωB97X	620		0.000			
M06-2X	613		0.000			

Table S27: Values of RRMSE (in percents) for the selected property derivatives with respect to the Q_4 normal mode of benzene, obtained for the selected DFAs and integration grids.

Method	Grid	d^3E/dQ_4^3	d^4E/dQ_4^4
	(99,590)	43.1	70.8
ωB97X	(250,974)	5.2	14.4
	(750,974)	0.0	6.4
	(99,590)	16.1	28.1
M06-6X	(250,974)	2.1	2.9
	(750,974)	2.2	2.4

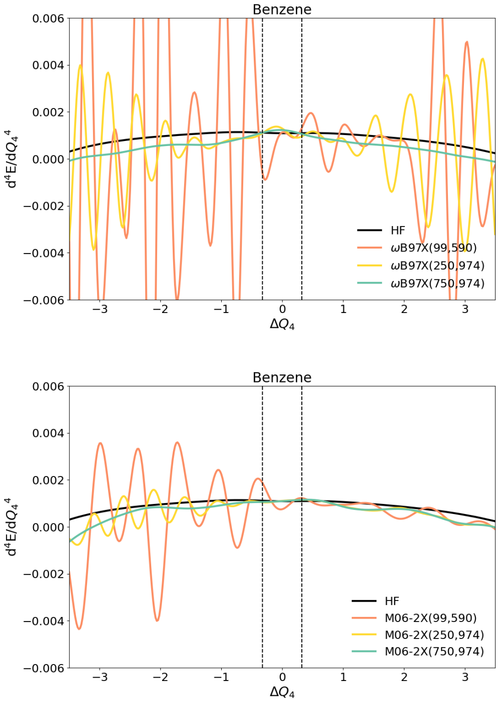

Figure S28: Spurious oscillations in $\mathrm{d}^4E/\mathrm{d}Q_4^4$ for the displacements along the Q_4 normal coordinate of benzene. Obtained with ωB97X (top row) and M06-2X (bottom row)

S4.5 Naphthalene (Q_3 mode)

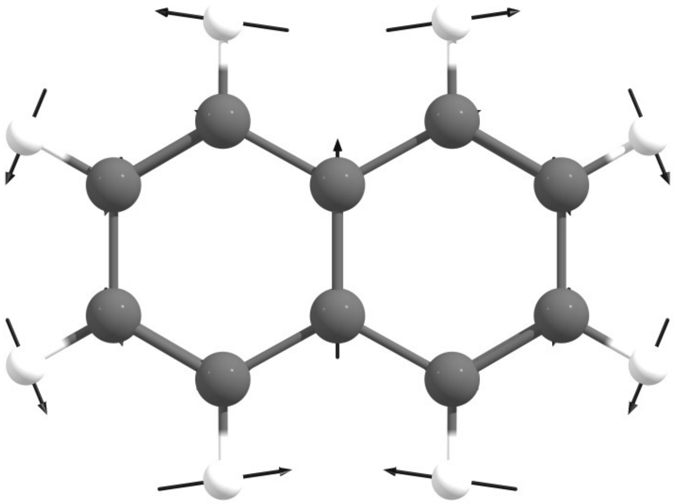

Figure S29: Graphical representation of the Q_3 (B_{1u}) normal mode of naphthalene. Obtained at M06-2X/aug-cc-pVDZ level of theory.

Table S28: Vibrational properties of the Q_3 normal mode of naphthalene obtained with the selected methods: ω_{har} and ω_{anh} - harmonic and anharmonic vibrational frequencies, IR_{har} and IR_{anh} - harmonic and anharmonic IR intensities, RA_{har} and RA_{anh} - harmonic and anharmonic Raman intensities. DFA computations utilized the (250,974) integration grid.

	Q_3 (B_{1u}) mode					
Method	ω_{har} $\left[\frac{1}{cm}\right]$	ω_{anh} $\left[\frac{1}{cm}\right]$	IR_{har} $\left[\frac{km}{mol}\right]$	IR_{anh} $\left[\frac{km}{mol}\right]$	RA_{har} $\left[Å^6\right]$	RA_{anh} $\left[Å^6\right]$
HF	391	373	1.979	2.086	0.000	0.000
B3LYP	372		1.559			
BH&H	380		1.584			
CAM-B3LYP	376		1.601			
ωB97X	371		1.532			
M06-2X	370		1.424			
N12	368		1.435			

Table S29: Values of RRMSE (in percents) for the selected property derivatives with respect to the Q_3 normal mode of naphthalene, obtained for the selected DFAs and integration grids.

Method	Grid	d^3E/dQ_3^3	d^4E/dQ_3^4
	(99,590)	0.7	1.3
B3LYP	(250,974)	0.0	0.1
	(750,974)	0.0	0.1
	(99,590)	6.1	2.2
BH&H	(250,974)	0.1	0.2
	(750,974)	0.1	0.2
	(99,590)	1.1	1.0
CAM-B3LYP	(250,974)	0.1	0.2
	(750,974)	0.1	0.2
	(99,590)	65.1	97.9
ωB97X	(250,974)	15.5	42.4
	(750,974)	8.5	35.0
	(99,590)	72.9	32.9
M06-6X	(250,974)	0.5	0.2
	(750,974)	0.1	0.1
	(99,590)	54.7	102.0
N12	(250,974)	26.6	93.2
	(750,974)	21.5	74.3

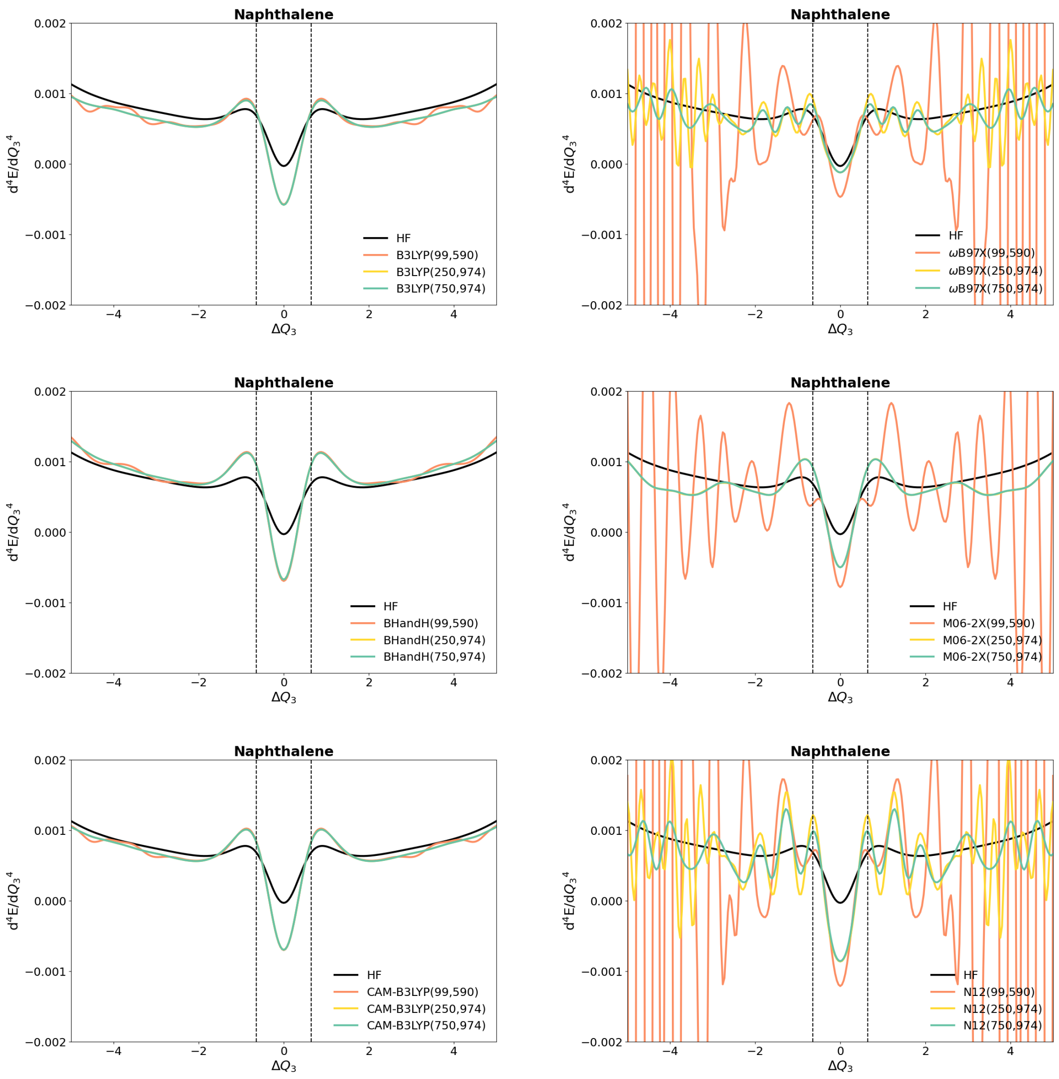

Figure S30: Spurious oscillations in $\mathrm{d}^4E/\mathrm{d}Q_3^4$ for the displacements along the Q_3 normal coordinate of naphthalene. Left column shows the results for B3LYP, BH&H, and CAM-B3LYP, and right column for ωB97X, M06-2X and N12.

S4.6 Phenanthrene (Q_4 mode)

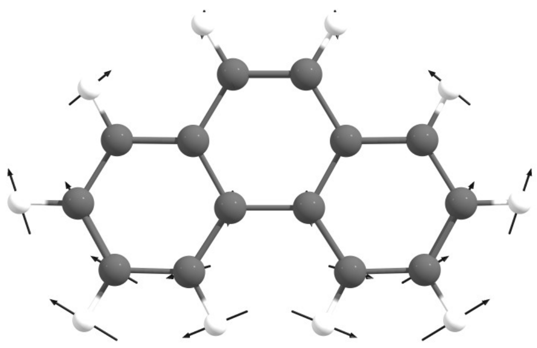

Figure S31: Graphical representation of the Q_4 (A_1) normal mode of phenanthrene. Obtained at M06-2X/aug-cc-pVDZ level of theory.

Table S30: Vibrational properties of the Q_4 normal mode of phenanthrene obtained with the selected methods: ω_{har} and ω_{anh} - harmonic and anharmonic vibrational frequencies, IR_{har} and IR_{anh} - harmonic and anharmonic IR intensities, RA_{har} and RA_{anh} - harmonic and anharmonic Raman intensities. DFA computations utilized the (250,974) integration grid.

	Q_4 (A_1) mode					
Method	ω_{har} $\left[\frac{1}{cm}\right]$	ω_{anh} $\left[\frac{1}{cm}\right]$	IR_{har} $\left[\frac{km}{mol}\right]$	IR_{anh} $\left[\frac{km}{mol}\right]$	RA_{har} $\left[Å^6\right]$	RA_{anh} $\left[Å^6\right]$
HF	263	261	0.495	0.486	0.177	0.165
ωB97X	253		0.4278			
M06-2X	252		0.4133			

Table S31: Values of RRMSE (in percents) for the selected property derivatives with respect to the Q_4 normal mode of phenanthrene, obtained for the selected DFAs and integration grids.

Method	Grid	d^3E/dQ_4^3	d^4E/dQ_4^4	$d^2\mu_z/dQ_4^2$	$d^3\mu_z/dQ_4^3$
	(99,590)	146.8	3030.2	116.6	1136.2
ωB97X	(250,974)	100.6	1716.4	69.5	1086.2
	(750,974)	7.4	41.2	11.0	84.9
	(99,590)	15.0	116.4	37.3	516.8
M06-6X	(250,974)	10.2	188.7	8.8	218.2
	(750,974)	3.6	22.6	1.9	27.3

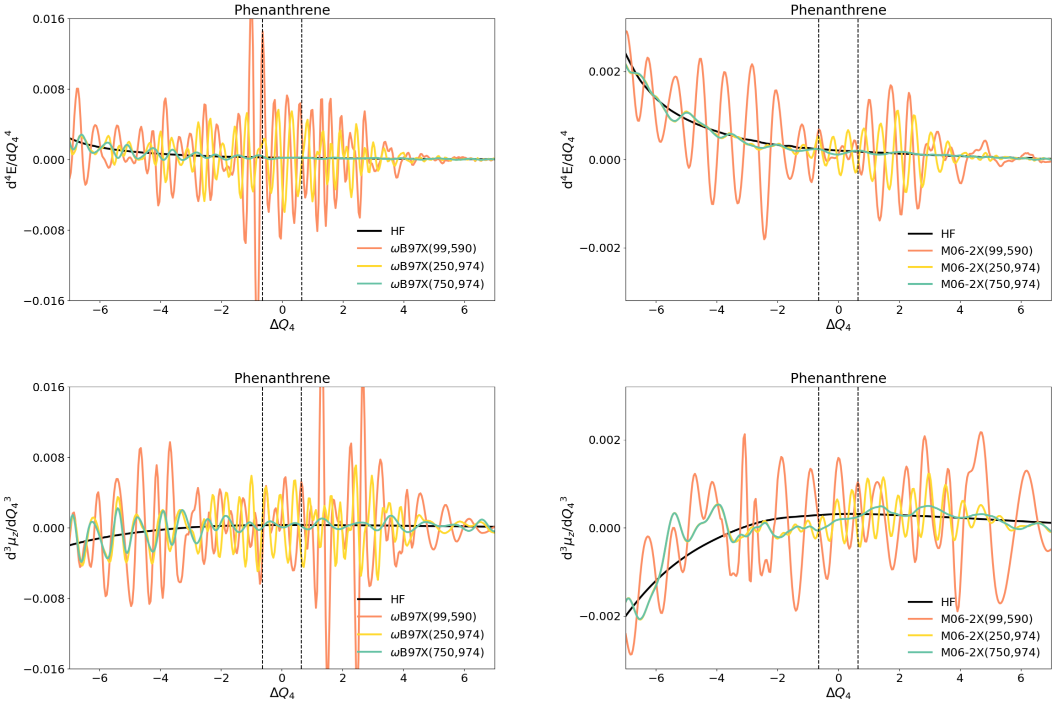

Figure S32: Spurious oscillations in $\mathrm{d}^4E/\mathrm{d}Q_4^4$ (left column) and $\mathrm{d}^3\mu_z/\mathrm{d}Q_4^3$ (right column) for the displacements along the Q_4 normal coordinate of phenanthrene. Obtained with ωB97X (top row) and M06-2X (bottom row)

S4.7 H_2O_2 (Q_1 mode)

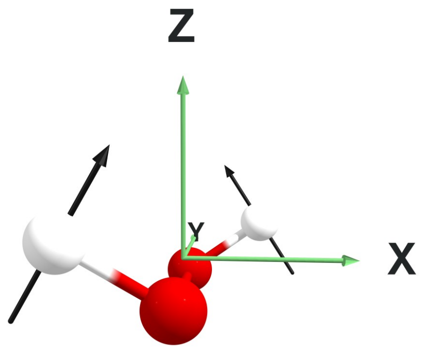

Figure S33: Graphical representation of the Q_1 (A) normal mode of H_2O_2. Obtained at M06-2X/aug-cc-pVDZ level of theory.

Table S32: Vibrational properties of the Q_1 normal mode of H_2O_2 obtained with the selected methods: ω_{har} and ω_{anh} - harmonic and anharmonic vibrational frequencies, IR_{har} and IR_{anh} - harmonic and anharmonic IR intensities, RA_{har} and RA_{anh} - harmonic and anharmonic Raman intensities. DFA computations utilized the (250,974) integration grid.

	Q_1 (A) mode					
Method	ω_{har} $\left[\frac{1}{cm}\right]$	ω_{anh} $\left[\frac{1}{cm}\right]$	IR_{har} $\left[\frac{km}{mol}\right]$	IR_{anh} $\left[\frac{km}{mol}\right]$	RA_{har} $\left[Å^6\right]$	RA_{anh} $\left[Å^6\right]$
HF	412	344	191.151	193.166	0.053	0.063
B3LYP	374		165.073		0.055	
CAM-B3LYP	383		172.425		0.051	
BH&H	416		180.792		0.047	
LC-BLYP	394		186.539		0.047	
PBE	377		148.887		0.049	
PBE0	398		161.896		0.050	
TPSSh	379		157.529		0.056	
ωB97X	395		166.621		0.043	
ωB97XD	398		164.932		0.042	
M06-2X	392		171.213		0.062	
N12	372		172.413		0.047	
MN15	380		173.555		0.062	
MN15-L	392		151.094		0.058	

Table S33: Values of RRMSE (in percents) for the selected property derivatives with respect to the Q_1 normal mode of H_2O_2, obtained for the selected DFAs and integration grids.

Method	Grid	d^2E/dQ_1^2	d^3E/dQ_1^3	d^4E/dQ_1^4
B3LYP	(99,590)	0.1	0.5	0.4
	(250,974)	0.1	1.0	1.1
	(750,974)	0.1	1.0	1.1
	(750,3074)	0.0	0.0	0.0
	(750,5294)	0.0	0.0	0.0
CAM-B3LYP	(99,590)	0.1	0.5	0.3
	(250,974)	0.1	0.9	0.9
	(750,974)	0.1	0.9	0.9
	(750,3074)	0.0	0.0	0.0
	(750,5294)	0.0	0.0	0.0
BH&H	(99,590)	0.1	0.8	0.9
	(250,974)	0.0	0.1	0.0
	(750,974)	0.0	0.1	0.1
	(750,3074)	0.0	0.0	0.0
	(750,5294)	0.0	0.0	0.0
LC-BLYP	(99,590)	0.1	0.2	0.4
	(250,974)	0.1	0.7	0.6
	(750,974)	0.1	0.7	0.6
	(750,3074)	0.0	0.0	0.0
	(750,5294)	0.0	0.0	0.0
PBE	(99,590)	0.2	3.1	3.2
	(250,974)	0.2	2.2	2.8
	(750,974)	0.2	2.2	2.8
	(750,3074)	0.0	0.1	0.1
	(750,5294)	0.0	0.0	0.1
PBE0	(99,590)	0.3	2.6	2.5
	(250,974)	0.2	1.7	2.2
	(750,974)	0.2	1.7	2.2
	(750,3074)	0.0	0.1	0.2
	(750,5294)	0.0	0.0	0.1
TPSSh	(99,590)	0.2	4.4	6.0
	(250,974)	0.2	1.5	1.6
	(750,974)	0.2	1.5	1.6
	(750,3074)	0.0	0.3	0.8
	(750,5294)	0.0	0.0	0.1

Table S34: Values of RRMSE (in percents) for the selected property derivatives with respect to the Q_1 normal mode of H_2O_2, obtained for the selected DFAs and integration grids.

Method	Grid	d^2E/dQ_1^2	d^3E/dQ_1^3	d^4E/dQ_1^4
	(99,590)	1.3	9.4	10.1
	(250,974)	0.2	2.7	3.5
ωB97X	(750,974)	0.2	2.7	3.5
	(750,3074)	0.0	0.6	1.6
	(750,5294)	0.0	0.0	0.1
	(99,590)	1.7	12.2	13.9
	(250,974)	0.3	3.5	4.8
ωB97XD	(750,974)	0.3	3.5	4.8
	(750,3074)	0.1	0.8	2.1
	(750,5294)	0.0	0.0	0.2
	(99,590)	5.6	65.4	51.0
	(250,974)	3.5	13.9	34.4
M06-2X	(750,974)	3.5	13.8	34.1
	(750,3074)	0.2	2.9	6.7
	(750,5294)	0.0	0.1	0.6
	(99,590)	0.8	6.0	13.6
	(250,974)	0.4	3.4	5.3
N12	(750,974)	0.4	3.4	5.3
	(750,3074)	0.1	1.7	4.1
	(750,5294)	0.0	0.1	0.3
	(99,590)	0.4	3.1	6.8
	(250,974)	0.1	0.9	1.5
MN15	(750,974)	0.1	0.9	1.5
	(750,3074)	0.0	0.6	1.1
	(750,5294)	0.0	0.0	0.0
	(99,590)	1.7	16.1	16.3
	(250,974)	1.4	14.6	19.3
MN15-L	(750,974)	1.4	14.6	19.3
	(750,3074)	0.0	0.5	0.8
	(750,5294)	0.0	0.0	0.2

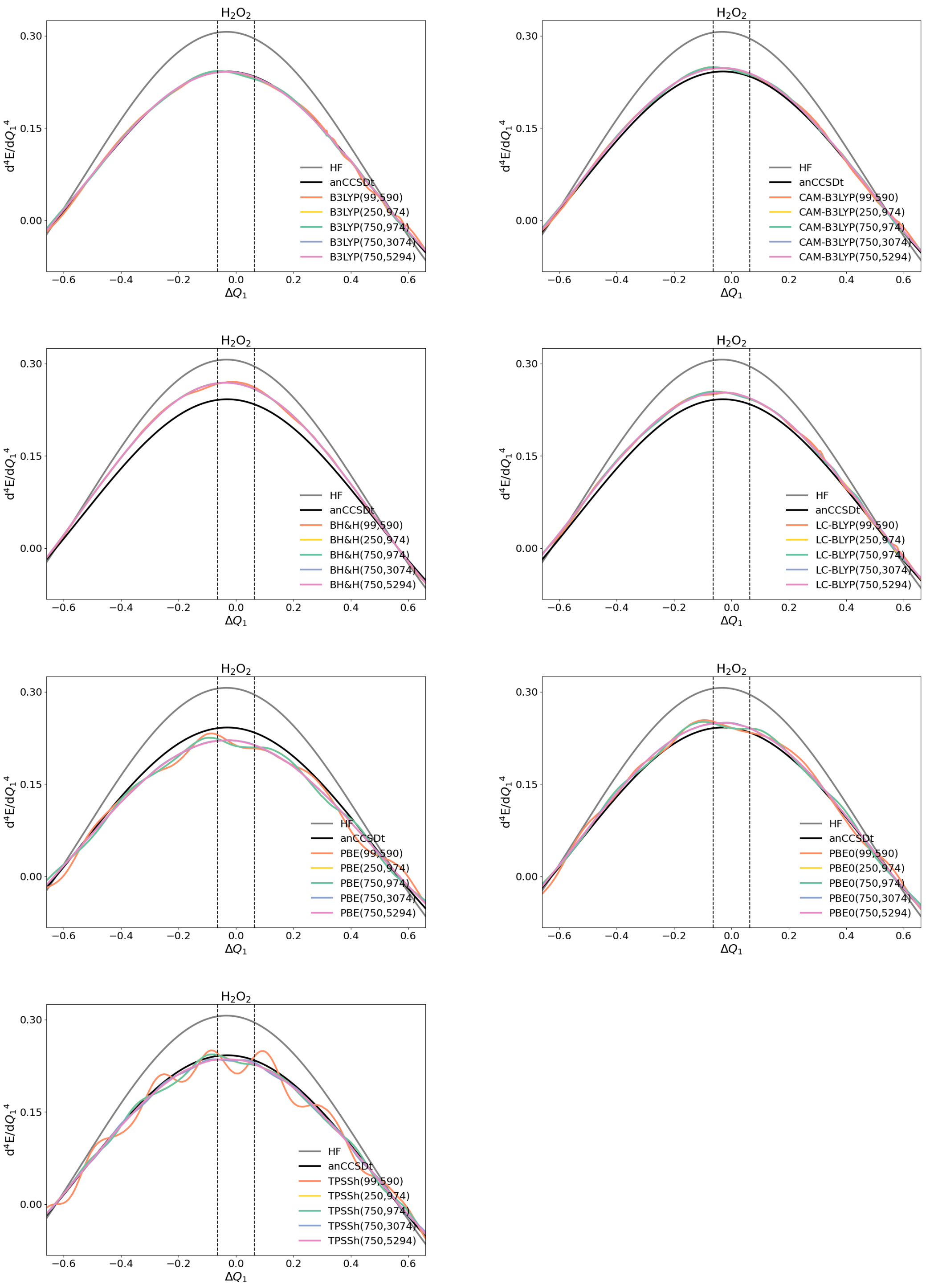

Figure S34: Spurious oscillations in $\mathrm{d}^4E/\mathrm{d}Q_1^4$ for the displacements along the Q_1 normal coordinate of H_2O_2. Left column shows the results for B3LYP, BH&H, PBE, and TPSSh, and right column for CAM-B3LYP, LC-BLYP, and PBE0.

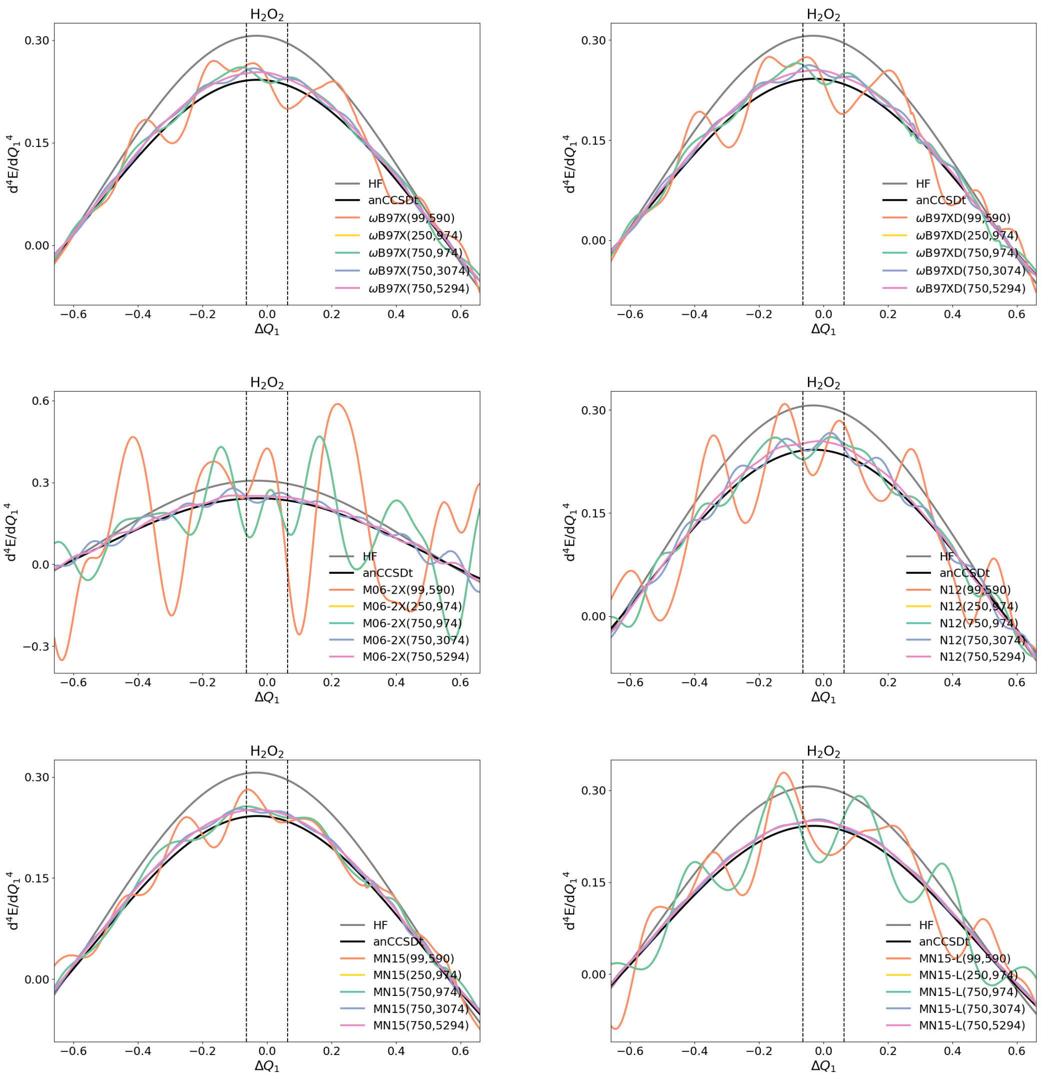

Figure S35: Spurious oscillations in $\mathrm{d}^4E/\mathrm{d}Q_1^4$ for the displacements along the Q_1 normal coordinate of H_2O_2. Left column shows the results for ωB97X, M06-2X, MN15, and right column for ωB97XD, N12, and MN15-L.

S4.8 H_2S_2 (Q_1 mode)

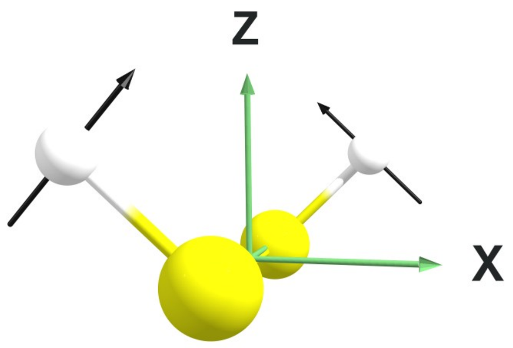

Figure S36: Graphical representation of the Q_1 (A) normal mode of H_2S_2. Obtained at M06-2X/aug-cc-pVDZ level of theory.

Table S35: Vibrational properties of the Q_1 normal mode of H_2S_2 obtained with the selected methods: ω_{har} and ω_{anh} - harmonic and anharmonic vibrational frequencies, IR_{har} and IR_{anh} - harmonic and anharmonic IR intensities, RA_{har} and RA_{anh} - harmonic and anharmonic Raman intensities. DFA computations utilized the (250,974) integration grid.

			Q_1 (A) mode			
Method	ω_{har} $\left[\frac{1}{cm}\right]$	ω_{anh} $\left[\frac{1}{cm}\right]$	IR_{har} $\left[\frac{km}{mol}\right]$	IR_{anh} $\left[\frac{km}{mol}\right]$	RA_{har} $\left[Å^6\right]$	RA_{anh} $\left[Å^6\right]$
HF	447	421	15.918	15.937	0.0427	0.0583
B3LYP	428		12.270		0.0370	
CAM-B3LYP	432		13.689		0.0402	
BH&H	450		15.281		0.0392	
LC-BLYP	438		15.865		0.0480	
ωB97X	433		14.684		0.0346	
ωB97XD	429		13.927		0.0310	
PBE	435		11.857		0.0346	
PBE0	445		13.423		0.0378	
TPSSh	449		12.539		0.0385	
M06-2X	415		13.132		0.0398	
N12	433		13.520		0.0305	
MN15	452		14.369		0.0440	
MN15-L	468		13.535		0.0403	

Table S36: Values of RRMSE (in percents) for the selected property derivatives with respect to the Q_1 normal mode of H_2S_2, obtained for the selected DFAs and integration grids.

Method	Grid	d^2E/dQ_1^2	d^3E/dQ_1^3	d^4E/dQ_1^4
B3LYP	(99,590)	0.6	5.8	6.7
	(250,974)	0.4	2.4	3.5
	(750,974)	0.4	2.4	3.5
	(750,3074)	0.0	0.0	0.1
	(750,5294)	0.0	0.0	0.1
CAM-B3LYP	(99,590)	0.2	1.0	1.4
	(250,974)	0.3	0.4	1.3
	(750,974)	0.3	0.4	1.3
	(750,3074)	0.0	0.0	0.1
	(750,5294)	0.0	0.0	0.1
BH&H	(99,590)	0.4	2.3	4.9
	(250,974)	0.2	0.8	1.2
	(750,974)	0.2	0.8	1.3
	(750,3074)	0.0	0.0	0.1
	(750,5294)	0.0	0.0	0.1
LC-BLYP	(99,590)	0.4	3.6	6.3
	(250,974)	0.3	1.3	1.7
	(750,974)	0.3	1.2	1.5
	(750,3074)	0.0	0.0	0.0
	(750,5294)	0.0	0.0	0.1
PBE	(99,590)	0.3	6.1	3.7
	(250,974)	0.6	35.1	35.4
	(750,974)	0.6	35.2	35.4
	(750,3074)	0.0	0.8	0.8
	(750,5294)	0.0	0.0	0.1
PBE0	(99,590)	0.3	3.9	3.8
	(250,974)	0.5	23.3	28.7
	(750,974)	0.5	23.3	28.8
	(750,3074)	0.0	0.3	0.7
	(750,5294)	0.0	0.0	0.1
TPSSh	(99,590)	2.8	99.4	97.9
	(250,974)	0.6	77.1	76.8
	(750,974)	0.6	77.3	76.8
	(750,3074)	0.1	5.6	5.2
	(750,5294)	0.0	0.1	0.3

Table S37: Values of RRMSE (in percents) for the selected property derivatives with respect to the Q_1 normal mode of H_2S_2, obtained for the selected DFAs and integration grids.

Method	Grid	d^2E/dQ_1^2	d^3E/dQ_1^3	d^4E/dQ_1^4
	(99,590)	2.4	40.9	45.3
	(250,974)	0.4	48.2	56.2
ωB97X	(750,974)	0.4	48.3	56.3
	(750,3074)	0.1	14.9	20.4
	(750,5294)	0.0	0.2	0.3
	(99,590)	2.2	64.2	61.9
	(250,974)	0.9	33.0	38.4
ωB97XD	(750,974)	0.9	33.1	38.7
	(750,3074)	0.2	21.8	28.6
	(750,5294)	0.0	1.4	2.1
	(99,590)	21.9	711.9	817.2
	(250,974)	20.1	497.3	676.4
M06-2X	(750,974)	20.1	499.1	678.4
	(750,3074)	0.8	49.5	59.1
	(750,5294)	0.0	2.1	3.6
	(99,590)	1.3	199.3	203.9
	(250,974)	1.0	129.3	140.2
N12	(750,974)	1.0	129.1	140.1
	(750,3074)	0.2	31.3	44.9
	(750,5294)	0.0	2.2	3.6
	(99,590)	12.0	334.1	353.5
	(250,974)	2.2	55.4	58.6
MN15	(750,974)	2.2	54.9	58.3
	(750,3074)	0.1	1.9	2.2
	(750,5294)	0.0	0.1	0.1
	(99,590)	2.0	39.4	38.6
	(250,974)	0.5	37.8	41.8
MN15-L	(750,974)	0.5	37.7	41.9
	(750,3074)	0.3	11.1	10.5
	(750,5294)	0.0	0.9	1.3

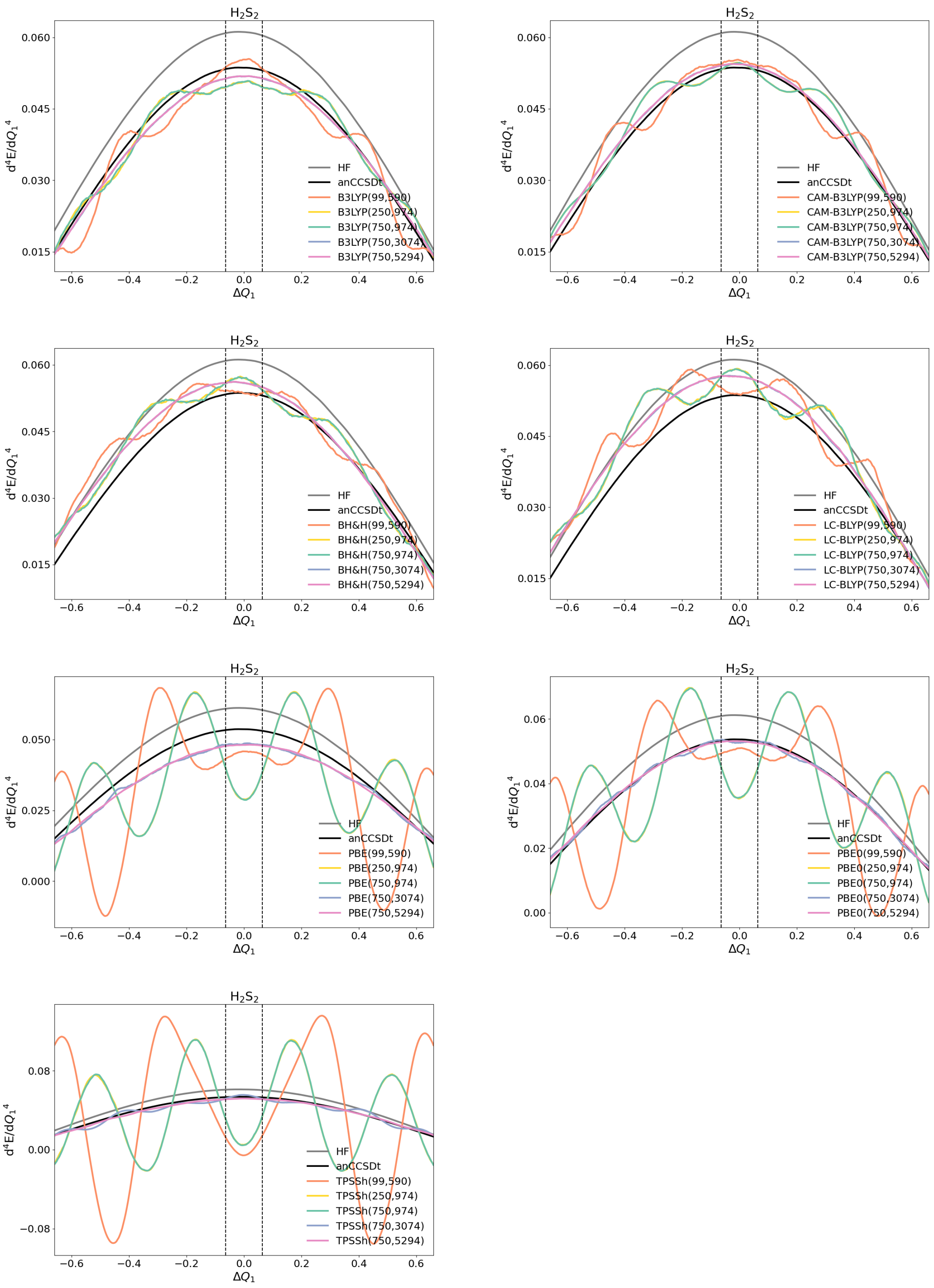

Figure S37: Spurious oscillations in $\mathrm{d}^4E/\mathrm{d}Q_1^4$ for the displacements along the Q_1 normal coordinate of H_2S_2. Left column shows the results for B3LYP, BH&H, PBE, and TPSSh, and right column for CAM-B3LYP, LC-BLYP, and PBE0.

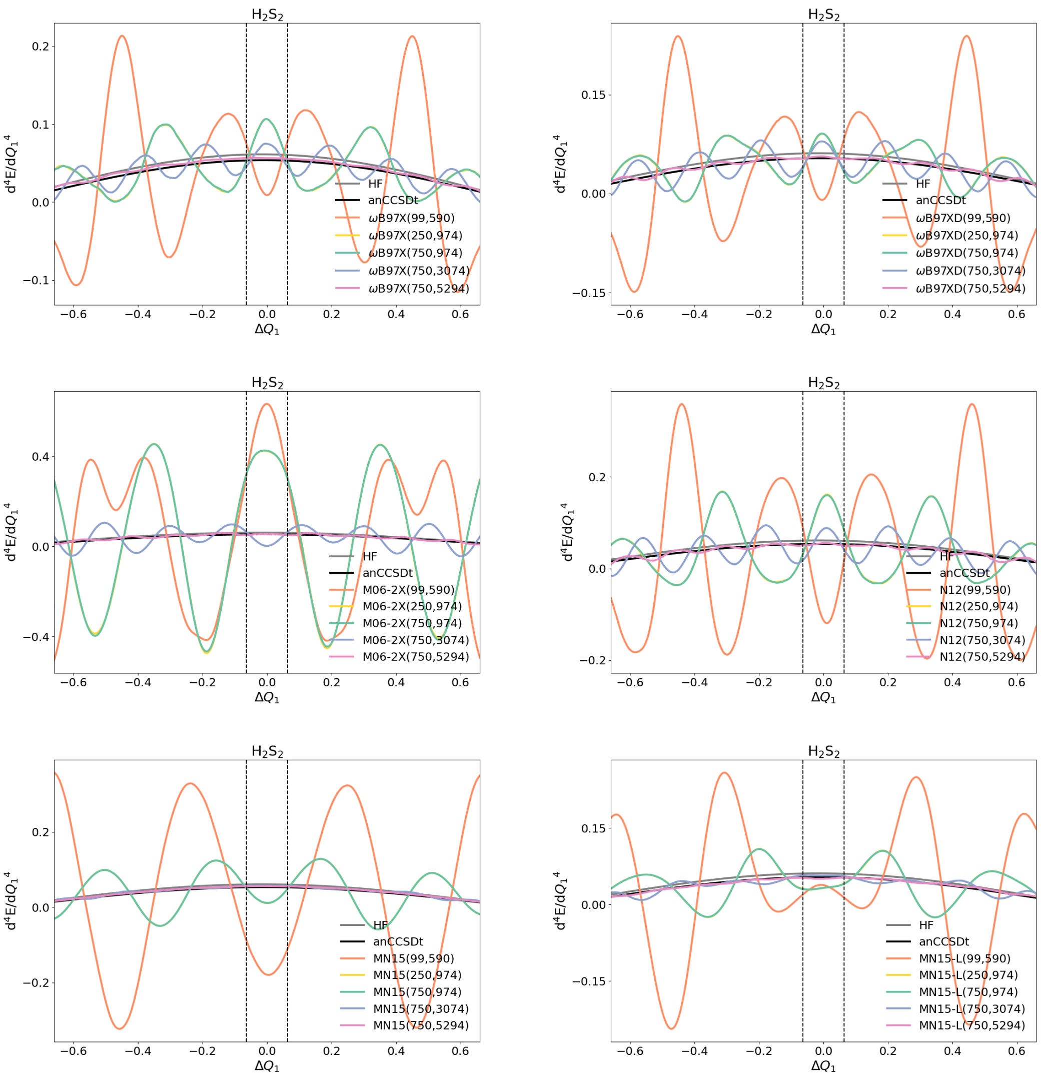

Figure S38: Spurious oscillations in $\mathrm{d}^4E/\mathrm{d}Q_1^4$ for the displacements along the Q_1 normal coordinate of H_2S_2. Left column shows the results for ωB97X, M06-2X, MN15, and right column for ωB97XD, N12, and MN15-L.

References

(1) Luis, J. M.; Duran, M.; Champagne, B.; Kirtman, B. Determination of vibrational polarizabilities and hyperpolarizabilities using field-induced coordinates. *J. Chem. Phys.* **2000**, *113*, 5203–5213.

(2) Harris, C. R.; Millman, K. J.; van der Walt, S. J.; Gommers, R.; Virtanen, P.; Cournapeau, D.; Wieser, E.; Taylor, J.; Berg, S.; Smith, N. J. et al. Array programming with NumPy. *Nature* **2020**, *585*, 357–362.

(3) Virtanen, P.; Gommers, R.; Oliphant, T. E.; Haberland, M.; Reddy, T.; Cournapeau, D.; Burovski, E.; Peterson, P.; Weckesser, W.; Bright, J. et al. SciPy 1.0: fundamental algorithms for scientific computing in python. *Nat. Methods* **2020**, *17*, 261–272.

(4) Meurer, A.; Smith, C. P.; Paprocki, M.; Čertík, O.; Kirpichev, S. B.; Rocklin, M.; Kumar, A.; Ivanov, S.; Moore, J. K.; Singh, S. et al. SymPy: symbolic computing in Python. *PeerJ Comput. Sci.* **2017**, *3*, e103–e129.

(5) Kuo, F. F., Kaiser, J. F., Eds. *System analysis by digital computer*; Wiley: New York, 1966; Vol. 9; Chapter 7.

(6) McClellan, J.; Parks, T. A unified approach to the design of optimum FIR linear-phase digital filters. *IEEE Trans. Circ. Theor.* **1973**, *20*, 697–701.

(7) Bellanger, M. *Digital processing of signals: Theory and practice, 3rd Edition*; Wiley: New York, 2000.

(8) Frisch, M. J.; Trucks, G. W.; Schlegel, H. B.; Scuseria, G. E.; Robb, M. A.; Cheeseman, J. R.; Scalmani, G.; Barone, V.; Petersson, G. A.; Nakatsuji, H. et al. Gaussian 16 Revision C.01. 2016; Gaussian Inc. Wallingford CT.

(9) Epifanovsky, E.; Gilbert, A. T. B.; Feng, X.; Lee, J.; Mao, Y.; Mardirossian, N.; Pokhilko, P.; White, A. F.; Coons, M. P.; Dempwolff, A. L. et al. Software for the frontiers of quantum chemistry: An overview of developments in the Q-Chem 5 package. *J. Chem. Phys.* **2021**, *155*, 084801.

(10) Stanton, J. F.; Gauss, J.; Cheng, L.; Harding, M. E.; Matthews, D. A.; Szalay, P. G. *CFOUR*, Coupled-Cluster techniques for Computational Chemistry, a quantum-chemical program package. With contributions from A.A. Auer, R.J. Bartlett, U. Benedikt, C. Berger, D.E. Bernholdt, S. Blaschke, Y. J. Bomble, S. Burger, O. Christiansen, D. Datta, F. Engel, R. Faber, J. Greiner, M. Heckert, O. Heun, M. Hilgenberg, C. Huber, T.-C. Jagau, D. Jonsson, J. Jusélius, T. Kirsch, K. Klein, G.M. KopperW.J. Lauderdale, F. Lipparini, T. Metzroth, L.A. Mück, D.P. O'Neill, T. Nottoli, D.R. Price, E. Prochnow, C. Puzzarini, K. Ruud, F. Schiffmann, W. Schwalbach, C. Simmons, S. Stopkowicz, A. Tajti, J. Vázquez, F. Wang, J.D. Watts and the integral packages MOLECULE (J. Almlöf and P.R. Taylor), PROPS (P.R. Taylor), ABACUS (T. Helgaker, H.J. Aa. Jensen, P. Jørgensen, and J. Olsen), and ECP routines by A. V. Mitin and C. van Wüllen. For the current version, see http://www.cfour.de.

(11) Dunning, T. H. Gaussian basis sets for use in correlated molecular calculations. I. The atoms boron through neon and hydrogen. *J. Chem. Phys.* **1989**, *90*, 1007–1023.

(12) Kendall, R. A.; Dunning, T. H.; Harrison, R. J. Electron affinities of the first-row atoms revisited. Systematic basis sets and wave functions. *J. Chem. Phys.* **1992**, *96*, 6796–6806.

(13) Wilson, A. K.; Woon, D. E.; Peterson, K. A.; Dunning, T. H. Gaussian basis sets for use in correlated molecular calculations. IX. The atoms gallium through krypton. *J. Chem. Phys.* **1999**, *110*, 7667–7676.

(14) Woon, D. E.; Dunning, T. H. Gaussian basis sets for use in correlated molecular calculations. III. The atoms aluminum through argon. *J. Chem. Phys.* **1993**, *98*, 1358–1371.

(15) Woon, D. E.; Dunning, T. H. Gaussian basis sets for use in correlated molecular calculations. IV. Calculation of static electrical response properties. *J. Chem. Phys.* **1994**, *100*, 2975–2988.

(16) Feller, D. The role of databases in support of computational chemistry calculations. *J. Comput. Chem.* **1996**, *17*, 1571–1586.

(17) Schuchardt, K. L.; Didier, B. T.; Elsethagen, T.; Sun, L.; Gurumoorthi, V.; Chase, J.; Li, J.; Windus, T. L. Basis set exchange: a community database for computational sciences. *J. Chem. Inf. Model.* **2007**, *47*, 1045–1052.

(18) Pritchard, B. P.; Altarawy, D.; Didier, B.; Gibson, T. D.; Windus, T. L. New basis set exchange: an open, up-to-date resource for the molecular sciences community. *J. Chem. Inf. Model.* **2019**, *59*, 4814–4820.

(19) Becke, A. D. Density-functional exchange-energy approximation with correct asymptotic behavior. *Phys. Rev. A* **1988**, *38*, 3098–3100.

(20) Lee, C.; Yang, W.; Parr, R. G. Development of the Colle-Salvetti correlation-energy formula into a functional of the electron density. *Phys. Rev. B* **1988**, *37*, 785–789.

(21) Perdew, J. P.; Burke, K.; Ernzerhof, M. Generalized gradient approximation made simple. *Phys. Rev. Lett.* **1996**, *77*, 3865–3868.

(22) Adamo, C.; Barone, V. Toward reliable adiabatic connection models free from adjustable parameters. *Chem. Phys. Lett.* **1997**, *274*, 242–250.

(23) Adamo, C.; Barone, V. Toward reliable density functional methods without adjustable parameters: The PBE0 model. *J. Chem. Phys.* **1999**, *110*, 6158–6170.

(24) Becke, A. D. Density-functional thermochemistry. III. The role of exact exchange. *J. Chem. Phys.* **1993**, *98*, 5648–5652.

(25) Stephens, P. J.; Devlin, F. J.; Chabalowski, C. F.; Frisch, M. J. Ab initio calculation of vibrational absorption and circular dichroism spectra using density functional force fields. *J. Phys. Chem.* **1994**, *98*, 11623–11627.

(26) Rohrdanz, M. A.; Herbert, J. M. Simultaneous benchmarking of ground- and excited-state properties with long-range-corrected density functional theory. *J. Chem. Phys.* **2008**, *129*, 034107.

(27) Becke, A. D. Density-functional thermochemistry. V. Systematic optimization of exchange-correlation functionals. *J. Chem. Phys.* **1997**, *107*, 8554–8560.

(28) Tawada, Y.; Tsuneda, T.; Yanagisawa, S.; Yanai, T.; Hirao, K. A long-range-corrected time-dependent density functional theory. *J. Chem. Phys.* **2004**, *120*, 8425–8433.

(29) Grimme, S. Semiempirical GGA-type density functional constructed with a long-range dispersion correction. *J. Comput. Chem.* **2006**, *27*, 1787–1799.

(30) Yanai, T.; Tew, D. P.; Handy, N. C. A new hybrid exchange–correlation functional using the Coulomb-attenuating method (CAM-B3LYP). *Chem. Phys. Lett.* **2004**, *393*, 51–57.

(31) Chai, J.-D.; Head-Gordon, M. Systematic optimization of long-range corrected hybrid density functionals. *J. Chem. Phys.* **2008**, *128*, 084106.

(32) Zhao, Y.; Truhlar, D. The M06 suite of density functionals for main group thermochemistry, thermochemical kinetics, noncovalent interactions, excited states, and transition elements: two new functionals and systematic testing of four M06-class functionals and 12 other functionals. *Theor. Chem. Acc.* **2008**, *120*, 215–241.

(33) Zhao, Y.; Truhlar, D. G. A new local density functional for main-group thermochemistry, transition metal bonding, thermochemical kinetics, and noncovalent interactions. *J. Chem. Phys.* **2006**, *125*, 194101.

(34) Chai, J.-D.; Head-Gordon, M. Long-range corrected hybrid density functionals with damped atom–atom dispersion corrections. *Phys. Chem. Chem. Phys.* **2008**, *10*, 6615–6620.

(35) Zhao, Y.; Truhlar, D. G. Density Functional for Spectroscopy: No long-range self-interaction error, good performance for Rydberg and charge-transfer states, and better performance on average than B3LYP for ground states. *J. Phys. Chem. A* **2006**, *110*, 13126–13130.

(36) Lin, Y.-S.; Li, G.-D.; Mao, S.-P.; Chai, J.-D. Long-range corrected hybrid density functionals with improved dispersion corrections. *J. Chem. Theory Comput.* **2013**, *9*, 263–272.

(37) Mardirossian, N.; Head-Gordon, M. Mapping the genome of meta-generalized gradient approximation density functionals: The search for B97M-V. *J. Chem. Phys.* **2015**, *142*, 074111.

(38) Peverati, R.; Zhao, Y.; Truhlar, D. G. Generalized gradient approximation that recovers the second-order density-gradient expansion with optimized across-the-board performance. *J. Phys. Chem. Lett.* **2011**, *2*, 1991–1997.

(39) Mardirossian, N.; Head-Gordon, M. ωB97M-V: A combinatorially optimized, range-separated hybrid, meta-GGA density functional with VV10 nonlocal correlation. *J. Chem. Phys.* **2016**, *144*, 214110.

(40) Peverati, R.; Truhlar, D. G. Communication: A global hybrid generalized gradient approximation to the exchange-correlation functional that satisfies the second-order density-gradient constraint and has broad applicability in chemistry. *J. Chem. Phys.* **2011**, *135*, 191102.

(41) Mardirossian, N.; Head-Gordon, M. ωB97X-V: A 10-parameter, range-separated hybrid, generalized gradient approximation density functional with nonlocal correlation, designed by a survival-of-the-fittest strategy. *Phys. Chem. Chem. Phys.* **2014**, *16*, 9904–9924.

(42) Peverati, R.; Truhlar, D. G. Improving the accuracy of hybrid meta-GGA density functionals by range separation. *J. Phys. Chem. Lett.* **2011**, *2*, 2810–2817.

(43) Tao, J.; Perdew, J. P.; Staroverov, V. N.; Scuseria, G. E. Climbing the density functional ladder: nonempirical meta–generalized gradient approximation designed for molecules and solids. *Phys. Rev. Lett.* **2003**, *91*, 146401.

(44) Peverati, R.; Truhlar, D. G. M11-L: A local density functional that provides improved accuracy for electronic structure calculations in chemistry and physics. *J. Phys. Chem. Lett.* **2012**, *3*, 117–124.

(45) Perdew, J. P.; Ruzsinszky, A.; Csonka, G. I.; Constantin, L. A.; Sun, J. Workhorse semilocal density functional for condensed matter physics and quantum chemistry. *Phys. Rev. Lett.* **2009**, *103*, 026403.

(46) Peverati, R.; Truhlar, D. G. An improved and broadly accurate local approximation to the exchange–correlation density functional: The MN12-L functional for electronic structure calculations in chemistry and physics. *Phys. Chem. Chem. Phys.* **2012**, *14*, 13171–13174.

(47) Staroverov, V. N.; Scuseria, G. E.; Tao, J.; Perdew, J. P. Comparative assessment of a new nonempirical density functional: Molecules and hydrogen-bonded complexes. *J. Chem. Phys.* **2003**, *119*, 12129–12137.

(48) Peverati, R.; Truhlar, D. G. Screened-exchange density functionals with broad accuracy for chemistry and solid-state physics. *Phys. Chem. Chem. Phys.* **2012**, *14*, 16187–16191.

(49) Brémond, E.; Adamo, C. Seeking for parameter-free double-hybrid functionals: The PBE0-DH model. *J. Chem. Phys.* **2011**, *135*, 024106.

(50) Brémond, E.; Sancho-García, J. C.; Pérez-Jiménez, A. J.; Adamo, C. Communication: Double-hybrid functionals from adiabatic-connection: The QIDH model. *J. Chem. Phys.* **2014**, *141*, 031101.

(51) Grimme, S. Semiempirical hybrid density functional with perturbative second-order correlation. *J. Chem. Phys.* **2006**, *124*, 034108.

(52) Yu, H. S.; He, X.; Li, S. L.; Truhlar, D. G. MN15: A Kohn–Sham global-hybrid exchange–correlation density functional with broad accuracy for multi-reference and single-reference systems and noncovalent interactions. *Chem. Sci.* **2016**, *7*, 5032–5051.

(53) Schwabe, T.; Grimme, S. Towards chemical accuracy for the thermodynamics of large molecules: new hybrid density functionals including non-local correlation effects. *Phys. Chem. Chem. Phys.* **2006**, *8*, 4398–4401.

(54) Yu, H. S.; He, X.; Truhlar, D. G. MN15-L: A new local exchange-correlation functional for Kohn–Sham density functional theory with broad accuracy for atoms, molecules, and solids. *J. Chem. Theory Comput.* **2016**, *12*, 1280–1293.

(55) Heyd, J.; Scuseria, G. E.; Ernzerhof, M. Hybrid functionals based on a screened Coulomb potential. *J. Chem. Phys.* **2003**, *118*, 8207–8215.

(56) Heyd, J.; Scuseria, G. E.; Ernzerhof, M. Erratum: "Hybrid functionals based on a screened Coulomb potential" [J. Chem. Phys. 118, 8207 (2003)]. *J. Chem. Phys.* **2006**, *124*, 219906.

(57) Sun, J.; Ruzsinszky, A.; Perdew, J. P. Strongly constrained and appropriately normed semilocal density functional. *Phys. Rev. Lett.* **2015**, *115*, 036402.

(58) Krukau, A. V.; Vydrov, O. A.; Izmaylov, A. F.; Scuseria, G. E. Influence of the exchange screening parameter on the performance of screened hybrid functionals. *J. Chem. Phys.* **2006**, *125*, 224106.

(59) Hui, K.; Chai, J.-D. SCAN-based hybrid and double-hybrid density functionals from models without fitted parameters. *J. Chem. Phys.* **2016**, *144*, 044114.

(60) Takahasi, H.; Mori, M. Double exponential formulas for numerical integration. *Publ. Res. Inst. Math. Sci.* **1974**, *9*, 721–741.

(61) Mori, M. Discovery of the double exponential transformation and its developments. *Publ. Res. Inst. Math. Sci.* **2005**, *41*, 897–935.

(62) Mitani, M. An application of double exponential formula to radial quadrature grid in density functional calculation. *Theor. Chem. Acc.* **2011**, *130*, 645–669.

(63) Mitani, M.; Yoshioka, Y. Numerical integration of atomic electron density with double exponential formula for density functional calculation. *Theor. Chem. Acc.* **2012**, *131*, 1–15.

(64) Treutler, O.; Ahlrichs, R. Efficient molecular numerical integration schemes. *J. Chem. Phys.* **1995**, *102*, 346–354.

(65) Murray, C. W.; Handy, N. C.; Laming, G. J. Quadrature schemes for integrals of density functional theory. *Mol. Phys.* **1993**, *78*, 997–1014.

(66) Mura, M. E.; Knowles, P. J. Improved radial grids for quadrature in molecular density-functional calculations. *J. Chem. Phys.* **1996**, *104*, 9848–9858.

(67) Lindh, R.; Malmqvist, P.; Gagliardi, L. Molecular integrals by numerical quadrature. I. Radial integration. *Theor. Chem. Acc.* **2001**, *106*, 178–187.

(68) Vydrov, O. A.; Van Voorhis, T. Nonlocal van der Waals density functional: The simpler the better. *J. Chem. Phys.* **2010**, *133*, 244103.

(69) Dasgupta, S.; Herbert, J. S. Standard Grids for high-precision integration of modern density functionals: SG-2 and SG-3 . *J. Comput. Chem.* **2017**, *38*, 869–882.

(70) Savitzky, A.; Golay, M. J. Smoothing and differentiation of data by simplified least squares procedures. *Anal. Chem.* **1964**, *36*, 1627–1639.

(71) Medved, M.; Stachová, M.; Jacquemin, D.; André, J. M.; Perpète, E. A. A generalized Romberg differentiation procedure for calculation of hyperpolarizabilities. *J. Mol. Struct. (Theochem)* **2007**, *847*, 39–46.

(72) Zaleśny, R.; Medved', M.; Sitkiewicz, S. P.; Matito, E.; Luis, J. M. Can Density Functional Theory Be Trusted for High-Order Electric Properties? The Case of Hydrogen-Bonded Complexes. *J. Chem. Theory Comput.* **2019**, *15*, 3570–3579.
